# The genetic basis of hydrocephalus: genes, pathways, mechanisms, and global impact

**DOI:** 10.1186/s12987-024-00513-z

**Published:** 2024-03-04

**Authors:** Andrew T. Hale, Hunter Boudreau, Rishi Devulapalli, Phan Q. Duy, Travis J. Atchley, Michael C. Dewan, Mubeen Goolam, Graham Fieggen, Heather L. Spader, Anastasia A. Smith, Jeffrey P. Blount, James M. Johnston, Brandon G. Rocque, Curtis J. Rozzelle, Zechen Chong, Jennifer M. Strahle, Steven J. Schiff, Kristopher T. Kahle

**Affiliations:** 1 Department of Neurosurgery, University of Alabama at Birmingham, FOT Suite 1060, 1720 2ndAve, Birmingham, AL 35294 UK; 2 Heersink School of Medicine, University of Alabama at Birmingham, Birmingham, AL UK; 3 Department of Neurosurgery, University of Virginia School of Medicine, Charlottesville, VA USA; 4 Division of Pediatric Neurosurgery, Monroe Carell Jr. Children’s Hospital, Vanderbilt University School of Medicine, Nashville, TN USA; 5 Neuroscience Institute, University of Cape Town, Cape Town, South Africa; 6 Division of Pediatric Neurosurgery, Red Cross War Memorial Children’s Hospital, University of Cape Town, Cape Town, South Africa; 7 Division of Pediatric Neurosurgery, Children’s of Alabama, University of Alabama at Birmingham, Birmingham, AL UK; 8 Heflin Center for Genomics, University of Alabama at Birmingham, Birmingham, AL UK; 9 Division of Pediatric Neurosurgery, St. Louis Children’s Hospital, Washington University in St. Louis, St. Louis, MO USA; 10 Department of Neurosurgery, Yale University School of Medicine, New Haven, CT USA; 11 Department of Neurosurgery, Massachusetts General Hospital, Harvard Medical School, Boston, MA USA

## Abstract

Hydrocephalus (HC) is a heterogenous disease characterized by alterations in cerebrospinal fluid (CSF) dynamics that may cause increased intracranial pressure. HC is a component of a wide array of genetic syndromes as well as a secondary consequence of brain injury (intraventricular hemorrhage (IVH), infection, etc.) that can present across the age spectrum, highlighting the phenotypic heterogeneity of the disease. Surgical treatments include ventricular shunting and endoscopic third ventriculostomy with or without choroid plexus cauterization, both of which are prone to failure, and no effective pharmacologic treatments for HC have been developed. Thus, there is an urgent need to understand the genetic architecture and molecular pathogenesis of HC. Without this knowledge, the development of preventive, diagnostic, and therapeutic measures is impeded. However, the genetics of HC is extraordinarily complex, based on studies of varying size, scope, and rigor. This review serves to provide a comprehensive overview of genes, pathways, mechanisms, and global impact of genetics contributing to all etiologies of HC in humans.

## Introduction

Hydrocephalus (HC) is characterized by aberrant cerebrospinal fluid (CSF) dynamics (with or without ventricular dilation) that can lead to increased intracranial pressure. When left untreated, HC may be fatal and cause severe impairment in neurodevelopment. While classical theories of CSF posited that CSF is produced predominantly in the lateral ventricles via the choroid plexus and flows through the foramina of Monroe, third ventricle, cerebral aqueduct, and the fourth ventricle where it is disseminated through the central canal of the spinal cord and the subarachnoid space to be reabsorbed by arachnoid granulations [1], this model is no longer considered dogmatic [2]. Anatomical disruption of CSF flow and/or CSF pulsatility may result in a buildup of CSF to be classified as obstructive or non-communicating HC. However, HC can be communicating (i.e., no obvious anatomical blockade of absorption or obstruction), the result from increased production of CSF in response to injury, impaired absorption from the subarachnoid space, or result from defects in cortical development. These insults, in turn, may lead to ventricular dilation, among other potential and highly debated pathophysiologic mechanisms. Importantly, the global burden of HC is high [3], with significant morbidity and mortality regardless of treatment [4]. However, the genetic and mechanistic basis of HC remains poorly understood, largely due to the genetic complexity and phenotypic heterogeneity of the disease as well as cost of large-scale human genetics studies.

HC is a component of a wide-array of genetic syndromes [5], a secondary consequence of brain injury (intraventricular hemorrhage (IVH), infection, etc.) [6, 7], and a component of many central nervous system congenital abnormalities (i.e., neural tube defects, Chiari malformation, etc.) with a number of comorbid phenotypes including epilepsy and autism, among others. HC is a highly polygenic disease [8–10], with genes of varying functions and mechanisms conferring risk to the disease. The current treatments for HC are surgical interventions such as insertion of a ventricular (-peritoneal, atrial, etc.) shunt or endoscopic third ventriculostomy (ETV), which may be combined with choroid plexus cauterization (CPC) [6, 11]. While many studies have evaluated the efficacy and cost of these procedures [12], long-term morbidity of HC remains high and both treatments are prone to failure [13, 14]. Furthermore, while clinical trials have attempted pharmacological strategies to treat HC [15], no pharmacological treatment has been successful. In addition, HC may present in adulthood as normal pressure hydrocephalus (NPH). A more sophisticated and detailed understanding the genetic architecture and molecular pathogenesis of HC may lead to development of targeted pharmacologic treatments.

While numerous studies have aimed to identify causative genetic mechanisms leading to HC, largely based on isolated human case studies and murine models [5], critical limitations include cost, patient/family recruitment, number of patients (small by population-genetics’ standards), individual variant validation (typically de novo mutations), and very important species differences between model-organisms and human disease. Proposed pathophysiological mechanisms of HC include impaired development of the neural stem cell niche [16–20], abnormal ciliated ependymal cells [21–23], disruption of the ventricular zone [24, 25], and primary alterations in CSF absorption and/or secretion [26–29]. However, our understanding of these mechanisms is derived from varied model systems, which do not always accurately recapitulate the genetic and pathophysiological basis of human HC. Furthermore, there is increasing evidence that germline genetic variation contributes to risk of HC [5, 8, 10, 30]; however, most cases of HC remain genetically undefined and clinical genetic testing is rarely performed.

Elucidation of the genetic architecture of both shared and etiology-specific forms of HC may uncover pathophysiological mechanisms and correlate genetic risk factors with clinical and surgical outcomes, with the potential to directly influence surgical counseling and clinical management. While many genes have been implicated in the pathogenesis of HC in humans, the study designs, approaches, and levels of evidence identifying and validating these genetic findings vary greatly. Uncovering the genetic basis of HC relies on many factors, but most importantly on the clinical phenotype in question because HC rarely occurs in isolation. Comorbid phenotypes (neural tube defects, primary structural brain disorders, epilepsy, cognitive delay, etc.), and antecedent injuries – IVH and/or infection (meningitis, intracranial abscess, and/or sepsis), alone and in concert, confound most classical approaches to understanding genetic disease. Advancing our understanding of HC genetics, therefore, will necessitate understanding the extent to which co-occurring phenotypes are present and integration of multiple molecular and genetic data. Furthermore, elucidation of human-specific molecular mechanisms necessitates study in human tissue representative of the diverse populations HC affects. Here we summarize genetic studies of HC in humans and offer suggestions for advancing the field forward.

## Methods

### Search criteria

The US National Library of Medicine PubMed database and the Online Mendelian Inheritance in Man (OMIM) were queried for English-language studies using Title/Abstract, MeSH headings, key words, and genetic descriptors relevant to genetic causes of HC and ventriculomegaly. The OMIM database was used as an additional adjunct database as well. Our search terms are included below. Duplicates identified across multiple databases were identified. We strictly adhered to PRISMA guidelines [31].

Our PubMed search syntax included the following: (HC[Title/Abstract]) OR (Ventriculomegaly[Title/Abstract]); ((HC[MeSH Major Topic]) OR (Ventriculomegaly[MeSH Major Topic])) AND ("mendelian" OR "de novo" OR "functional genomics" OR "whole exome sequencing" OR "whole-genome sequencing" OR "genotyping" OR "genotype" OR "microarray" OR "genome-wide association study" OR "genome wide association study" OR "GWAS" OR "transcriptome wide association study" OR "transcriptome-wide association study" OR "TWAS" OR "gene expression" OR "copy number variation" OR "insertion" OR "deletion" OR "mosaic" OR "mosaicism" OR "genetic variation" OR "consanguineous" OR "consanguinity" OR "autosomal recessive" OR "autosomal dominant" OR "x-linked recessive" OR "x-linked dominant" OR "inherited" OR "inheritance" OR "non-coding" OR "coding" OR "co-expression" OR "germline" OR "linkage" OR "linkage disequilibrium" OR "genetic counseling" OR "syndrome" OR "syndromic" OR "genetic testing" OR "aqueductal stenosis" OR "obstructive HC" OR "acquired HC" OR "congenital HC" OR "proteomics" OR "proteomic" OR "metabolomic" OR "metabolomics" OR "methylation" OR "mutation" OR "genetic deficiency" OR "gain of function" OR "gain-of-function" OR "loss of function" OR "loss-of-function" OR "molecular"[Title/Abstract]).

We next queried the Online Mendelian Inheritance in Man (OMIM) database [32] using the search terms: “HC” or “ventriculomegaly” to identify genetic disease of which HC is a component. The search returned 671 entries which were manually reviewed. Duplicates within the OMIM database were excluded (n = 95). The resulting search query resulted in 3,709 studies.

### Inclusion and exclusion criteria

Records (n = 3,709) from the above search were initially evaluated via abstract and screened for exclusion criteria: (1) Records published before 1970; (2) no genetic data of any kind; (3) no HC diagnosis; or (4) animal subjects. A total of 2,652 studies were excluded. Full text screening of the remaining papers (n = 1,057) was then screened for inclusion criteria. The second round of screening was carried out by full text review (n = 1,057). The same exclusion criteria were applied, while inclusion criteria were implemented: (1) Records published after 1970; (2) pediatric cohort (0–18 years of age); (3) primary genetic analysis; (4) confirmed diagnosis of human HC; and (5) human subjects. The final records were assessed for eligibility and records unavailable in English were excluded (n = 2). The final studies included (n = 327) were then evaluated for the methodology and type of genetic analysis performed. The papers included in our study (n = 327) were then subject to secondary analyses to assess for (1) change in number of publications over time; (2) geographic and ethnic associations of HC; (3) size of study; (4) central nervous system and non-central nervous system phenotypic associations. Figures were created using BioRender.

### Author affiliation and subject country of origin

Authors’ institutional affiliation was obtained via PubMed’s “Affiliations” tab within the respective research articles PubMed webpage. Authors were then cross-referenced via Google search to increase validity of institutional affiliation at the time the study was performed. Articles were individually queried for the country of origin of patients with HC. If not explicitly stated, it was assumed that the patients were from the same country as the senior author’s affiliation. The total number of cases were tallied and tabulated on a world map using OpenStreetMap.

## Results

**Fig. 1:**
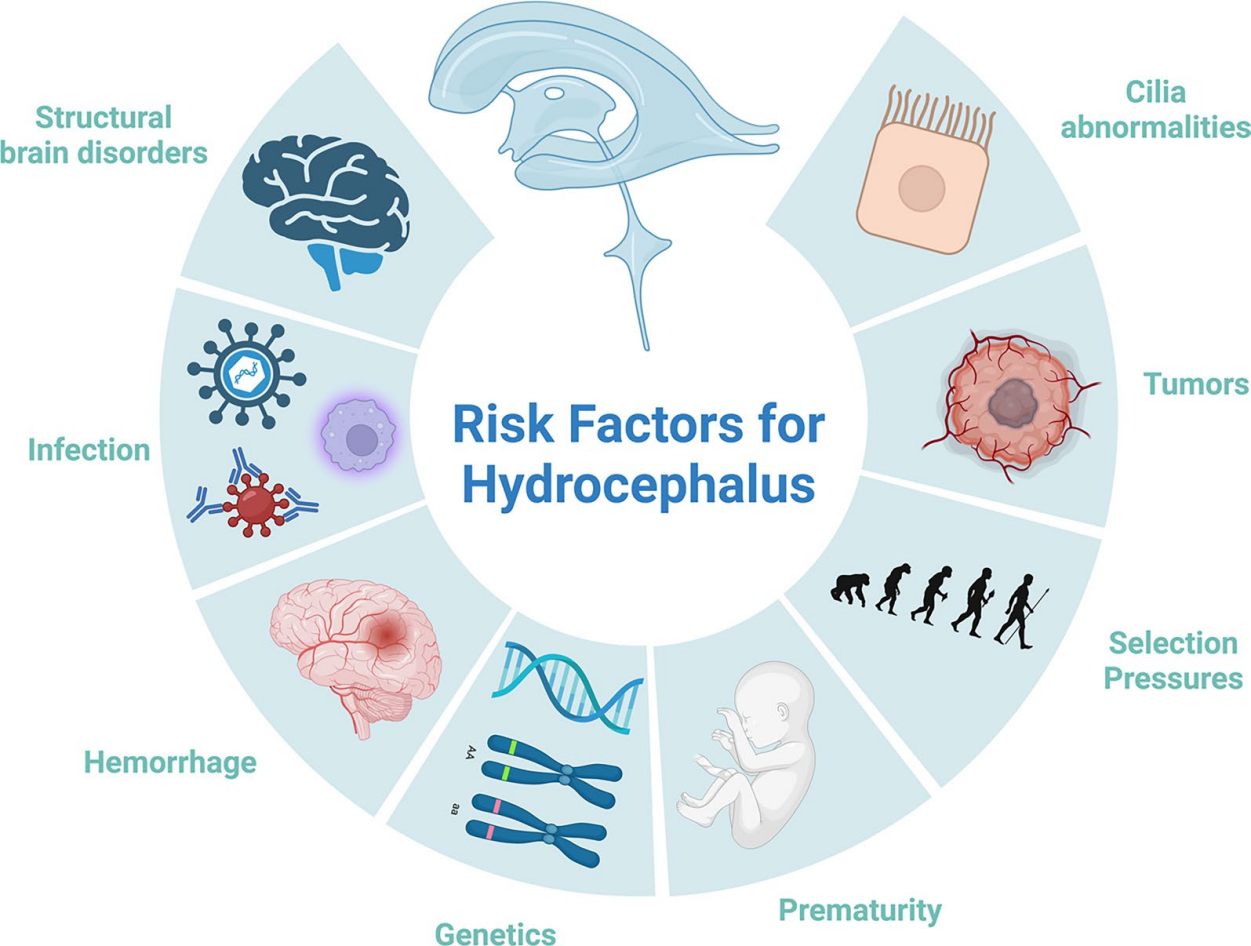
Factors contributing to the development of hydrocephalus in humans

**Fig. 2:**
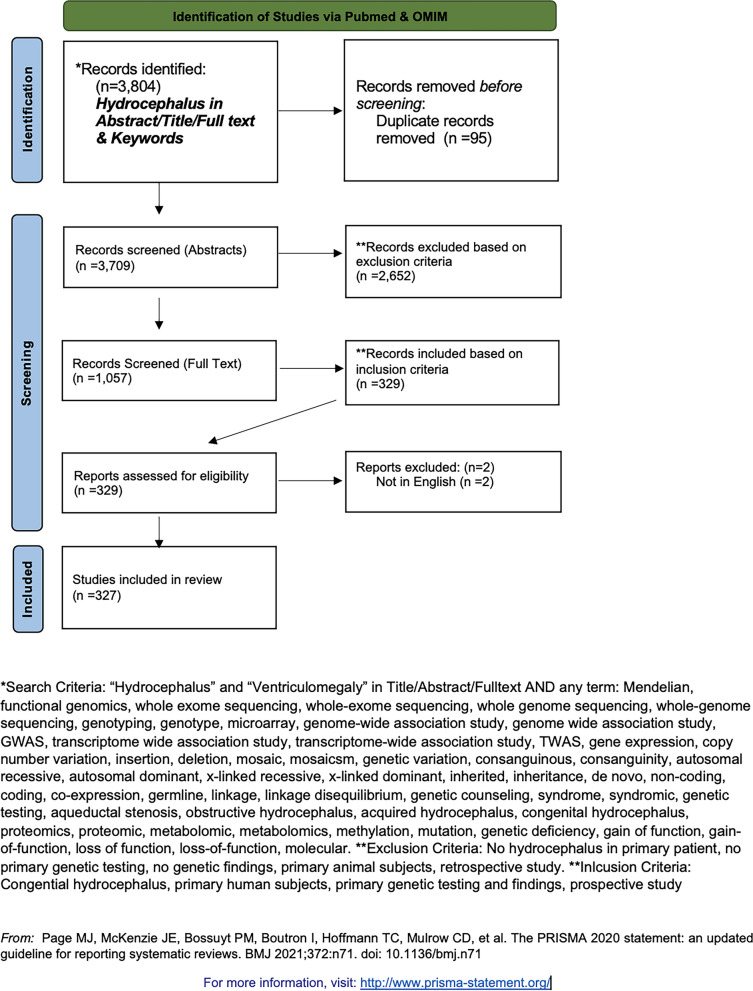
PRISMA flowchart outlining literature search to identify genes, mutations, and genetic mechanisms contributing to hydrocephalus in humans

HC in humans can be caused by or is secondary to several factors including structural brain disorders, cilia abnormalities, brain tumors resulting in CSF obstruction requiring CSF diversion, neural tube defects, prematurity and germinal matrix fragility, neonatal systemic and CNS infections, intracranial hemorrhage, evolutionary selection pressures, and ‘genetic’ anomalies, classically thought as Mendelian disorders (Fig. 1). Thus, we conducted a systematic review of human genetic studies of HC to quantify and summarize the current state of genetic contributions to HC of various etiologies (Fig. 2). However, genetic susceptibility confers risk to all these preceding factors as well as to HC directly. Thus, understanding the pleiotropic effect of genes on both risk factors and development of HC is needed and requires highly detailed phenomics analysis [33]. Here, we summarize all genetic studies of *human* HC across the age spectrum, including discussion of animal models of HC only as corroborating findings of genes and pathways identified in humans where there is a reasonable degree of evolutionary conservation. We believe this is essential as regulation of CSF and brain development is highly divergent across evolution, necessitating clarification and specificity of how genetics plays a role in human disease. Categories are defined a priori based on either phenotypic, molecular, or known genetic classifications. While many forms of HC can reasonably be classified into multiple categories, we attempt to simplify the groupings below.

### Hydrocephalus secondary to aqueductal stenosis (AS)

Human genetics studies of HC secondary to aqueductal stenosis (AS) are summarized in Table 1. Fifteen unique gene mutations on 11 chromosomes inherited in both X-linked and autosomal patterns underlying HC secondary to AS have been identified. These genes include protocadherin 9 (*PCDH9),* immunoglobulin superfamily containing leucine rich repeat 2 *(ISLR2),* ATPase Na + /K + transporting subunit alpha 3 *(ATP1A3),* L1 cell adhesion molecule *(L1CAM),* FA complementation group C *(FAC),* fibroblast growth factor receptor 3 *(FGFR3),* solute carrier family 12 member 6 *(SLC12A6),* crumbs cell polarity complex component 2 *(CRB2),* Bardet-Biedl syndrome 7 *(BBS7),* podocin gene *(NPHS2),* multiple PDZ domain crumbs cell polarity complex component *(MPDZ),* laminin subunit beta 1 *(LAMB1),* alpha glucosidase *(GAA),* A-Disintegrin and Metalloproteinase with Thrombospondin motifs like 2 *(ADAMTSL2),* collagen type IV alpha 2 chain *(COL4A2)*. A duplication in the Xp22.33 region and deletions of the long arm of chromosome 9, 12q22-q23.1, mutation in SRY-box transcription factor 2 (*SOX2*) gene, and mutation in the solute carrier family 12-member 7 (*SLC12A7)* gene were also identified.

**Table 1:** Aqueductal stenosis

Citation	Title	Author affiliation	Case #	Ancestry	Study design	CNS phenotype	Non-CNS phenotype	Type of hydrocephalus	Genetic methodology	Genetic analysis	Inheritance	Genetic Findings
Alazami et al., 2019 [129]	A novel ISLR2-linked autosomal recessive syndrome of congenital hydrocephalus, arthrogryposis and abdominal distension	King Faisal Specialist Hospital and Research Center, Riyadh, Saudi Arabia	2 Subjects, 8 Controls	Saudi	Case series	Generalized hypotonia, diminished reflexes	Arthrogryposis and abdominal distension	Obstructive	WES	Autozygome analysis; Variant analysis	AR	13q21.32 (c.652 T > C, p.Y218H in PCDH9); 15q24.1 (c.1660delT, p.W554Gfs*40 in ISLR2)
Allocco et al., 2019 [130]	Recessive Inheritance of Congenital Hydrocephalus with Other Structural Brain Abnormalities Caused by Compound Heterozygous Mutations in ATP1A3	Yale University, New Haven, CT, United States	1 Subject, 2 Parents	Caucasian	Case study	Open schizencephaly, type 1 Chiari malformation, and dysgenesis of the corpus callosum	–	Obstructive	WES	CNV, Sanger sequencing	AR	19q13.2 (p.R19C in exon 2 and p.R463C in exon 11 of ATP1A3)
Chassaing et al., 2007 [131]	Germinal mosaicism and familial recurrence of a SOX2 mutation with highly variable phenotypic expression extending from AEG syndrome to absence of ocular involvement	Hôpital Purpan, Toulouse, France	1 Subject, 1 Control	–	Case study	Brain malformations, corpus callosum hypoplasia	Ocular dysgenesis, male genital tract malformations, postnatal growth retardation, and facial dysmorphic features	Obstructive	TGS	–	AD	3q26.33 (deletion within SOX2)
Cox et al., 1997 [132]	VACTERL with hydrocephalus in twins due to Fanconi anemia (FA): mutation in the FAC gene	Royal Postgraduate Medical School, Hammersmith Hospital, London, United Kingdom	2 Subjects, 2 Parents	South African Ashkenazi Jew	Case study	Isolated hydrocephalus	Absent thumb, pericardial effusion, tracheo-esophageal fistula, pulmonary dysgenesis, intestinal malrotation, ectopic kidney, tetralogy of fallot	Obstructive	TGS	–	AR	9q22.32 (IVS4 + 4 A to T splice mutation in intron 4 of FAC)
De Keersmaecker et al., 2013 [133]	Prenatal diagnosis of MPPH syndrome	University Hospitals, Leuven, Belgium	1 Subject	–	Case study	Polymicrogyria	Postaxial polydactyly	Obstructive	Cytogenetics, TGS	aCGH	–	–
Escobar et al., 2009 [134]	Significant phenotypic variability of Muenke syndrome in identical twins	St. Vincent Children's Hospital, Indianapolis, Indiana, USA	2 Subjects	–	Case study	Coronal craniosynostosis, porencephalic cyst, and absence of the corpus callosum	Bilateral sensorineural hearing loss, tracheoesophageal fistula, asd, vsd	Obstructive	TGS	–	De novo	4p16.3 (c.C749G, p.P250R in FGFR3 gene)
Gomy et al., 2008 [135]	Two new Brazilian patients with Gómez-López-Hernández syndrome: reviewing the expanded phenotype with molecular insights	School of Medicine of Ribeirão Preto, University of São Paulo, Ribeirão Preto, Brazil	2 Subjects	Brazilian	Case study	Craniosynostosis, craniofacial anomalies, trigeminal anesthesia, cerebellar ataxia, intellectual disability, and rhombencephalosynapsis	Scalp alopecia, developmental delay	Obstructive	TGS	Direct sequencing	-	No pathogenic mutations
Isik et al., 2018 (78) [136]	Clinical and genetic features of L1 syndrome patients: Definition of two novel mutations	Faculty of Medicine, Ege University, Izmir, Turkey	2 Subjects	-	Case series	Intellectual disability, microcephaly, spasticity	Developmental delay, broad forehead, hypertelorism, low set ears, long philtrum, bilateral adducted thumbs, atrial septal defect	Obstructive	Molecular analysis, unspecified	De-novo mutation analysis, Segregation analysis	X-linked	Xq28 (c.3166 + 1G > A; c.749delG, p.S250Tfs*51)
Jin et al., 2019 [137]	SLC12A ion transporter mutations in sporadic and familial human congenital hydrocephalus	Yale University School of Medicine, New Haven, CT, USA	2 Subjects	-	Case series	Agenesis of the corpus callosum, and schizencephaly	-	Obstructive	WES	CNV	De novo	15q14 (c.C1814T, p.Pr05L in SLC12A6); 5p15.33 (SLC12A7 deletion)
Jouet et al., 1993 [138]	Refining the genetic location of the gene for X linked hydrocephalus within Xq28	University of Cambridge	4 Subjects	-	Case series	Seizures, intellectual disability, callosal agenesis, aqueduct stenosis, spastic paraparesis	Adducted thumbs	Obstructive	Genotyping	Two point/multipoint linkage analysis	X-linked recessive	Xq28 (HSAS gene proximal to DXS605 & coincident with DXS52)
Khattab et al., 2011 [139]	A de novo 3.54 Mb deletion of 17q22-q23.1 associated with hydrocephalus: a case report and review of literature	Yale University School of Medicine, New Haven, Connecticut 06520–8064, USA	1 Subject	-	Case study	Generalized hypotonia	Sutural separation with full anterior fontanel, small palpebral fissures, hypertelorism, low-set ears, micrognathia, downturned corners of the mouth, arachnodactyly of fingers and toes, contractures of joints	Obstructive	TGS, cytogenetics	aCGH; FISH	De novo	17q22-q23.1 deletion
Lamont et al., 2016 [140]	Expansion of phenotype and genotypic data in CRB2-related syndrome	Alberta Children's Hospital Research Institute, University of Calgary, Calgary, Alberta, Canada	5 Subjects	-	Case series	Hypoplastic cerebellum, Subependymal heterotopias, hypertonia	Bilateral echogenic kidneys, hypoplastic right lung, hypoplastic right pulmonary artery, dextroposition of the heart, ASD defect, low visual acuity, irregular retinal pigmentation, mild optic atrophy	Obstructive	WES	NGS, Sanger sequencing, SNP genotyping array	AR, De novo, maternal	9q33.3 (CRB2 mutations (p.C620S; p.R628C; p.C629S; p.G1036Afs*42; p.R1248Q; p.W498C; p.R633W; p.E643A; p.W759X; p.N800K; p.D1076A; p.C1098Sfs*53; p.R1115C; p.C1129R)); 4q27 (BBS7 mutation (p.R238Efs*59)); 1q25.2 (NPHS2 mutation (p.R229Q))
Lyonnet et al., 1992 [141]	The gene for X-linked hydrocephalus maps to Xq28, distal to DXS52	Hôpital des Enfants-Malades, Paris, France	58 Subjects	French, German	Case series	Intellectual disability and spasticity	Abnormal flexion deformity Of the thumbs	-	Haplotyping	Pairwise and multipoint linkage analysis	X-linked	HSAS1 localized to Xq28 and distal to DXS52
Maurya et al., 2021 [142]	A case report of Arnold Chiari type 1 malformation in acromesomelic dwarf infant	Seth Gordhandas Sunderdas Medical College and King Edward Memorial Hospital, Mumbai, Maharashtra, India	1 Subject	-	Case study	Arnold Chiari type 1 malformation, atrophy of both lentiform nuclei, paucity of white matter in bilateral occipital regions, thinning of the corpus callosum	Acromesomelic dwarfism	Obstructive	WGS	NGS	AR	4p16.3 (c.G1138A, p.G380R in exon 9 of FGFR3); 9p23 (c.G394A, p.G132S in exon 5 of MPDZ); 7q31.1 (c.T4133A, p.L1378H in exon 27 of LAMB1); 17q25.3 (c.A1G, p.M1V in exon 2 of GAA)
Porayette et al., 2013 [143]	Novel mutations in geleophysic dysplasia type 1	Boston Children's Hospital, Harvard Medical School, Boston, MA, USA	1 Subject	-	Case study	Isolated hydrocephalus	Large head, prominent flat forehead, hypertelorism, wide mouth with long thin lips, full cheeks, downturned corners of the mouth, short palpebral fissures, long flat philtrum, grooved tongue with tongue-tie, low-set ears, small nose with depressed nasal bridge, short neck, mild abdominal distension, symmetrical shortening of all extremities, aortic stenosis, pulmonary valve stenosis	Obstructive	TGS	-	AD	9q34.2 (c.1934G > A, p.R645H in exon 13 and mutation in intron 8) of ADAMTSL2)
Serville et al., 1993 [144]	Prenatal exclusion of X-linked hydrocephalus-stenosis of the aqueduct of Sylvius sequence using closely linked DNA markers	Unité de Recherches sur les Handicaps Génétiques de l'Enfant INSERM U-12, Hôpital des Enfants-Malades, Paris, France	2 Subject	-	Case series	Cortex thinning, spasticity, cerebral palsy, intellectual disability, corpus callosum agenesis, aqueductal stenosis	Bilateral adducted thumbs	Obstructive	Southern blotting, DNA probes; autoradiography	Linkage analysis	X-linked recessive	Xq28 region linkage with the HSAS locus
Strain et al., 1994 [145]	Genetic heterogeneity in X-linked hydrocephalus: linkage to markers within Xq27.3	Human Genetics Unit, University of Edinburgh, Western General Hospital, UK	4 Subjects, Controls used	-	Case series	Septum pellucidum and corpus callosum agenesis, aqueductal stenosis	Adducted thumbs	Obstructive	Chromosomal banding	Two-point/multipoint linkage analysis	-	Linkage to DXS548 and FRAXA loci in Xq27.3
Tzschach et al., 2012 [146]	Interstitial 9q34.11-q34.13 deletion in a patient with severe intellectual disability, hydrocephalus, and cleft lip/palate	Institute of Human Genetics, University of Tuebingen, Tuebingen, Germany	1 Subject, 2 Parents	-	Case study	Intellectual disability	Cleft lip and palate, bilateral talipes equinovarus, kyphoscoliosis, psychomotor development delay, short stature, bilateral convergent strabismus, dysmorphic facial features	Obstructive	Chromosome analysis	SNP array	-	9q34.11–q34.13 (3.7 Mb deletion)
Verbeek et al., 2012 [147]	COL4A2 mutation associated with familial porencephaly and small-vessel disease	Erasmus University Medical Center, Rotterdam, The Netherlands	10 Subjects, Controls used	Caucasian, Afghani	Case series	Porencephaly, periventricular leukoencephalopathy, cerebellar hypoplasia, cerebral atrophy	Developmental delay, feeding difficulties, bilateral ica, ophthalmological signs	Obstructive	TGS	SNP array	AD	13q34 (c.4165G4A, p.G1389R in exon 44 and c.3206delC in exon 34 in COL4A2)
Vieira et al., 2012 [148]	Primary ciliary dyskinesia and hydrocephalus with aqueductal stenosis	Hospital de Dona Estefânia, Centro Hospitalar de Lisboa Central, Lisbon, Portugal	1 Subject	Gypsy	Case study	Aqueductal stenosis	Dextrocardia, a complex heart malformation, situs inversus, intestinal malrotation	Obstructive	TES	-	AR	No mutations in DNAI1 or DNAH5

Amplification created restriction site (ACRS). Array comparative genomic hybridization (aCGH). Arthrogryposis multiplex congenital (AMC). Atrial septal defect (ASD). Autosomal dominant (AD). Autosomal recessive (AR). Copy number variant (CNV). Fluorescein isothiocyanate (FITC). Fluorescence-assisted mismatch analysis (FAMA). Fluorescence In Situ Hybridization (FISH). Heteroduplex analysis (HA). Internal carotid artery (ICA). Next generation sequencing (NGS). Restriction endonuclease fingerprinting (REF). Single nucleotide polymorphisms (SNP). Single nucleotide primer extension (SNuPE). Single-strand conformation polymorphisms (SSCP). Targeted exome sequencing (TES). Targeted genome sequencing (TGS). Whole exome sequencing (WES). Whole genome sequencing (WGS)

Understanding the function of these genes may confer a mechanistic and phenotypic understanding of HC secondary to AS. For example, some patients with AS will display abnormal brainstem development leading to near complete obliteration of the aqueduct, whereas other children may display relatively normal anatomy associated with a web obscuring CSF flow. Genetics factors contributing to AS include *ATP1A3*, which encodes an ATPase ion channel that has been associated with CNS development and ventricular dilatation when disrupted in zebrafish [34]. In addition, *SLC12A6* codes for the ion transporter KCC3 (K-Cl co transporter) that has been associated with AS among other phenotypes including peripheral neuropathy and agenesis of the corpus callosum in mice [35]. These ion channels are localized to the choroid plexus and are involved in neural stem cell development [36]. *ADAMTSL2*, encoding a glycoprotein, has been shown to interact with fibrillin 1 to enhance transforming growth factor- β (TGFβ) and fibroblast function. Additionally, TGFβ has been implicated in skeletal dysplasia and developmental dysfunction [37]. Thus, it is evident that genes with varying functions may contribute to AS and the diverse co-occurring phenotypes observed in these patients.

### X-linked hydrocephalus

Genes contributing to X-linked HC include apoptosis inducing factor mitochondria associated 1 (*AIFM1),* adaptor related protein complex 1 subunit sigma 2 *(AP1S2),* EBP cholestenol delta-isomerase *(EBP),* FA complementation group B *(FANCB),* histone deacetylase 6 *(HDAC6),* OFD1 centriole and centriolar satellite protein *(OFD1),* OTU deubiquitinase 5 *(OTUD5),* coiled-coil domain containing 22 (*CCDC22*), and porcupine O-acyltransferase *(PORCN).* Table 2 summarizes the genetic studies of X-linked HC in humans. *AIFM1* is involved in regulation of apoptosis [38]. In addition, *AP1S2* regulates endosomal protein trafficking and structural integrity [39]. HDAC6 has been shown to interact with Runx2, a transcription factor involved in osteoblast differentiation, and other HDACs exhibit high expression patterns in prehypertrophic chondrocytes, indicating their role in endochondral ossification and skeletal dysplasias [40]. OTUD5 mutations also impact transcriptional regulation with its inability to prevent HDAC degradation and maintain neural stem cell development [41]. OFD1 and PORCN mutations affect signaling pathways such as hedgehog signaling or wingless/integrated (Wnt) signaling [42, 43].

**Table 2:** X-linked hydrocephalus

Citation	Title	Author affiliation	Case #	Ancestry	Study design	CNS phenotype	Non-CNS phenotype	Type of hydrocephalus	Genetic methodology	Genetic analysis	Inheritance	Genetic findings
Alhousseini et al., 2019 [149]	Familial Hydrocephalus and Dysgenesis of the Corpus Callosum Associated with Xp22.33 Duplication and Stenosis of the Aqueduct of Sylvius with X-Linked Recessive Inheritance Pattern	Wayne State University, Detroit, Michigan, USA	2 Subjects, 1 Control	Furey	Case study	Global motor delay		Obstructive	Chromosomal microarray	CNV	X-linked Recessive	Xp22.33 (439 Kb duplication)
Beggs et al., 1992 [150]	Possible influences on the expression of X chromosome-linked dystrophin abnormalities by heterozygosity for autosomal recessive Fukuyama congenital muscular dystrophy	Howard Hughes Medical Institute, Children's Hospital, Boston, MA	37 Subjects, Controls used	Japanese	Case series	Comedullar atrophy, cortical dysgenesis	Congenital muscular dystrophy	Communicating	TGS	Southern blotting	AR with X linked interaction	Xp21.2-p21.1 (exons 51–54 deletion in DMD)
Berger et al., 2011 [38]	Early prenatal ventriculomegaly due to an AIFM1 mutation identified by linkage analysis and whole exome sequencing	Hadassah-Hebrew University Medical Center, Jerusalem, Israel	2 Subjects, 86 Controls	Palestinian	Case Study	Bilateral septated choroid plexus cysts, enlarged cisterna magna, swallowing difficulties, hypotonia	Hypertrophic cardiomyopathy, dystrophic muscle changes	Obstructive	WES	Linkage analysis	X-linked	Xq26.1 (c.G923A, p.G308E in exon 9 of AIFM1)
Cacciagli et al., 2013 [151]	AP1S2 is mutated in X-linked Dandy-Walker malformation with intellectual disability, basal ganglia disease and seizures (Pettigrew syndrome)	Faculté de Médecine de La Timone, Marseille, France	4 Subjects, Control matched sampling	-	Case series	Dandy walker malformation, intellectual disability, self-harm, ataxia, limb scissoring, spasticity, kyphoscoliosis	Facial dysmorphism with a long and narrow face, prominent mandible, inconstant choreoathetosis	-	WES	SNP analysis; Sanger Sequencing	X-linked	Xp22.2 (c.G426 + 1 T mutation in exon 4 of AP1S2)
Chassaing et al., 2005 [152]	X-linked dominant chondrodysplasia with platyspondyly, distinctive brachydactyly, hydrocephaly, and microphthalmia	Hôpital Pellegrin, CHU Bordeaux, France	4 Subjects	-	Case study	Macrocephaly	Microphthalmia, small low-set ears, and a short flat nose, platyspondyly, poor mineralization of the bones, 11 pairs of thin ribs, hypoplasia of the iliac wings, metaphyseal cupped phalanges, and hypoplastic bilobar-shaped calcaneus	-	TGS	Microsatellite marker assay; linkage analysis	X-linked dominant	X-linked dominant
Furtado et al., 2010 [153]	A novel X-linked multiple congenital anomaly syndrome associated with an EBP mutation	University of Utah Health Sciences Center, Salt Lake City, Utah, USA	1 Subject, 5 Controls	-	Case study	Dandy–walker malformation, dysgenesis of the corpus callosum,	Cataracts, bilateral cryptorchidism, collodian or ichthyotic skin, 2,3-toe syndactyly, robin anomaly, a high-nasal bridge, auricular dysplasia, and septal defects	Obstructive	TGS	Variant analysis, Sanger sequencing	X-linked recessive	Xp11.23 (c.G141T, p.W47C in exon 2 of EBP)
Holden et al., 2006 [154]	Fanconi anaemia complementation group B presenting as X linked VACTERL with hydrocephalus syndrome	Guy's Hospital, St Thomas Street, London SE1 9RT, UK	2 Subjects, 2 Controls	-	Case Study	Cervical vertebral defects, Arnold Chiari malformation, lumbar spina bifida occulta	Absent thumbs, unilateral renal agenesis, incomplete lung lobulation, cardiac defects, tracheoesophageal fistula/atresia, abnormal ear	Communicating	TGS	chromosome breakage assay, direct sequencing	X-linked	Xp22.2 (G to A substitution in intron 7 of FANCB which causes skipping of exon 7)
Jouet et al., 1995 [155]	New domains of neural cell-adhesion molecule L1 implicated in X-linked hydrocephalus and MASA syndrome	University of Cambridge Department of Medicine, Addenbrooke's Hospital, United Kingdom	9 Subjects	-	Case–Control	Intellectual disability, and spastic paraplegia type I	Aphasia, shuffling gait, adducted thumbs	-	TGS	Automated sequencing; SSCP; HA; direct radioactive cycle sequencing; SNuPE	X-linked	Xq28 (L1 gene mutations: c.G2302T, p.V768F; c.G361A, p.G121S; exon 1 (c.G26C, p.W9S); exon 8 (nucleotide G-to-A transition, p.E209K); exon 14 (c.C1756T, p.Q586X); exon 21 (c.C2822T, p.P941L); exon 24 (c.A3209G, p.Y1070C); base change at the intron 10 donor splice site resulting in the skipping of exon 10; point mutation in intron 26)
Kaepernick et al., 1994 [156]	Clinical aspects of the MASA syndrome in a large family, including expressing females	Michigan State University	22 Subjects	-	Case series	Intellectual disability, spasticity	Developmental delay, adducted thumbs, syndactyly of toes, rounded shoulders, shuffling gait, kyphosis, lordosis, hammer toes, metatarsus adductus, pes cavus, ankle	-	Southern blotting	DNA probing	X-linked	Mutation within Xq28
Kenwrick et al., 1986 [157]	Linkage studies of X-linked recessive spastic paraplegia using DNA probes	Nutfield Department of Clinical Medicine, John Radcliffe Hospital, OX39DU, Oxford, UK	6 Subjects	-	Case series	Spastic paraplegia, intellectual disability	Absence of extensor pollicis longus	-	Southern blotting	Linkage analysis	X-linked	Mutation within Xq28
Ko et al., 1994 [158]	Prenatal diagnosis of X-linked hydrocephalus in a Chinese family with four successive affected pregnancies	National Taiwan University Hospital, Taipei, Republic of China	4 Subjects	Chinese	Case study	Psychomotor delay, spastic quadriplegia, seizures	Aphasia	Obstructive	TES	Linkage analysis; direct sequencing	X-linked	Mutation within Xq28
Kolanczyk et al., 2015 [159]	Missense variant in CCDC22 causes X-linked recessive intellectual disability with features of Ritscher-Schinzel/3C syndrome	Institute for Medical Genetics and Human Genetics, Charité-Universitätsmedizin Berlin, Berlin, Germany	2 Subjects, 1 Parent, 1 Control	Australian	Case study	Dandy–Walker malformation, cerebellar vermis hypoplasia, posterior fossa cysts, and ventricular dilatation, intellectual disability	Facial dysmorphism, cardiac defects, glaucoma, VSD, cryptorchidism	Obstructive	WES, cytogenetics	NGS-based WES, aCGH, segregation analysis, Sanger sequencing	X-linked recessive	Xp11.23 (c.A1670G, p.Y557C in exon 15 of CCDC22)
Kroes et al., 2005 [160]	Cerebral, cerebellar, and colobomatous anomalies in three related males: Sex-linked inheritance in a newly recognized syndrome with features overlapping with Joubert syndrome	University Medical Center Utrecht, Utrecht, The Netherlands	3 Subjects, 1 Control	Caucasian, Indonesian	Case study	Neural tube defect, convulsions, hypotonia, Dandy walker—cerebellar malformations, hypotonia, molar tooth sign,	Meckel’s diverticulum, Facial dysmorphism, bilateral colobomas	Communicating/Obstructive	TGS	X-inactivation status	X-linked recessive	X-linked inheritance
Legius et al., 1994 [161]	Fine mapping of X-linked clasped thumb and mental retardation (MASA syndrome) in Xq28	University of Michigan, Department of Pediatric Genetics, Ann Arbor	49 Subjects	-	Case Study	Spastic paresis, Intellectual disability	Adducted thumbs, global physical delay	-	Haplotyping	Two-point and Multipoint linkage analysis	X-linked	Xq28 (Genetic etiology of MASA syndrome is localized to between DXS455 and F8C)
McCauley et al., 2011 [162]	X-linked VACTERL with hydrocephalus syndrome: further delineation of the phenotype caused by FANCB mutations	GSTS Pathology, Guy's Hospital, London, UK	10 Subjects, Controls used	-	Case series	Isolated hydrocephalus	Vertebral defects, hear anomalies, esophageal/duodenal/anal atresia, renal abnormalities, genital abnormalities (vacterl-h like phenotype)	-	TGS	Direct sequencing	X-linked, De novo	Xp22.2 (FANCB mutations: deletion of exons 8–10; c.2165 + 1G > T exon 9 donor splice site mutation; c.1857_1858delAG, p.R619fs; c.T2150G, p.L717X)
Mikat et al., 2016 [163]	X-linked recessive VACTERL-H due to a mutation in FANCB in a preterm boy	University Hospital Essen, University Duisburg-Essen, Duisburg and Essen	1 Subject, 1 Control	Caucasian	Case study	Isolated hydrocephalus	Bilateral renal agenesis, posteriorly rotated ears, retrognathia, oligodactyly of the hands, bilateral dysplasia of the radius and ulna, anal atresia, and myocardial hypertrophy	-	TGS	-	X-linked recessive	Xp22.2 (c.C832T, p.Q278X in FANCB)
Peters et al., 2014 [42]	Focal dermal hypoplasia: report of a case with myelomeningocele, Arnold-Chiari malformation and hydrocephalus with a review of neurologic manifestations of Goltz syndrome	University of Calgary, Calgary, Alberta, Canada	1 Subject	Nigerian	Case study	Arnold Chiari I malformation, lumbosacral meningomyelocele	Cryptorchidism, pointed chin and low set under folded ears with hypopigmentation of the helices linear hypoplasia and atrophy of the skin, bilateral iris and chorioretinal colobomas, syndactyly	Obstructive	TGS	a-CGH	X-linked, De novo	Xp11.23 (c853_855delACG in PORCN)
Rietschel et al., 1991 [164]	MASA syndrome: clinical variability and linkage analysis	Institut für Humangenetik der Universität Bonn, Germany	3 Subjects	-	Case series	Intellectual disability, spastic paraplegia	Aphasia, shuffling gait, and adducted thumbs	-	Chromosome analysis	Linkage analysis	X-linked	Mutation within Xq28
Rosenthal, Jouet, Kenwrick, 1992 [165]	Aberrant splicing of neural cell adhesion molecule L1 mRNA in a family with X-linked hydrocephalus	University of Cambridge, Addenbrooke's Hospital, UK	2 Subjects, 2 Controls	-	Case–Control	Intellectual disability, spasticity	Adducted thumbs	-	TGS	Direct sequencing	X-linked	Xq28 (intronic A to C base change 19 bp upstream of a splice acceptor site in the L1 gene)
Saillour et al., 2007 [39]	Mutations in the AP1S2 gene encoding the sigma 2 subunit of the adaptor protein 1 complex are associated with syndromic X-linked mental retardation with hydrocephalus and calcifications in basal ganglia	Institut Cochin, Université Paris Descartes, CNRS (UMR 8104), Paris, France	8 Subjects	Scottish, French	Case series	Hypotonia, calcification of the basal ganglia, intellectual disability, seizures	Osteosclerosis of the calvarium, mild facial dysmorphism	Communicating/Obstructive	WGS	Microsatellite marker assay; linkage analysis, chromatographic mutation analysis	X-linked	Xp22.2 (c.288 + 5G > A in AP1S2)
Schrander-Stumpel et al., 1990 [166]	MASA syndrome: new clinical features and linkage analysis using DNA probes	State University of Limburg, The Netherlands	3 Subjects	-	Case series	Intellectual disability, macrocephaly, spastic paraplegia	Aphasia, shuffling gait, adducted thumbs, divergent strabismus, myopia, astigmatism, anteverted hip and shoulders, bowed knees, pupils irregularly shaped and not reactive to light, camptodactyly of fingers, dysarthria	-	Southern blotting	Linkage analysis	X-linked recessive	Mutation within Xq28
Serville et al., 1992 [167]	X-linked hydrocephalus: clinical heterogeneity at a single gene locus	CHU, Hôpital Pellegrin, Bordeaux, France	3 Subjects	-	Case series	Cortex thinning, spasticity, cerebral palsy, intellectual disability, corpus callosum agenesis, aqueductal stenosis	Bilateral adducted thumbs	-	Southern blotting	Linkage analysis	X-linked	Mutation within Xq28
Sheen et al., 2004 [168]	Etiological heterogeneity of familial periventricular heterotopia and hydrocephalus	Beth Israel Deaconess Medical Center, Harvard Medical School, HIM 816, 4 Blackfan Circle, Boston, MA 02115, USA	3 Subjects	Caucasian (Australian & American), Ethiopian	Case series	Periventricular heterotopia, callosal agenesis, hypotonia, Chiari I malformation and aqueductal stenosis, seizures	Pulmonary artery stenosis, cardiac defects, bilateral per planovalgus, bilateral knee recurvatum, bilateral hip dysplasia, dysmorphic facial features	Obstructive	TGS	Linkage analysis	X-linked, Autosomal	Xq28
Simon et al., 2010 [169]	A mutation in the 3'-UTR of the HDAC6 gene abolishing the post-transcriptional regulation mediated by hsa-miR-433 is linked to a new form of dominant X-linked chondrodysplasia	Laboratoire de Génétique Humaine, EA 4137, Université Victor Segalen Bordeaux 2, Bordeaux 33,076, France	2 Subjects, 1 Control	-	Case series	Isolated hydrocephalus	Platyspondyly, rhizomelic shortening of the members, specific brachydactyly, hydrocephaly, facial dysmorphism and microphthalmia	-	TES, cytogenetics	Linkage analysis; aCGH	X-linked dominant	Xp11.23 (c.*281A > T in exon 29 of HDAC6 gene)
Tripolszki et al., 2020 [170]	An X-linked syndrome with severe neurodevelopmental delay, hydrocephalus, and early lethality caused by a missense variation in the OTUD5 gene	CENTOGENE GmbH, Rostock, Germany	13 Subjects, Controls used	Irish Caucasian	Case study	Severe neurodevelopmental delay, hypotonia	Growth retardation, congenital heart defects, hypospadias	Obstructive	WGS	Variant analysis	X-linked	Xp11.23 (c.G598A, p.E200K in exon 2 of OTUD5)
Watanabe et al., 2018 [171]	X-linked VACTERL-H caused by deletion of exon 3 in FANCB: A case report	Yamagata University Faculty of Medicine, Yamagata, Japan	1 Subject	-	Case study	Isolated hydrocephalus	Tetralogy of fallot, absence of pulmonary valve, tracheoesophageal fistula, esophageal atresia, bilateral radial aplasia, left renal dysplasia, duodenal atresia, imperforate anus, and cleft vertebrae	-	WES	CNV, MLPA analysis	X-linked	Xp22.2 (exon 3 deletion in FANCB)
Willems et al., 1990 [172]	Assignment of X-linked hydrocephalus to Xq28 by linkage analysis	University of Antwerp-UIA, Wilrijk, Belgium	7 Subjects, 34 Family Members, 55 Controls	Dutch, UK, USA	Case Series	Stenosis of the aqueduct of Sylvius, intellectual disability, spastic paraparesis	Clasped thumbs	-	X chromosome DNA marker probing	Southern Blot analysis, Linkage analysis	X-linked	mutation located on Xq28
Willems et al., 1992 [173]	Further localization of X-linked hydrocephalus in the chromosomal region Xq28	University of Antwerp-UIA, Belgium	20 Subjects, 84 Family Members	Netherlands, European, Israeli, German, French	Case series	Stenosis of the aqueduct of Sylvius, intellectual disability, and spastic paraparesis	Clasped thumbs	-	Southern blotting	Two-point and multipoint linkage analysis	X-linked	Gene mutations within Xq28 (between DXS52 and F8C)
Zhang et al., 2021 [43]	A rare mutant of OFD1 gene responsible for Joubert syndrome with significant phenotype variation	West China Hospital, Sichuan University and Collaborative Innovation Center, Chengdu, 610,041, China	1 Subject, 5 Family Members, 201 Controls	-	Case study	Agenesis of cerebellar vermis and abnormal brain stem	Tetralogy of fallot	Obstructive	WES	Sanger sequencing	X-linked recessive	Xp22.2 (c.599 T > C, p.L200P in exon 8 of OFD1)

Amplification created restriction site (ACRS). Array comparative genomic hybridization (aCGH). Atrial Septal Defect (ASD). Autosomal Recessive (AR). Central Nervous System (CNS), Copy number variant (CNV). Deep tendon reflexes (DTR). Denaturing gradient gel electrophoresis (DGGE). Fluorescein isothiocyanate (FITC). Fluorescence assisted mismatch analysis (FAMA). Mental retardation, aphasia, shuffling gait, and adducted thumbs syndrome (MASA syndrome). Multiplex ligation dependent probe amplification (MLPA). Restriction endonuclease fingerprinting (REF). Targeted exome sequencing (TES). Targeted genome sequencing (TGS). Ventricular septal defect (VSD). Whole exome sequencing (WES). Whole genome sequencing (WGS). Internal carotid artery (ICA)

### L1CAM associated hydrocephalus

**Fig. 3:**
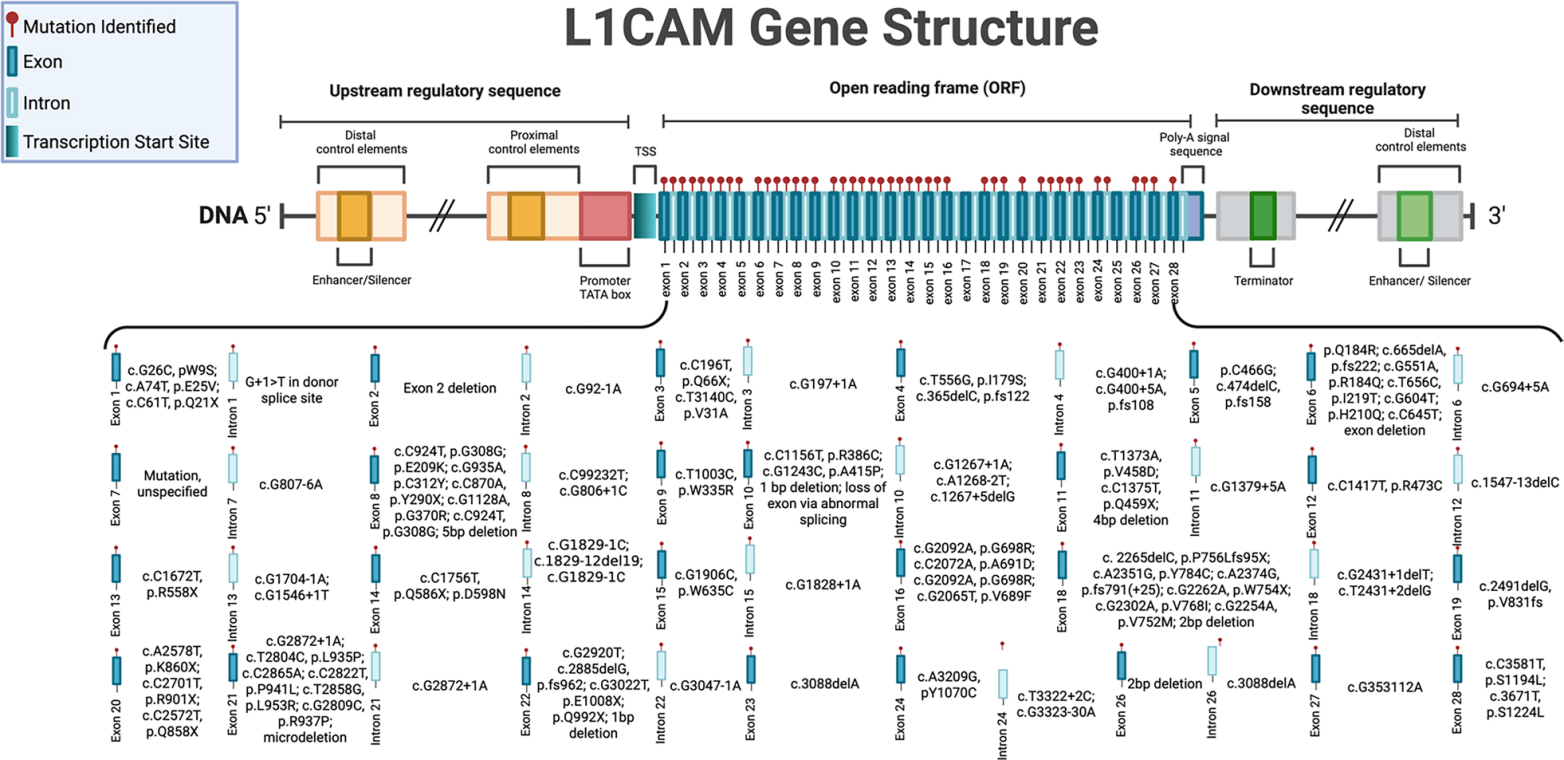
L1CAM mutations implicated in human patients with hydrocephalus

Next, we discuss L1CAM associated HC, as this entity is well described and distinct phenotypically. Early linkage analysis studies of HC identified a mutation within the long arm of chromosome X, specifically Xq28. Further genomic analyses localized to a region between the gene loci of *DXS52* and *F8C*, within which L1 cell adhesion molecule (*L1CAM)* resides. The genetic understanding of X-linked HC has primarily been linked to genetic alterations at the *L1CAM* locus. *L1CAM* duplications include the 3’ end of the open reading frame and exons 2–10. *L1CAM* insertions include exon 18 and the junction sequence between *L1CAM* and *AVPR2*. *L1CAM* deletions/microdeletions include exons 2, 5–8, 10, 11, 18, 19, 21–23, 26, intron 18, and whole gene deletion. *L1CAM* missense mutations include exons 1–16, 18, 20, 21, 24, 27, 28 and introns 2–4, 6–8, 10–15, 18, 21, 22, 24, and 26. *L1CA*M nonsense mutations include exons 1, 3, 8, 10–14, and 20–22. A silent mutation in Exon 8 of *L1CAM* has been associated with HC. A summary of all mutations across L1CAM can be found in Fig. 3 and Table 3. Mutations in L1CAM are also associated with MASA syndrome (characterized by mental retardation, aphasia, shuffling gait, and adducted thumb), and spastic paraplegia, highlighting the pleiotropic role of L1CAM in human disease.

**Table 3:** L1CAM

Citation	Title	Author affiliation	Case #	Ancestry	Study design	CNS phenotype	Non-CNS phenotype	Type of hydrocephalus	Genetic methodology	Genetic analysis	Inheritance	Genetic findings
Bott et al., 2004 [174]	Congenital idiopathic intestinal pseudo-obstruction and hydrocephalus with stenosis of the aqueduct of sylvius	Jeanne de Flandre Hospital, Faculty of Medicine Lille, France	1 Subject	French	Case study	Bilateral nystagmus, convergent strabismus, spastic paraplegia, callosal agenesis	Bilateral adducted thumbs, abdominal distension	Obstructive	-	-	X-linked recessive	Xq28 (G2920T in exon 22 of L1CAM)
Brewer et al., 1996 [175]	X-linked hydrocephalus masquerading as spina bifida and destructive porencephaly in successive generations in one family	Western General Hospital, Edinburgh, UK	1 Subject	-	Case study	Midline cysts, callosal agenesis, cognitive impairment	Asymmetric tetraplegia, low vision, eye movement disorder	Obstructive	-	-	X-linked recessive	Xq28 (Frameshift mutation in L1CAM)
Chidsey et al., 2014 [176]	L1CAM whole gene deletion in a child with L1 syndrome	ARUP Laboratories, Salt Lake City, Utah	1 Subject, 1 Parent	North European	Case study	Absent septum pellucidum	Adducted thumbs with contractures	Obstructive	microarray analysis	SNP analysis	X-linked	Xq28 (62 kb deletion)
Claes et al., 1998 [177]	Hydrocephalus and spastic paraplegia result from a donor splice site mutation (2872 + 1G to A) in the L1CAM gene in a Venezuelan pedigree	Center for Human Genetics, University of Leuven, Belgium	3 Subjects	Venezuela	Case study	Aqueductal stenosis, psychomotor delay, hypotonia, spastic paraplegia	Learning difficulties	Obstructive	cDNA analysis; TGS	Solid-phase approach w/ FITC primer	X-linked	Xq28 (exon 21 microdeletion in L1CAM; G-to-A transition at bp 2872 + 1 of exon 21)
Coucke et al., 1994 [178]	Identification of a 5' splice site mutation in intron 4 of the L1CAM gene in an X-linked hydrocephalus family	University of Antwerp-UIA, Belgium	1 Subject, 1 Control	-	Case study	Aqueductal stenosis, intellectual disability, spastic paresis	Clasped thumbs	-	RT-PCR	Linkage analysis	X-linked	Xq28 (G to A transition at position –5 of the 5’ splice site of intron 4 of L1CAM)
Du et al., 1998 [179]	A silent mutation, C924T (G308G), in the Li CAM gene results in X linked hydrocephalus (HSAS)	J C Self Research Institute of Human Genetics, Greenwood Genetic Center, SC 29646, USA	1 Subject, 1 Family member	-	Case study	Callosal agenesis, intellectual disability, spastic paraplegia	Clenched fingers, overlapping digits	Obstructive	TES	REF	X-linked	Xq28 (c.C924T, p.G308G silent mutation in exon 8 of L1CAM)
Du et al., 1998 [180]	Multiple exon screening using restriction endonuclease fingerprinting (REF): detection of six novel mutations in the L1 cell adhesion molecule (L1CAM) gene	J C Self Research Institute of Human Genetics, Greenwood Genetic Center, SC 29646, USA	5 Subjects, 200 Controls	-	Case series	Intellectual disability	Adducted thumbs with contractures, aphasia	-	TGS	SSCP, REF	X-linked, De novo	Xq28 (6 novel mutations in the L1CAM gene: intron 7 (G to A base substitution at position 807–6 at the 3 ´ splice site); intron 11 (G to A transition at position 1379 + 5 within the 5 ´ splice site); intron 10 (A to T base change at position 1268–2 of the 3 ´ splice site); within exons 16–18 (c.G2092A, p.G698R); exon 16 (c.C2072A, p.A691D); exon 21 (c.T2804C, p.L935P))
Du et al., 1998 [181]	Somatic and germ line mosaicism and mutation origin for a mutation in the L1 gene in a family with X-linked hydrocephalus	J C Self Research Institute of Human Genetics, Greenwood Genetic Center, SC 29646, USA	5 Subjects, 200 Controls	-	Case study	Intellectual disability	Adducted thumbs with contractures, aphasia	Obstructive	TGS	SSCP	X-linked	Xq28 (G to A nucleotide change at the first position of intron 10 of L1CAM)
Ferese et al., 2016 [182]	A New Splicing Mutation in the L1CAM Gene Responsible for X-Linked Hydrocephalus (HSAS)	Localita' Camerelle, Pozzilli, Italy	1 Subject, 1 Control	-	Case study	Aqueductal stenosis, thinned cerebral parenchyma, lissencephaly, corpus callosum agenesis,	Adducted thumbs, dysmorphic facial features	Obstructive	TGS	Direct sequencing	X-linked recessive	Xq28 (Intron 10 in L1CAM hemizygous for c.1267 + 5delG; loss of exon 10 via abnormal splicing)
Fernandez et al., 2012 ([183]	Association of X-linked hydrocephalus and Hirschsprung disease: report of a new patient with a mutation in the L1CAM gene	Instituto de Biomedicina de Sevilla (IBIS), Hospital Universitario Virgen del Rocío/CSIC/Universidad de Sevilla, Sevilla, Spain	1 Subject	-	Case study	Bilateral spastic tetraparesis, psychomotor growth delay	Cephalo-pelvic dystocia, hirschsprung’s disease	Obstructive	TES	-	X-linked	Xq28 (c.2092G > A, p.G698R in exon 16 of L1CAM)
Finckh et al., 2000 [184]	Spectrum and Detection Rate of L1CAM Mutations in Isolated and Familial Cases with Clinically Suspected L1-Disease	University Hospital Eppendorf, University of Hamburg, Hamburg, Germany	153 Subjects, 100 controls	-	Case series	Spastic paraplegia, intellectual disability, agenesis, hypoplasia of corpus callosum	Adducted thumbs with contractures, cleft palate, heart malformation, esophageal atresia, club feet	-	TES	SSCP	X-linked, De novo	Xq28 (L1CAM mutations)
Fransen et al., 1994 [185]	X-linked hydrocephalus and MASA syndrome present in one family are due to a single missense mutation in exon 28 of the L1CAM gene	University of Antwerp-UIA, Belgium	1 Subject	-	Case study	Extreme macrocephaly, severe spasticity, and intellectual disability	Shuffling gait, adducted thumbs	-	Southern Blotting	SSCP analysis	X-linked	Xq28 (c.C3581T, p. S1194L in exon 28 of L1CAM)
Gigarel et al., 2004 [186]	Single cell co-amplification of polymorphic markers for the indirect preimplantation genetic diagnosis of hemophilia A, X-linked adrenoleukodystrophy, X-linked hydrocephalus and incontinentia pigmenti loci on Xq28	Hôpital Necker Enfants Malades, 75,015 Paris, France	10 Subjects, Controls used	-	Case Series	Isolated hydrocephalus	Hemophilia a	-	TGS	Microsatellite marker assay	X-linked recessive	Xq28 (2872 + 1 G to A mutation in intron 21 of L1CAM)
Graf et al., 2000 [187]	Diffusion-weighted magnetic resonance imaging in boys with neural cell adhesion molecule L1 mutations and congenital hydrocephalus	University of Washington School of Medicine, Seattle, USA	5 Subjects, 1 Control	Italian	Case series	Agenesis of the corpus callosum, diffuse cerebral dysplasia, decreased white matter, small posterior fossa, Chiari I malformation	Developmental delay	Obstructive	TES	REF	X-linked	Xq28 (14 bp deletion in exon 11, 1 bp deletion in exon 10, p.C466G in exon 5, and p.R184W in L1CAM)
Gregory et al., 2019 [188]	Mutations in MAGEL2 and L1CAM Are Associated with Congenital Hypopituitarism and Arthrogryposis	UCL Great Ormond Street Institute of Child Health, London, United Kingdom	5 subjects, Controls used	European, Chile, Afro-Caribbean	Case series	Hypotonia, bulky tectum, white matter loss, thin corpus callosum	Bilateral radial clubbed hands, plagiocephaly, distal arthrogryposis with adducted thumbs, flexion deformities, growth hormone deficiency, ventricular septal defect, severe obstructive sleep apnea, global developmental delay, right hip subluxation, scoliosis, bilateral astigmatism, visual impairment	Communicating	WES; chromosome microarray	Sangar Sequencing; Human embryonic expression analysis; Ingenuity Variant analysis	X-linked	Xq28 (c.G1354A, p.G452R in L1CAM)
Griseri et al., 2009 [189]	Complex pathogenesis of Hirschsprung's disease in a patient with hydrocephalus, vesico-ureteral reflux and a balanced translocation t(3;17)(p12;q11)	Laboratory Molecular Genetics and Cytogenetics, Genova, Italy	1 Subject, 1 Control	-	Case Study	Intellectual disability, bilateral spastic paraplegia	Adducted thumbs, vesico-ureteral reflux, developmental delay	-	TGS	-	X-linked, De novo	Xq28 (c.2265delC, p.P756Lfs95X in exon 18 of L1CAM); haploinsufficiency of MYO18A/TIAF1 genes involved in a balanced translocation (3;17)(p12;q21)
Gu et al., 1996 [190]	Five novel mutations in the L1CAM gene in families with X linked hydrocephalus	Institut fur Humangenetik, Medizinische Universitat zu Lubeck, Germany	5 Subjects	-	Case series	Intellectual disability, spastic paresis, complex brain malformation with agenesis of the corpus callosum and fusion of the Thalamus	Deafness, Adducted thumbs, global physical delay, cleft lip and palate	-	TES	SSCP, HA	X-linked, De novo	Xq28 (mutations in exon 1, 6, 7, and 8 of L1CAM)
Guo et al., 2020 [191]	A novel nonsense mutation in the L1CAM gene responsible for X-linked congenital hydrocephalus	Xiangya Hospital, Central South University, Changsha, Hunan, China	1 Subject	Chinese	Case study	Agenesis of the corpus callosum, vermis hypoplasia and enlargement of the quadrigeminal plate, aqueductal stenosis	Tower-shaped skull, contractions of both thumbs	Obstructive	WES; chromosomal karyotyping; chromosomal microarray analysis	Sangar Sequencing; Variant segregation analysis	X-linked recessive	Xq28 (c.C2865A in exon 21 of L1CAM)
Hubner et al., 2004 [192]	Intronic mutations in the L1CAM gene may cause X-linked hydrocephalus by aberrant splicing	University Hospital Eppendorf, Hamburg, Germany	7 Subjects, 50 Controls	-	Case series	Intellectual disability, hypoplastic or absent corticospinal tract, callosal agenesis, spastic paraparesis, aqueductal stenosis	Aphasia, shuffling gait, adducted thumbs	-	TGS	SSCP analysis	X-linked	Xq28 (Intronic L1CAM sequence variants: c.523 + 5G > A; c.1123 + 1G > A; c.1547-13delC in intron 12; c.3323-17dupG; c.3457 + 3A > T; c.3457 + 18C > T; and c.523 + 12C > T)
Jouet et al., 1994 (79)	X-linked spastic paraplegia (SPG1), MASA syndrome and X-linked hydrocephalus result from mutations in the L1 gene	University of Cambridge, Addenbrooke's Hospital, UK	6 Subjects	-	Case series	Agenesis of the corpus callosum; agenesis of the septum pellucidum; fusion of thalami and hypoplasia of the corticospinal tract, aqueductal stenosis, spastic diplegia	Adducted thumbs	Obstructive	TES	SSCP, HA	X-linked	Xq28 (L1 gene mutations: 2 bp deletion in exon 26; single nucleotide deletion in exon 22; p.H210Q in second Ig domain; G to A nucleotide change, p.Q184R in exon 6; C to T mutation in exon 12 that introduces a stop codon at amino acid position 485; G to A mutation in exon 11 that changes a Gly to Arg residue)
Jouet et al., 1995 [193]	Gene analysis of L1 neural cell adhesion molecule in prenatal diagnosis of hydrocephalus	Addenbrooke's Hospital, Cambridge, UK	2 Subjects	-	Case series	Intellectual disability, spastic paraparesis	Developmental delay, adducted thumbs	-	TGS	SSCP, Direct sequencing	X-linked, De novo	Xq28 (g + 1- > t in the intron 1 donor splice site and 1 bp deletion in exon 22 of L1CAM)
Jouet et al., 1996 [194]	Discordant segregation of Xq28 markers and a mutation in the L1 gene in a family with X linked hydrocephalus	University of Cambridge, UK	19 Subjects	-	Case study	Intellectual disability, and spastic paraplegia type I	Aphasia, shuffling gait, adducted thumbs	-	TES	SSCP; HA	X-linked	Xq28 (deletion of a single adenosine at position 3088 in exon 23 of L1CAM)
Kanemura et al., 2006 [195]	Molecular mechanisms and neuroimaging criteria for severe L1 syndrome with X-linked hydrocephalus	Osaka National Hospital, Osaka, Japan	96 Subjects, 7 Controls	Japanese	Case series	Corpus callosum agenesis, vermis hypoplasia, epilepsy, spastic paraplegia	Bilateral adducted thumbs, developmental delay, elevated diaphragm	-	TGS	Direct sequencing	X-linked, De novo	Xq28 (L1CAM mutations: exon 1 (c.A74T, p.E25V); exon 5 (c.474delC, p.fs158); exon 6 (c.665delA, p.fs222); exon 8 (c.G935A, p.C312Y; c.C870A, p.Y290X); exon 11 (c.T1373A, p.V458D); exon 16 (c.G2065T, p.V689F); exon 18 (c.G2254A, p.V752M); exon 20 (c.A2578T, p.K860X; c.C2701T, p.R901X); exon 21 (c.T2858G, p.L953R); exon 22 (c.2885delG, p.fs962; c.G3022T, p.E1008X); intron 2 (c.92-1gA); intron 3 (c.197 + 1gA); intron 4 (400 + 1gA); intron 6 (c.694 + 5gA); intron 13 (c.1704-1gA); intron 14 (c.1829-1gC; c.1829-12del19bp); intron 15 (c.1940–21 ~ 1940–6); intron 18 (c.2431 + 1delGT); intron 21 (c.2872 + 1gA); intron 22 (c.3047-1gA))
Kong et al., 2019 [196]	A new frameshift mutation in L1CAM producing X-linked hydrocephalus	Sichuan Provincial Hospital for Women and Children, Chengdu, China	1 Subject, 2 Parents	-	Case study	Callosal agenesis and lissencephaly	-	-	WES	Sangar Sequencing	X-linked recessive	Xq28 (c.2491delG (p.V831fs) in exon 19 of L1CAM)
Liebau et al., 2007 [197]	L1CAM mutation in a boy with hydrocephalus and duplex kidneys	University Hospital of Freiburg, Mathildenstrasse 1, Freiburg, Germany	1 Subject	-	Case study	Tower-shaped skull, corpus callosum agenesis, Intellectual disability, microcephaly, strabismus, neurogenic bladder dysfunction, spasticity	Bilateral duplex kidneys and ureters, unilateral mega-ureter, adducted thumbs	-	TGS	SSCP	X-linked	Xq28 (c.2431 + 2delTG at the beginning of intron 18 of L1CAM)
Limbrick et al., 2017 [198]	Cerebrospinal fluid biomarkers of infantile congenital hydrocephalus	Washington University in St. Louis, School of Medicine, Saint Louis, MO, United States of America	20 Subjects, 51 Controls	Caucasian, Black, Asian	Case series	Isolated hydrocephalus		Obstructive	Chromosomal microarrays, TGS	-	-	Xq28 (G847X mutation in L1CAM); 1q25.2 anomaly; 11q24.2 anomaly
MacFarlane et al., 1997 [199]	Nine novel L1 CAM mutations in families with X linked hydrocephalus	University of Cambridge Department of Medicine, Addenbrooke's Hospital, Cambridge, UK	20 Subjects, 56 Controls	-	Case series	Intellectual disability, spastic paraplegia, corpus callosum agenesis, absence of the cortical spinal tract	Adducted thumbs	Obstructive	TES	SSCP; SNuPE	X-linked	Xq28 (L1CAM: exon 6 (c.G551A, p.R184Q); exon 11 (microdeletion); exon 13 (c.C1672T, p.R558X); exon 18 (c.A2351G, p.Y784C; c.A2374GG, p.fs791(+ 25); c.G2262A, p.W754X); exon 20 (c.C2701T, p.R901X); exon 21 (microdeletion); intron 7 (c.G(807–6)A); intron 24 (c.T(3322 + 2)C))
Marin et al., 2015 [200]	Three cases with L1 syndrome and two novel mutations in the L1CAM gene	Hospital Universitario Puerta del Mar, Cádiz, Spain	3 subjects	-	Case series	Corpus callosum agenesis, microcephaly, spastic paraplegia,	Developmental delay, bilaterally flexed adducted thumbs, bilateral clinodactyly of the fifth finger	-	TES	Sangar Sequencing	X-Linked recessive, De novo	Xq28 (L1CAM mutations: c.A1754C, p.D585A; c.C3478T, p.Q1160X; c.G353112A in exon 27)
Marx et al., 2012 [201]	Pathomechanistic characterization of two exonic L1CAM variants located in trans in an obligate carrier of X-linked hydrocephalus	Institute of Anatomy and Cell Biology, Center for Neurosciences, University of Freiburg, Freiburg, Germany	3 Subjects, Control cells used	-	Case study	Aqueductal stenosis	Adducted thumbs	Obstructive	TGS	Direct sequencing	X-linked	Xq28 (L1CAM mutations: c.C99232T in intron 8; c.G1906C, p.W635C in exon 15; c.G2302A, p.V768I in exon 18)
Michaelis et al., 1998 [202]	The site of a missense mutation in the extracellular Ig or FN domains of L1CAM influences infant mortality and the severity of X linked hydrocephalus	Center for Molecular Studies, J C Self Research Institute, Greenwood Genetic Center, SC 29646, USA	7 Subjects, Controls used	-	Case series	Intellectual disability, spasticity, aqueductal stenosis	Adducted thumbs	Obstructive	TES	SSCP, REF	X-linked	Xq28 (missense mutations in the extracellular Ig or FN domains of L1CAM)
Nakakimura et al., 2008 [203]	Hirschsprung's disease, acrocallosal syndrome, and congenital hydrocephalus: report of 2 patients and literature review	Hokkaido University Graduate School of Medicine, Sapporo, Japan	2 Subjects	-	Case series	Callosal body agenesis, spastic paralysis, and porencephaly	Bilateral inferior limbs, and bilateral thumb adduction, polydactyly	Obstructive	TGS	Direct sequencing	X-linked	Xq28 (c.T3140C, p.V31A in exon 3 of L1CAM)
Okamoto et al., 1997 [204]	Hydrocephalus and Hirschsprung's disease in a patient with a mutation of L1CAM	Osaka Medical Centre, Japan	1 Subject	Japanese	Case study	Intellectual disability, spastic quadriplegia, agenesis of the corpus callosum and septum Pellucidum, irregular ventricular wall, hypoplastic white matter, cerebellar hypoplasia, and Fusion of the thalami	Cleft palate, micrognathia, abdominal distension, bilateral adducted thumbs, and flexion contractures of the fingers	-	TES	Fluorescent dideoxy terminator method	X-linked, De novo	Xq28 (2 bp deletion in exon 18 of L1CAM)
Okamoto et al., 2004 [205]	Hydrocephalus and Hirschsprung’s disease with a mutation of L1CAM	Osaka Medical Center and Research Institute for Maternal and Child Health, 840 Murodo-cho, Izumi, Osaka, Japan	3 Subjects	Canadian, Spanish	Case series	Cerebellar hypoplasia, corpus callosal dysgenesis, thalami fusion, decreased white matter, aqueductal stenosis, intellectual disability, spastic paraparesis	Hirschsprung’s disease, bilateral adducted thumbs, flexion contracture of fingers, aphasia	Obstructive	TGS	Direct sequencing	X-linked recessive	Xq28 (Intron 15 mutation and p.Q992X in exon 22 of L1CAM)
Panayi et al., 2005 [206]	Prenatal diagnosis in a family with X-linked hydrocephalus	National Taiwan University Hospital, Taipei, Republic of China	1 Subject	-	Case study	Aqueductal stenosis, Underdevelopment of brain tissue, spastic quadriplegia, seizures, and psychomotor retardation	Aphasia	Obstructive	TES	Cycle sequencing, SSCP, HA	X-linked, De novo	Xq28 (deletion of exon 2 and 6 in L1CAM)
Parisi et al., 2002 [207]	Hydrocephalus and intestinal aganglionosis: is L1CAM a modifier gene in Hirschsprung disease?	University of Washington and Children's Hospital and Regional Medical Center, Seattle, Washington 98,105, USA	1 Subject, 1 Control	-	Case study	Macrocephaly, aqueductal stenosis, corpus callosum agenesis	Bilateral adducted thumbs and index fingers, bilateral inguinal hernias, hirschsprung's disease, developmental delay, micropenis, small descended right testis, cryptorchid left testis, upgoing toes, limb spasticity, strabismus, amblyopia	Obstructive	TGS	REF, SSCP	X-linked	Xq28 (c.G2254A, p. V752M in exon 18 of L1CAM)
Pomili et al., 2000 [208]	MASA syndrome: ultrasonographic evidence in a male fetus	University Hospital, Perugia, Italy	1 Subject	Italian	Case study	Intellectual disability, spasticity of the lower limbs, callosal hypoplasia	Colorblindness, bilaterally adduced thumbs	-	TGS	DGGE; direct sequencing	X-linked	Xq28 (G > A base substitution 12 bp upstream from the intron/exon boundary of exon 27 in L1CAM gene)
Rehnberg et al., 2010 [209]	Novel L1CAMSplice Site Mutation in a Young Male with L1 Syndrome	Linköping University, University Hospital, Linköping, Sweden	1 Subject, 3 Family Members	Swedish	Case study	Global hypotonia, intellectual disability, spastic paraplegia	Bilateral adducted thumbs	-	TGS	Dideoxynucleotide sequencing	X-linked, De novo	Xq28 (c.G3458-1C in L1CAM)
Rodríguez Criado et al., 2003 [210]	X-linked hydrocephalus: another two families with an L1 mutation	Unidad de Dismorfología, H.I.U.V. Rocío, Sevilla, Spain	3 Subjects	-	Case series	Intellectual disability	Aphasia, shuffling gait, and adducted thumbs	Obstructive	TGS	DGGE; REF; direct sequencing	X-linked, De novo	Xq28 (c.C196T, p.Q66X in exon 3 of L1CAM; 1267 + 1G > A in intron 10 of L1CAM)
Ruiz et al., 1995 [211]	Mutations in L1-CAM in two families with X linked complicated spastic paraplegia, MASA syndrome, and HSAS	University of Leuven, Belgium	3 Subjects	-	Case–Control	Spastic paresis, intellectual disability, aqueductal stenosis	Adducted thumbs	Obstructive	TGS	Solid-phase approach w/ FITC primer; Dot Blot Assay	X-linked	Xq28 (15 bp deletion was found at coding position 97 of the cDNA; 12 bp deletion at bp3551; c.T875C; insertion of a cytosine at nucleotide position 3806 within the 3' untranslated region; exons 4,5,6 (c.T556G, p. I179S); exons 8,9,10 (c.G1128A, p.G370R))
Saugier-Veber et al., 1998 [212]	Identification of novel *L1CAM* mutations using fluorescence-assisted mismatch analysis	Laboratoire de Génétique Moléculaire, CHU de Rouen, France	13 Subjects, 100 Controls	French	Case series	Intellectual disability, spastic paraplegia	Aphasia, shuffling gait, adducted thumbs, hirschsprung’s disease	Obstructive	TGS; genotyping	FAMA; ACRS	X-linked, De novo	Xq28 (L1CAM nucleotide changes: c.365delC, p.FS122 in exon 4; c.400 + 5G > A, p.FS108 in intron 4; c.T656C, p. I219T in exon 6; c.T1003C, p. W335R in exon 9; c.C1156T, p. R386C in exon 10; c.C1417T, p. R473C in exon 12; c.C2572T, p. Q858SX in exon 20; c.2872 + 1G > A in intron 21; c.C3671T, p. S1224L in exon 28; c.3323-30G > A in intron 24)
Senat et al., 2001 [213]	Prenatal diagnosis of hydrocephalus-stenosis of the aqueduct of Sylvius by ultrasound in the first trimester of pregnancy. Report of two cases	CHI Poissy, France	2 Subjects	Caucasian	Case study	Corpus callosum agenesis, hypoplasia of pyramidal tract, spasticity, intellectual disability	Adducted thumbs	Obstructive	TGS	FAMA	De novo	Xq28 (p.Y589H in exon 14 of L1CAM)
Serikawa et al., 2014 [214]	Prenatal molecular diagnosis of X-linked hydrocephalus via a silent C924T mutation in the L1CAM gene	Niigata University Medical and Dental Hospital, Niigata, Japan	4 Subjects, 2 Parents	Japanese	Case study	Cortex thinning, cerebral palsy, intellectual disability, corpus callosum agenesis, aqueductal stenosis	Bilateral adducted thumbs	Obstructive	TGS	Sanger Sequencing	X-linked	Xq28 (c.C924T, p. G308G silent mutation in exon 8 of L1CAM)
Silan et al., 2005 [215]	A novel L1CAM mutation with L1 spectrum disorders	Abant Izzet Baysal University, Duzce School of Medicine, Duzce, Turkey	14 Subjects	Turkish	Case series	Corpus callosum agenesis, intellectual disability, spastic quadriplegia	Bilateral adducted thumbs	-	-	-	X-linked	Xq28 (c.C1375T, Q459X in exon 11 of L1CAM)
Stowe et al., 2018 [216]	Clinical Reasoning: Ventriculomegaly detected on 20-week anatomic fetal ultrasound	Baylor College of Medicine, Texas Children's Hospital, Houston	1 Subject	-	Case study	Aqueductal stenosis, diencephalic fusion, and brainstem dysplasia	Fisted thumbs	Obstructive	WES	Trio-based WES	X-linked, De novo	Xq28 (c.1703 + 5G > A in L1CAM)
Sullivan et al., 2020 [128]	Exome Sequencing as a Potential Diagnostic Adjunct in Sporadic Congenital Hydrocephalus	Yale School of Medicine, New Haven, Connecticut	475 Subjects	European, African American, south Asian	Case series	Hypotonia, cerebral palsy, epilepsy, white-matter hypoplasia, agenesis of the corpus callosum, macrocephaly	Bilateral adducted thumbs, skeletal abnormalities	Obstructive	WES	Sanger sequencing	X-linked, De novo	Xq28 (L1CAM mutations: p.W460C; p.W635R; c.1828 + 1G > A (localizing to intron 15); c.1546 + 1G > T (located in intron 13); p.E304X; p.V788F; c.806 + 1G > C (positioned in intron 8))
Sztriha et al., 2000 [217]	Novel missense mutation in the L1 gene in a child with corpus callosum agenesis, retardation, adducted thumbs, spastic paraparesis, and hydrocephalus	Faculty of Medicine and Health Sciences, United Arab Emirates University	1 Subject, 1 Parent, 1 Control	Arabic	Case study	Corpus callosum agenesis, intellectual disability, spastic paraparesis	Adducted thumbs	Communicating	TES	DGGE analysis	X-linked	Xq28 (c.G604T in exon 6 of L1CAM)
Sztriha et al., 2002 [218]	X-linked hydrocephalus: a novel missense mutation in the L1CAM gene	Faculty of Medicine and Health Sciences, United Arab Emirates University, Al Ain	1 Subject	Pakistani	Case study	Spastic diplegia, intellectual disability, multiple small gyri, markedly reduced white matter volume, agenesis of the corpus callosum, and lack of cleavage of the thalami	Adducted thumbs	Obstructive	-	-	X-linked	Xq28 (c.G1243C, p.A415P in exon 10 of L1CAM)
Takahashi et al., 1997 [219]	L1CAM mutation in a Japanese family with X-linked hydrocephalus: a study for genetic counseling	Asahikawa Medical College, Nishikagura, Japan	1 Subject, 2 Parents, 2 Sisters	Japanese	Case study	Intellectual disability, spastic quadriplegia	Bilateral adducted thumbs	Obstructive	TES	-	X-linked	Xq28 (1 bp deletion in exon 22 of L1CAM resulting in a premature stop codon)
Takechi et al., 1996 [220]	A deletion of five nucleotides in the L1CAM gene in a Japanese family with X-linked hydrocephalus	National Institute of Neuroscience, Tokyo, Japan	2 Subjects, 1 Sister	Japanese	Case study	Aqueduct of Sylvius, mental retardation, and spastic paraparesis	Bilateral clasped thumbs	-	TES	Dideoxy plasmid-based sequencing	X-linked	Xq28 (5 bp deletion in exon 8 of L1CAM)
Takenouchi et al., 2011 [221]	Hydrocephalus with Hirschsprung disease: severe end of X-linked hydrocephalus spectrum	Keio University School of Medicine, Tokyo, Japan	1 Subject	Japanese	Case study	Aqueductal stenosis, hypoplasia of the corpus callosum	Hirschsprung disease, frontal bossing, adducted thumbs	Obstructive	TGS	Mutation analysis, unspecified	X-linked, De novo	Xq28 (c.C61T, p.Q21X in exon 1 of L1CAM)
Tegay et al., 2007 [222]	Contiguous gene deletion involving L1CAM and AVPR2 causes X-linked hydrocephalus with nephrogenic diabetes insipidus	Stony Brook University Hospital, Stony Brook, New York, USA	1 Subject, Mother, Grandmother, 1 Control	Northern European	Case study	Hypotonia	Bilateral adducted thumbs, hirschsprung disease	Obstructive	WGS	GeneDX, microdeletion	X-linked	Xq28 (32.7 kb deletion and 90 bp insertion at the L1CAM-AVPR2 junction sequence (from L1CAM intron1 to AVPR2 exon2))
Van Camp et al., 1993 [223]	A duplication in the L1CAM gene associated with X-linked hydrocephalus	University of Antwerp-UIA, Belgium	25 Subjects, Controls used	The Netherlands, United Kingdom, USA, Israel, Germany, Hungary, Belgium	Case series	Stenosis of the aqueduct of Sylvius, intellectual disability, spastic para paresis of the lower extremities, aplasia or hypoplasia of the corpus callosum	Bilateral adducted thumbs	-	Southern Blotting	-	X-linked recessive	Xq28 (1.3 kb duplication in L1CAM)
Verhagen et al., 1998 [224]	Familial congenital hydrocephalus and aqueduct stenosis with probably autosomal dominant inheritance and variable expression	Canisius Wilhelmina Hospital, Nijmegen, Netherlands	12 Subjects	-	Case series	Septum pellucidum cavitation, aqueductal stenosis		Obstructive	TES	-	AD	No mutations in L1CAM
Vits et al., 1994 [225]	MASA syndrome is due to mutations in the neural cell adhesion gene L1CAM	University of Antwerp, Belgium	8 Subjects, 50 Controls	United States, the Netherlands, Mexico, UK, Germany	Case series	Intellectual disability	Adducted thumbs, shuffling gait, aphasia	-	TGS	SSCP	X-linked	Xq28 (p.D598N in exon 14 and p.H210Q in exon 6 of L1CAM)
Vos et el., 2010 [226]	Genotype–phenotype correlations in L1 syndrome: a guide for genetic counselling and mutation analysis	University Medical Centre Groningen, Hanzeplein 1, 9713 GZ Groningen, The Netherlands	367 Subjects, 3 Controls	Various	Case–Control	Aqueductal stenosis, intellectual disability, callosal agenesis	Adducted thumbs, shuffling gait, aphasia	Obstructive	TES	DGGE; direct sequencing; MLPA	X-linked recessive	Xq28 (L1CAM mutations: 23 missense mutations; 3 in-frame deletions/duplications; 18 splice site mutations; 14 nonsense mutations; 8 frame-shift mutations; 1 duplication of exons 2–10; 1 deletion of the entire gene; c.C645T within exon 6 of L1CAM)
Wilson et al., 2009 [227]	Prenatal identification of a novel R937P L1CAM missense mutation	University of Oklahoma Health Sciences Center, Oklahoma City, Oklahoma, USA	2 Subjects	Caucasian	Case study	Aqueductal stenosis, agenesis or hypoplasia of the corpus callosum and corticospinal tracts, intellectual disability, spastic paraplegia	Adducted thumbs, short femurs, right clubbed foot	Obstructive	TGS	bidirectional DNA sequencing	X-linked	Xq28 (c.G2809C, p.R937P in exon 21 of L1CAM)
Xie et al., 2018 [228]	Two novel pathogenic variants of L1CAM gene in two fetuses with isolated X-linked hydrocephaly: A case report	Guangxi Maternal and Child Health Hospital, Nanning, Guangxi, P.R. China	2 Subjects, 4 Parents, 100 Controls	Chinese	Case–Control	Isolated hydrocephalus		-	TES	Sanger sequencing	X-linked	Xq28 (c.C998T, p.P333L and c.G2362T, p.V788F in L1CAM)
Yamasaki et al., 2011 [229]	Prenatal molecular diagnosis of a severe type of L1 syndrome (X-linked hydrocephalus)	Osaka National Hospital, National Hospital Organization, Osaka City, Japan	14 Subjects	Japanese	Case series	Intellectual disability, spastic paraplegia	Adducted thumbs, shuffling gait, aphasia	-	TGS	Direct sequencing	X-linked	Xq28 (L1CAM mutations: c.G1829-1C 1 bp downstream from the 5′ of intron 14; ACC (817–819) nucleotide deletion in exon 8, deletion of T at amino acid position 273; c.C1146A, p.Y382X in exon 10)

Amplification created restriction site (ACRS). Array comparative genomic hybridization (aCGH). Atrial Septal Defect (ASD). Autosomal Recessive (AR). Central Nervous System (CNS), Copy number variant (CNV). Deep tendon reflexes (DTR). Denaturing gradient gel electrophoresis (DGGE). Fluorescein isothiocyanate (FITC). Fluorescence assisted mismatch analysis (FAMA). Mental retardation, aphasia, shuffling gait, and adducted thumbs syndrome (MASA syndrome). Multiplex ligation dependent probe amplification (MLPA). Restriction endonuclease fingerprinting (REF). Single-strand conformation polymorphisms (SSCP). Targeted exome sequencing (TES). Targeted genome sequencing (TGS). Ventricular septal defect (VSD). Whole exome sequencing (WES). Whole genome sequencing (WGS). Internal carotid artery (ICA)

### Dandy walker malformation

Dandy Walker malformation is a cerebellar structural anomaly that can impede CSF flow but can also be related to primary brain developmental alterations and contribute to HC development. Missense mutations are found in forkhead box C1 (*FOXC1*), fukutin (*FKTN*), laminin subunit gamma 1 (*LAMC1*), sphingosine-1-phosphate phosphatase 2 (*SGPP2*), and exocyst complex component 3 like 2 (*EXOC3L2*). Nonsense mutations are found in FKTN, nidogen 1 (*NID1*), and potassium channel tetramerization domain containing 3 (*KCTD3*). SIL1 nucleotide exchange factor (*SIL1*) displayed a nonstop mutation and carnitine palmitoyltransferase 2 (*CPT2*) displayed a deletion-insertion variant. Additional mutations included Zic family member 2 (*ZIC2*) and Zic family member 5 (*ZIC5).* Deletions were found in lysine methyltransferase 2D (*KMT2D*), chromosome 2 (2q36.1), chromosome 3 (3q25.1), chromosome 6 (6p24.1, 6p25.3), chromosome 7 (7p21.3), chromosome 8 (8q21), chromosome 12 (12q24), chromosome 13 (13q32), and chromosome 16 (16q21). The deletion of 8p21 resulted in the downregulation of fibroblast growth factor 17 (*FGF17*). Duplications were found in chromosome 6 (6p25.3), chromosome 7 (7p21.3), and chromosome 12 (12q24). In addition, EXOC3L2 regulates vesicular trafficking at synapses and cell polarity; a mutation within this gene locus can impact normal brain development [44]. *KCTD3* is also highly expressed in the brain and kidneys and regulates ion channels such as hyperpolarization activated cyclic nucleotide-gated channel 3 (HCN3) [45]. SIL1 is a glycoprotein that regulates protein trafficking into the ER and ATPase activity, suggesting a mutated implication in protein folding through development [46, 47]. A patient with a mutation in *CPT2*, an enzyme responsible for breaking down long chain fatty acids, suggests a role of metabolic enzymes in the genetic susceptibility of HC secondary to Dandy Walker malformation [48]. Thus, Dandy Walker malformation related HC may be caused by a wide variety of genes involved in many biological processes. These data are summarized in Table 4.

**Table 4:** Dandy walker malformation

Citation	Title	Author affiliation	Case #	Ancestry	Study design	CNS phenotype	Non-CNS phenotype	Type of hydrocephalus	Genetic methodology	Genetic analysis	Inheritance	Genetic findings
Aldinger et al., 2009 [230]	FOXC1 is required for normal cerebellar development and is a major contributor to chromosome 6p25.3 Dandy-Walker malformation	University of Chicago, Chicago, Illinois, USA	18 Subjects, 2 Controls	-	Case series	Dandy walker—cerebellar malformations	Ocular abnormalities	Communicating	WES	Trio-based sequencing	De novo, maternal translocation, Mosaicism	6p25.3 (p.S82T and p.S131L in FOXC1)
Arora et al., 2019 [231]	Prenatal presentation of a rare genetic disorder: a clinical, autopsy and molecular correlation	Sir Ganga Ram Hospital. New Delhi, India	1 Subject	-	Case study	Dandy walker—cerebellar malformations, callosal agenesis	Talipes equinovarus, renal cysts	Communicating	WES	Trio-based sequencing	AR	9q31.2 (c.C411A. p. C137X in exon 5 of FKTN)
Chen et al., 2009 [232]	A 12 Mb deletion of 6p24.1– > pter in an 18-gestational-week fetus with orofacial clefting, the Dandy-Walker malformation and bilateral multicystic kidneys	Mackay Memorial Hospital, Taipei, Taiwan	1 Subject	-	Case study	Craniosynostosis	Tracheal stenosis, midface hypoplasia, ocular proptosis and digital malformations	Communicating	Cytogenetics	Karyotyping, aCGH	De novo	6pter/6p24.1 (12 Mb deletion)
Darbro et al., 2013 [233]	Mutations in extracellular matrix genes NID1 and LAMC1 cause autosomal dominant Dandy-Walker malformation and occipital cephaloceles	The University of Iowa, Iowa City, Iowa, USA	7 Subjects, 348 Controls	Indian, Vietnamese	Case study	Dandy walker malformation, variable cerebellar hypoplasia, meningeal anomalies, and occipital skull defects	-	-	WES	Massively parallel sequencing, Sanger sequencing	AD	1q25.3 (c.C2237T, p.T746M in LAMC1); 1q42.3 (c.C1162T, p.Q388X in NID1)
Faqeih et al., 2017 [234]	Phenotypic characterization of KCTD3-related developmental epileptic encephalopathy	Children's Specialized Hospital, King Fahad Medical City, Riyadh, Saudi Arabian	7 Subjects	-	Case series	Seizures, poor muscle control and tone, dandy walker malformation	Renal distention, bilateral hip dislocation, scoliosis	Communicating	WES	Sanger Sequencing	De novo	1q41 (c.1036_1073del, p.P346Tfs*4; c.C166T, p.R56X in KCTD3)
Gai et al., 2016 [235]	Novel SIL1 nonstop mutation in a Chinese consanguineous family with Marinesco-Sjögren syndrome and Dandy-Walker syndrome	Central South University, 110 Xiangya Road, Changsha, Hunan 410,078, China	2 Subjects, Matched Controls used	Chinese	Case study	Mild intellectual disability, hypotonia, ataxia, dysarthria, strabismus, and dandy walker malformation	Cubitus valgus	-	WES	Sanger sequencing	AR	5q31.2 (nonstop mutation in SIL1)
Guo et al., 2020 [236]	Hypoglycemia and Dandy-Walker variant in a Kabuki syndrome patient: a case report	Xingtai People's Hospital, Xingtai, Hebei, China	1 Subject, 2 Parents	Chinese	Cases study	Dandy walker—cerebellar malformations	Persistent hypoglycemia, elongated palpebral fissures with eversion of the lower lateral eyelids and prominent ears	Communicating	WES	Sanger sequencing	De novo	12q13.12 (c.12165del, p.E4056Sfs*10 in exon 39 of KMT2D)
Jalali et al., 2008 [237]	Linkage to chromosome 2q36.1 in autosomal dominant Dandy-Walker malformation with occipital cephalocele and evidence for genetic heterogeneity	Northwestern University Feinberg School of Medicine, Chicago, IL, USA	19 Subjects	Vietnamese- American and Brazilian	Case series	Dandy walker malformation, occipital encephalocele	Prominent forehead, mildly downturned vermilion border of the upper lip, deep-set eyes and flat philtrum, minimal high frequency hearing loss	Communicating	TGS, cytogenetics	SNP genotyping, multipoint linkage analysis, G-banded karyotype analysis and FISH	AD	2q36.1 (silent mutation of SGPP2; insertion/deletion 85 bp upstream of ACSL3 exon 4)
Liao et al., 2012 [238]	Prenatal diagnosis and molecular characterization of a novel locus for Dandy-Walker malformation on chromosome 7p21.3	Guangzhou Women and Children's Medical Center, Guangzhou Medical College, Guangzhou, Guangdong, China	4 Subjects	-	Case series	Dandy walker—cerebellar malformations	Ocular hypertelorism, cardiac anomalies, talipes valgus, syndactyly	-	WGS, cytogenetics	aCGH, FISH	De novo	7p21.3 (de novo adjacent microdeletion/duplication)
Linpeng et al., 2018 [239]	Diagnosis of Joubert Syndrome 10 in a Fetus with Suspected Dandy-Walker Variant by WES: A Novel Splicing Mutation in *OFD1*	Central South University, Changsha, Hunan, China	3 Subjects, 1 Control	Chinese	Case study	Hypoplastic cerebellum and absent vermis	Bilateral postaxial polydactyly	-	WES, cytogenetics	Karyotype; microarray; CNV; FISH; Sanger sequencing	Maternal	8q21 (4.9 Mb heterozygous deletion at 8q21.13-q21.3); Xp22.2 (c.T2488 + 2C, resulting in an abnormal skipping of exon 18 in OFD1)
MacDonald, Holden 1985 [240]	Duplication 12q24––qter in an infant with Dandy-Walker syndrome	Queen's University, Kingston, Ont., Canada	1 Subject	-	Case study	Dandy walker—cerebellar malformations		-	Cytogenetics	-	Paternal	12q24 (duplication 12q24 to qter)
Mademont-Soler et al., 2010 [241]	Description of the smallest critical region for Dandy-Walker malformation in chromosome 13 in a girl with a cryptic deletion related to t(6;13)(q23;q32)	Servei de Bioquímica i Genètica Molecular, Hospital Clínic, Barcelona, Spain	1 Subject, Controls used	-	Case study	Dandy walker—cerebellar malformations	Iris coloboma, profound hearing loss, and hyperlaxity of skin and joints	Obstructive	WGS, cytogenetics	G-banded chromosome analysis, aCGH, CNV analysis, FISH	De novo	Karyotype 46,XX,t(6;13)(q23;q32); 2.47 Mb deletion of band 13q32; 4 Mb deletion of 16q21
Matsukura et al., 2017 [242]	MODY3, renal cysts, and Dandy-Walker variants with a microdeletion spanning the HNF1A gene	Saiseikai Toyama Hospital	1 Subject	Japanese	Case study	Intellectual disability, dandy walker malformation	Glycosuria, developmental delay, renal cysts	-	TGS, cytogenetics	MLPA; direct sequencing, aCGH	De novo	5.6 Mb deletion of 12q24.22–12q24.31 in HNF1A
Mimaki et al., 2015 [243]	Holoprosencephaly with cerebellar vermis hypoplasia in 13q deletion syndrome: Critical region for cerebellar dysgenesis within 13q32.2q34	Graduate School of Medicine, The University of Tokyo, Japan	2 Subjects	-	Case series	Cerebellar hypoplasia, hypoplastic optic nerve	Upslanted palpebral fissures, hypertelorism, low-set malformed ears, a broad prominent nasal bridge, micrognathia, micropenis, hypospadias, bifid scrotum, and a low-level imperforate anus, ventral septal defect	Obstructive	Cytogenetics	G-banding, FISH, aCGH	De novo	13q32.3 (ZIC2 and ZIC5)
Shalata et al., 2019 [244]	Biallelic mutations in EXOC3L2 cause a novel syndrome that affects the brain, kidney and blood	Pediatrics and Medical Genetics and The Simon Winter Institute for Human Genetics, Bnai Zion Medical Center, Haifa, Israel	4 Subjects, 2 Control	-	Case series	Hypotonia, dandy-walker malformation	Panhypopituitarism, hearing impairment, cataracts and congenital glaucoma, renal failure, buphthalmos, corneal ectasia, narrow ears canal, high arched palate and undescended testes	Obstructive	WES, cytogenetics	aCGH/SNP array, microarray analysis, Sanger Sequencing	-	19q13.32 (c.T122A, p.L41Q in EXOC3L2)
Sudha et al., 2001 [245]	De novo interstitial long arm deletion of chromosome 3 with facial dysmorphism, Dandy-Walker variant malformation and hydrocephalus	Health Sciences Centre, University of Manitoba, Winnipeg, Canada	1 Subject, 2 Parents	German-Swiss	Case study	Dandy walker—cerebellar malformations, macrocrania	Coarse facial features, developmental delay	Obstructive	Cytogenetics	Karyotyping, FISH analysis utilizing WCP	De novo	46,XX,del(3)(q25.1q25.33) de novo
Traversa et al., 2019 [246]	Prenatal whole exome sequencing detects a new homozygous fukutin (FKTN) mutation in a fetus with an ultrasound suspicion of familial Dandy-Walker malformation	Fondazione IRCCS Casa Sollievo della Sofferenza, Laboratory of Clinical Genomics, San Giovanni Rotondo (FG), Italy	1 Subject, 2 Parents	Italian	Case study	Dandy walker—cerebellar malformations		Obstructive	WES	Sanger sequencing	-	9q31.2 (c.G898A, p.G300R in FKTN)
Yahyaoui et al., 2011 [48]	Neonatal carnitine palmitoyltransferase II deficiency associated with Dandy-Walker syndrome and sudden death	Clinical Laboratory, Carlos Haya University Hospital, Málaga, Spain	1 Subject	Moroccan	Case study	Dandy-Walker malformation	Hypoketotic hypoglycemia, severe hepatomuscular symptoms, cardiac abnormalities	-	TGS	-	-	1p32.3 (c.534_558del25bpinsT, p.L178_I186delinsF of CPT2)
Zaki, et al., 2015 [247]	Dandy-Walker malformation, genitourinary abnormalities, and intellectual disability in two families	National Research Centre, Cairo, Egypt	3 Subjects	Egyptian	Case series	Intellectual disability, Dandy-Walker malformation	Genitourinary abnormalities, hearing deficit	Obstructive	TGS, cytogenetics	aCGH, CNV analysis	AR	Genetic analysis unrevealing
Zanni et al., 2011 [248]	FGF17, a gene involved in cerebellar development, is downregulated in a patient with Dandy-Walker malformation carrying a de novo 8p deletion	Bambino Gesù Pediatric Hospital, 4 Piazza S. Onofrio, Rome	1 Subject, 3 Controls	-	Case study	Hypotonia	Motor delay, gastroesophageal reflux and frequent gastrointestinal and respiratory infections, joint laxity, facial deformity	Obstructive	WGS, cytogenetics	aCGH, FISH analysis using a locus-specific probe	De novo	8p21.3 (2.3 Mb deletion in 8p21.2-8p21.3; reduced levels of FGF17)

Array comparative genomic hybridization (aCGH). Autosomal Dominant (AD). Autosomal Recessive (AR). Copy number variant (CNV). Fluorescence In Situ Hybridization (FISH). Multiplex ligation dependent probe amplification (MLPA). Next generation sequencing (NGS). Single nucleotide polymorphisms (SNP). Targeted exome sequencing (TES). Targeted genome sequencing (TGS). Whole chromosome probes (WCP). Whole exome sequencing (WES). Whole genome sequencing (WGS)

### Ciliopathy

Genes involved in cilia function that are associated with HC are summarized in Table 5**.** Primary cilia dysfunction has been demonstrated to play a role in HC with numerous Mendelian ‘ciliopathies’ resulting in HC. Missense mutations were observed in Meckel-Gruber syndrome gene (*MKS3*), MKS transition zone complex subunit 1 (*MKS1*), intraflagellar transport 43 (*IFT43*), WD repeat domain 35 (*IFT121*), coiled-coil and C2 domain containing 2A (*CC2D2A*), transmembrane protein 216 (*TMEM216*), PKHD1 ciliary IPT domain containing fibrocystin/polyductin (*PKHD1*), intestinal cell kinase (*ICK*), exon 14 of KIAA0586, exons 4 and 13 of centrosomal protein 83 (*CEP83*), exons 6, 11, 12, 20, 23, 24, 28, 29, 32, and 36 of SET binding factor 2 (*SBF2*), exon 9 of zinc finger E-box binding homeobox 1 (*ZEB1*), and exon 5 of G protein subunit alpha i2 (*GNAI2*). Nonsense mutations were identified in *CC2D2A*, *IFT121*, forkhead box J1 (*FOXJ1*), exon 2 of *KIAA0586*, exon 3 of centrosomal protein 55 (*CEP55*), exons 3, 4, 7, and 13 of *CEP83*, and exon 11 of *SBF2*. Deletions and duplications resulting in frameshift mutations were found in *CC2D2A*, *MKS3*, *MKS1*, dynein axonemal intermediate chain 2 (*DNAI2*), *IFT121*, *FOXJ1*, exon 5 and 17 of *CEP83*, and exon 4 of *ZEB1*. Exon 2 was deleted in WD repeat-containing protein 16 (*WDR16*). Additional mutations were found in WD repeat domain 93 (*WDR93*). Loss of *MKS3* and *MKS1* are associated with ciliary shortening and dysfunction, suggesting a role in primary ciliary development. *TMEM216* also contributes to ciliary development through apical polarization and formation and may result in Joubert, Meckel and related syndromes [49]. IFT43 and IFT121 maintain cilium organization and regulate intraflagellar transport in interaction with the IFT-A complex [50]. In addition, CEP83 also interacts with IFT proteins and guides vesicular docking ciliogenesis [51]. One patient was identified with a mutation in *DNAI2*, a component of the outer dynein arm complex (ODA), which is involved in cilia motility [52]. ZEB1, SBF2, and GNAI2 are involved in other signaling pathways previously identified in association with HC [53].

**Table 5:** Ciliopathy

Citation	Title	Author affiliation	Case #	Ancestry	Study design	CNS phenotype	Non-CNS phenotype	Type of hydrocephalus	Genetic methodology	Genetic analysis	Inheritance	Genetic findings
Alby et al., 2015 [249]	Mutations in KIAA0586 Cause Lethal Ciliopathies Ranging from a Hydrolethalus Phenotype to Short-Rib Polydactyly Syndrome	Paris Descartes University, Sorbonne Paris Cité and Imagine Institute, Paris, France	8 Subjects, Controls used	Romania, Hungary, Kosovo, Lebanon	Case study	Anencephaly or large occipital meningocele to vermian agenesis, associated with brainstem anomalies	Cleft lip and palate, polysyndactyly, preaxial polydactyly of the feet	-	TES	NGS; CNV	AR	14q23.1 (c.C230G, p.S77X in exon 2 and c.G1815A of exon 14 in KIAA0586)
Al-Shroof et al., 2001 [250]	Ciliary dyskinesia associated with hydrocephalus and mental retardation in a Jordanian family	Houston Medical Center, Warner Robins, GA, USA	4 Subjects, 5 Family Members	Jordanian	Case study	Intellectual disability	Growth delay	Communicating	Chromosome analysis	Haplotype analysis	AR	-
Bachmann-Gagescu et al., 2012 [251]	Genotype–phenotype correlation in CC2D2A-related Joubert syndrome reveals an association with ventriculomegaly and seizures	University of Washington, Seattle, Washington, USA	20 Subjects, Controls used	-	Case series	Intellectual impairment, hypotonia, ataxia, molar tooth sign	Retinal dystrophy, chorioretinal coloboma, cystic kidney disease, liver fibrosis and polydactyly	Obstructive	TGS	Variant analysis; microsatellite marker assay; a-CGH, SNP	AR	4p15.32 (CC2D2A mutations: p.S117R; p.IVS11(+ 1); p.S423Gfs*19; p.K507E; p.L559P; p.R950X; p.R1019X; p.R1049X; p.V1097Ffs*1; p.V1045A; p.Q1096H; p.T1116M; p.P1122S; p.V1151A; p.IVS29(-1); p.V1298Ffs*16; p.IVS30(-3); p.E1393Efs*1; p.R1284C; p.R1284H; p.R1330Q; p.V1430A; p.R1528C; p.D1556V; p.S1615Lfs*15)
Bondeson et al., 2017 [252]	A nonsense mutation in CEP55 defines a new locus for a Meckel-like syndrome, an autosomal recessive lethal fetal ciliopathy	Uppsala University, Science for Life Laboratory, Uppsala, Sweden	5 Subjects, Controls used	Swedish	Case study	Liquified skull, cerebral cysts, encephalocele	Bilateral club foot, renal cysts, neck hygroma, single umbilical artery	-	WES	Sanger Sequencing; haplotype analysis	AR	10q23.33 (c.C256T, p.R86X in exon 3 of CEP55)
Boycott et al., 2007 [253]	Meckel syndrome in the Hutterite population is actually a Joubert-related cerebello-oculo-renal syndrome	Alberta Children's Hospital and University of Calgary, Calgary, Alberta, Canada	10 Subjects	Hutterite	Case series	Developmental delay, hypotonia, ataxia, abnormal breathing pattern, nystagmus, strabismus	Growth failure, retinal colobomas, post-axial polydactyly, cystic kidneys, abnormalities in renal function, hypertension, occipital encephalocele, posterior fossa fluid collections	Obstructive	TGS	Microsatellite marker assay	AR	Genetic analysis unrevealing
Dawe et al., 2007 [254]	The Meckel-Gruber Syndrome proteins MKS1 and meckelin interact and are required for primary cilium formation	University of Oxford, South Parks Road, Oxford OX1 3RE, UK	3 Subjects, 2 Controls	-	Case series	Dandy-Walker malformation, agenesis of the corpus callosum, microcephaly, rhombic roof dysgenesis and prosencephalic dysgenesis	Fibrocystic liver changes, polydactyly, cleft lip/palate, laterality defects and congenital heart malformations including dextrocardia, shortening and bowing of the long tubular bones and abnormal development of the male genitalia	-	In situ hybridization studies	Direct sequencing via dideoxy chain termination method	-	8q22.1 (c.647delA, p.E216fsX221 and c.A1127C, p.Q376P in MKS3); 17q22 (c.1448_1451dupCAGG duplication, p.T485fsX591 in MKS1)
Duran et al., 2017 [255]	Mutations in IFT-A satellite core component genes *IFT43* and *IFT121* produce short rib polydactyly syndrome with distinctive campomelia	David Geffen School of Medicine at the University of California at Los Angeles, Los Angeles, CA 90095 USA	3 Subjects, Control cells used	European	Case series	Isolated hydrocephalus	Long narrow chest, markedly shortened long bones, polydactyly and, often, cardiac, gastrointestinal, and genitourinary abnormalities	-	TES	Variant analysis, sanger sequencing	AR	14q24.3 (c.T2A, p.M1K and c.T535C, p.W179R in IFT43); 2p24.1 (IFT121 mutations: c.G1433A, p.R478K; c.C1579T, p.Q527X; c.G932T, p.W311L; c.1501delC, p.Q501Kfs*10)
Edvardson et al., 2010 [256]	Joubert syndrome 2 (JBTS2) in Ashkenazi Jews is associated with a TMEM216 mutation	Hebrew University Medical Center, Jerusalem, Israel	13 Subjects, Controls used	Ashkenazi Jewish	Case series	Mid hindbrain malformation, hypotonia, cerebellar ataxia, and developmental delay	Oculomotor apraxia, abnormal breather patterns, retinal degeneration, renal anomalies, ocular colobomas and liver abnormalities	Obstructive	TGS	SNP; array-based hybrid selection; deep sequencing	-	11q12.2 (c.G35T, p.R12L in TMEM216)
Failler et al., 2014 [257]	Mutations of CEP83 cause infantile nephronophthisis and intellectual disability	Laboratory of Inherited Kidney Diseases, 75,015 Paris, France	1,255 Subjects, Controls used	European; Turkish; Latino	Case series	Intellectual disability	Renal malformation, retinitis pigmentosa, intellectual disability, cerebellar ataxia, bone anomalies, liver fibrosis	-	TES	NGS; sanger sequencing	AR	12q22 (CEP83 mutations: c.C121T, p.R41X in exon 3; c.C241T, p.Q81X in exon 4; c.T260T, p.L87P in exon 4; c.335_352del, p.P112_L117del in exon 5; c.C625T, p.R209X in exon 7; c.C1530A, p.C510X in exon 13; c.G1532C, p.R511P in exon 13; c.2007del, p.E669Dfs*14 in exon 17; c.2050_2052del, p.E684del in exon 17; c.2075_2077del, p.Q692del in exon 17)
Kosaki et al., 2004 [258]	Absent inner dynein arms in a fetus with familial hydrocephalus-situs abnormality	Keio University School of Medicine, Tokyo, Japan	3 Subjects	-	Case study	Isolated hydrocephalus	Situs inversus, micrognathia, ulnar deviation of the fingers with absent distal interphalangeal creases on fingers 2–4, lung abnormalities, and rocker-bottom feet	-	TES	Direct sequencing	AR	-
Mei et al., 2021 [259]	Genetic etiologies associated with infantile hydrocephalus in a Chinese infantile cohort	Children's Hospital of Fudan University, National Children's Medical Center, Shanghai, China	110 Subjects, 300 Controls	Chinese	Case series	Isolated hydrocephalus		-	WES	NGS; variant analysis	-	11p15.4 (SBF2 mutations: c.G1171A, p.A391T in exon 12; c.A3877G, p.K1293E in exon 29; c.A3754T, p.S1252C in exon 28; c.A3056T, p.Q1019L in exon 24; c.C5037T, p.R1679R in exon 36; c.C1066T, p.R356X in exon 11; c.G1067T, p.R356L in exon 11; c.A2390G, p.Y797C in exon 20; c.A2813G, p.E938G in exon 23; c.T527G, p.L176W in exon 6; c.A4328C, p.E1443A in exon 32); 10p11.22 (ZEB1 mutations: c.444_461delinsG, p.G150Wfs*3 in exon 4; c.479_480delinsA, p.N160Kfs*26 in exon 4; c.G2995C, p.E999Q in exon 9); 3p21.31 (c.A465-8C in exon 5 of GNAI2)
Nabhan et al., 2014 [260]	Case Report: Whole-exome analysis of a child with polycystic kidney disease and ventriculomegaly	Kasr Al Ainy School of Medicine, Center of Pediatric Nephrology and Transplantation, Cairo University, Egyptian Group for Orphan Renal Diseases, Cairo, Egypt	1 Subject, 2 Parents	-	Case study	Macrocephaly	Bilateral enlarged and palpable kidneys, systemic hypertension	-	WES	Sanger sequencing, segregation analysis	AR	6p12.3 (c.G3367A, p.G1123S in PKHD1); 17q22 (c.G368A, p.R123Q in MKS1)
Oud et al., 2016 [261]	A novel ICK mutation causes ciliary disruption and lethal endocrine-cerebro-osteodysplasia syndrome	Radboud Institute for Molecular Life Sciences, Radboud University Medical Centre, PO-Box 9101, 6500 HB Nijmegen, The Netherlands	4 Subjects, 2 Controls	Turkish	Case study	Absence of septum pellucidum	Genital anomalies, ventral septal defect, renal abnormalities, cystic hygroma, scalp edema ascites, very short tubular bones and polydactyly of hands and feet, and short ribs	-	WES; genotyping	IBD mapping; CNV; Sanger sequencing	-	6p12.1 (c.G358T, p.G120C in ICK)
Rocca et al., 2020 [262]	A novel genetic variant in DNAI2 detected by custom gene panel in a newborn with Primary Ciliary Dyskinesia: case report	University of Padova, Via Giustiniani, Padova, Italy	1 Subject	Moroccan	Case study	Isolated hydrocephalus	Situs inversus, respiratory infections	Communicating	TGS, cytogenetics	NGS; aCGH; CNV	-	17q25.1 (6.9 kb deletion in of DNAI2)
Wallmeier et al., 2019 [263]	De Novo Mutations in FOXJ1 Result in a Motile Ciliopathy with Hydrocephalus and Randomization of Left/Right Body Asymmetry	University Children's Hospital Muenster, 48,149 Muenster, Germany	6 Subjects, Controls used	Germany, USA	Case series	Isolated hydrocephalus	Chronic destructive airway disease, and randomization of left/right body asymmetry	Obstructive	WES	Sequencing, unspecified	De novo	17q25.1 (FOXJ1 mutations: c.G901T, p.E301X; c.868_871dup, p.T291Kfs*12; c.C826T, p.Q276X; c.967delG, p.E323Sfs*10; c.939delC, p.I314Sfs*19)

Array comparative genomic hybridization (aCGH). Autosomal Recessive (AR). Copy number variant (CNV). Identical-By-Descent (IBD). Next generation sequencing (NGS). Single nucleotide polymorphisms (SNP). Targeted exome sequencing (TES). Targeted genome sequencing (TGS). Whole exome sequencing (WES). Whole genome sequencing (WGS)

### PI3K-Akt-mTOR

Genes involved in PI3K-Akt-mTOR cell signaling pathway underlying HC are summarized in Table 6. Missense mutations were identified in ring finger protein 125 (*RNF125*), HECT and RLD domain containing E3 ubiquitin protein ligase family member 1 (*HERC1*), AKT serine/threonine kinase 3 (*AKT3*), mechanistic target of rapamycin kinase (*mTOR*), phosphatase and tensin homolog (*PTEN*), cyclin D2 (*CCND2*), phosphatidylinositol-4,5-bisphosphate 3-kinase catalytic subunit alpha (*PIK3CA*) (exon 18 and others), phosphoinositide-3-kinase regulatory subunit 2 (*PIK3R2*) (exon 13 and others), and platelet derived growth factor receptor beta (*PDGFRB*) (exon 12 and others). Deletions were observed in *PIK3CA*, and nonsense mutations were seen in *PTEN* [54]. Deletions in chromosome 1 (1q42.3-q44) resulted in the deletion of AKT serine/threonine kinase 3 (*AKT3*). Additional genetic mutations implicated in this pathway included those in tripartite motif containing 71 (*TRIM71*), SWI/SNF related matrix associated, actin dependent regulator of chromatin (*SMARCC1*), forkhead box J1 (*FOXJ1*), formin 2 (*FMN2*), patched 1 (*PTCH1*), and FXYD domain containing ion transport regulator 2 (*FXYD2*). Multiple genes within the PI3K-AKT-MTOR pathway highlight convergence on molecular mechanisms conferring risk to HC. Murine models have demonstrated the role of *HERC1*, which codes for an E3 ubiquitin ligase, to affect Purkinje cell physiology and mTOR activity [55]. *TRIM71* and *SMARCC1* are expressed within the ventricles and epithelium of mice brains (determined via in situ hybridization) suggesting that a mutation within this gene locus can affect this region may lead to HC [8]. Mutations in *FOXJ1* and *FMN2* have been shown to alter neuroepithelial integrity and lead to HC in mice [56, 57]. Mice harboring mutations in *PTCH1* also display defects in ependymal cell integrity [58]. Thus, mutations within many genes converging on PI3K-Akt-mTOR signaling have been widely implicated in HC pathophysiology.

**Table 6:** PI3K-Akt-MTOR

Citation	Title	Author affiliation	Case #	Ancestry	Study design	CNS phenotype	Non-CNS phenotype	Type of hydrocephalus	Genetic methodology	Genetic analysis	Inheritance	Genetic findings
Cappuccio et al., 2019 [264]	Severe presentation and complex brain malformations in an individual carrying a CCND2 variant	Federico II University, Naples, Italy	1 Subject, 2 Parents	-	Case study	Infantile spasms, siezures, developmental delay, bilateral PMG, white matter hypoplasia, fenestration of the septum pellucidum and hypoplasia of the anterior and posterior commissures, hippocampal hypoplasia and malrotation, hypoplastic thalami and lentiform nuclei malrotation of the vermis, brainstem hypoplasia	Bilateral postaxial polydactyly, patent foramen ovale and ductus arteriosus	-	TGS	NGS, sanger sequencing	De novo	12p13.32 (c.C839T, p.T280I in CCND2)
Jin et al., 2020 [10]	Exome sequencing implicates genetic disruption of prenatal neuro-gliogenesis in sporadic congenital hydrocephalus	The Rockefeller University, New York, NY, USA	381 Subjects, 1,798 Controls	-	Case–Control	Congenital hydrocephalus	-	Obstructive, Communicating	WES	CNV; sanger sequencing	*De-novo*	3q26.32 (PIK3CA mutations: p.D350N; p.E365K; p.G914R; p.R770Q; p.N345S); 10q23.31 (PTEN mutations: p.Y16X; p.R130Q; p.R335X; p.S305N); 1p36.22 (MTOR mutations: p.E1799K; p.M304T; p.R769C; p.R1161G; p.R1170C; p.H1782R); Mutations in 3p22.3 (TRIM71), 3p21.31 (SMARCC1), 17q25.1 (FOXJ1), 1q43 (FMN2), 9q22.32 (PTCH1) and 11q23.3 (FXYD2)
Maguolo et al., 2018 [265]	Clinical pitfalls in the diagnosis of segmental overgrowth syndromes: a child with the c.2740G > A mutation in PIK3CA gene	University Hospital of Verona, Verona, Italy	1 Subject	Italian	Case study	Cerebellar tonsillar ectopia, a markedly thick corpus callosum, and white matter abnormalities	Lateralized overgrowth (segmental overgrowth syndrome)	-	TGS	Targeted NGS	-	3q26.32 (c.G2740A, pG914R in exon 18 of PIK3CA)
Maini et al., 2018 [266]	A Novel CCND2 Mutation in a Previously Reported Case of Megalencephaly and Perisylvian Polymicrogyria with Postaxial Polydactyly and Hydrocephalus	Azienda Unità Sanitaria Locale, Arcispedale Santa Maria Nuova, IRCCS, Reggio Emilia, Italy	1 Subject, Controls used	-	Case study	Intellectual disability, seizures	Aphasia, postaxial polydactyly	-	WES	Sanger sequencing, direct sequencing	-	12p13.32 (c.C839T, p.T280I in CCND2)
McDermott et al., 2018 [267]	Hypoglycaemia represents a clinically significant manifestation of PIK3CA- and CCND2-associated segmental overgrowth	St Mary's Hospital, Central Manchester University Hospitals, NHS Foundation Trust Manchester Academic Health Sciences Centre, Manchester, UK	6 Subjects	-	Case series	Polymicrogyria	Polydactyly, capillary malformation, endocrine abnormalities	-	TGS	NGS; sanger sequencing	-	3q26.32 (PIK3CA mutations: c.G1048A, p.D350N; c.G2176A, p.E726K; c.G263A, p.R88Q); 12p13.32 (c.C841G, p.P281R in CCND2)
Mirzaa et al., 2015 [268]	Characterisation of mutations of the phosphoinositide-3-kinase regulatory subunit, PIK3R2, in perisylvian polymicrogyria: a next-generation sequencing study	University of Washington, Seattle, WA, USA; Center for Integrative Brain Research, Seattle Children's Research Institute, Seattle, WA, USA	20 Subjects, Controls used	USA	Case series	Polymicrogyria, seizures	Oromotor weakness	-	WES	AD assay; smMIPs; amplicon sequencing; sanger sequencing	De novo*,* maternal,	19p13.11 (c.G1117A, p.G373R and c. A1126G, p.K376E in PIK3R2)
Mirzaa, et al. 2013 [269]	Megalencephaly syndromes and activating mutations in the PI3K-AKT pathway: MPPH and MCAP	Center for Integrative Brain Research, University of Washington, Seattle Children's Research Institute, Seattle, WA, USA	50 Subjects	-	Case series	Cerebellar tonsillar ectopia or Chiari malformation, cortical brain abnormalities, macrocephaly	Postaxial polydactyly	-	WES	Sanger sequencing; REF; targeted ultra-deep sequencing	De novo	19p13.11 (p.G373R in PIK3R2); 1q43-q44 (p.R465W and p.N229S in AKT3); 3q26.32 (PIK3CA mutations: c.G241A, p.E81K; c.G263A, p.R88Q; c.G1090A, p.G364R; c.G1093A, p.E365K; c.G1133A, p.C378Y; c.1359_1361del, p.E453del; c.G1633A, p.E545K; c.G2176A, p.E726K; c.G2740A, p.G914R; c.A3062G, p.Y1021C; c.A3073G, p.T1025A; c.C3104T, p.A1035V; c.G3129T, p.M1043I; c.C3139T, p.H1047Y; c.G3145A, p.G1049S)
Ortega-Recalde et al., 2015 [270]	Biallelic HERC1 mutations in a syndromic form of overgrowth and intellectual disability	Escuela de Medicina y Ciencias de la Salud, Universidad del Rosario, Bogotá, Colombia	2 Subjects	Colombian	Case study	Intellectual disability	Overgrowth, kyphoscoliosis and facial dysmorphism	Communicating	WES	NGS, sanger sequencing	AR	15q22.31 (c.G2625A, p.W875X and c.G13559A, p.G4520E in HERC1)
Poduri et al., 2012 [271]	Somatic activation of AKT3 causes hemispheric developmental brain malformations	Children's Hospital Boston, 300 Longwood Avenue, Boston, MA 02115, USA	8 Subjects, Controls used	-	Case series	Intellectual disability and severe, intractable epilepsy		-	TGS	CNV; SNP; Karyotyping	De novo	1q43-q44 (c.G49A, p.E17K in AKT3)
Riviere et al., 2012 [272]	De novo germline and postzygotic mutations in AKT3, PIK3R2 and PIK3CA cause a spectrum of related megalencephaly syndromes	Seattle Children's Hospital, Seattle, Washington, USA	52 Subjects, 95 Controls	European	Case series	Megalocephaly, variable cortical malformation	Growth dysregulation with variable asymmetry, developmental vascular anomalies, distal limb malformations (syndactyly and polydactyly), and a mild connective tissue dysplasia	-	TES	Sanger sequencing; REF; targeted deep sequencing	De novo	1q43-q44 (c.C1393T, p.R465W and c.A686G, p.N229S in AKT3); 19p13.11 (c.G1117A; p.G373R in PIK3R2); 3q26.32 (PIK3CA mutations: c.G241A, p.E81K; c.G263A, p.R88Q; c.G1090A, p.G364R; c.G1093A, p.E365K; c.G1133A, p.C378Y; c.1359_1361del, p.E453del; c.G1633A, p.E545K; c.G2176A, p.E726K; c.G2740A, p.G914R; c.A3062G, p.Y1021C; c.A3073G, p.T1025A; c.C3104T, p.A1035V; c.G3129T, p.M1043I; c.C3139T, p.H1047Y; c.G3145A, p.G1049S)
Sameshima et al., 2019 [273]	MPPH syndrome with aortic coarctation and macrosomia due to CCND2 mutations	Hyogo Prefectural Awaji Medical Center, Sumoto, Hyogo, Japan	1 Subject, 2 Parents	Japanese	Case study	Polymicrogyria, seizures	Forehead protrusion, sacral cusp depression, low auricle, depressed nasal bridge and postaxial polydactyly, aortic coarctation	-	TGS	NGS, sanger sequencing	-	12p13.32 (c.C842G, p.P281R in CCND2)
Szalai et al., 2020 [274]	Maternal mosaicism underlies the inheritance of a rare germline AKT3 variant which is responsible for megalencephaly-polymicrogyria-polydactyly-hydrocephalus syndrome in two Roma half-siblings	University of Pecs, Medical School, Department of Medical Genetics, Pecs, Hungary	2 Subjects	Hungarian Roma	Case study	Intellectual disability, epilepsy, brain malformations, and megalencephaly	Dysmorphic features, visual impairment	-	WES, cytogenetics	Karyotyping; aCGH; sanger sequencing	Maternal mosaicism	1q43-q44 (c.C1393T, p.R465W in AKT3)
Tapper et al., 2014 [275]	Megalencephaly syndromes: exome pipeline strategies for detecting low-level mosaic mutations	University of Southampton, Southampton, Hampshire, United Kingdom; Wessex Regional Genetics Laboratory, Salisbury District Hospital, Salisbury, Wiltshire, United Kingdom	3 Subjects, 4 Parents	-	Case series	Macrocephaly, dysmorphic cerebellum, hypotonia	Capillary malformations, overgrowth and asymmetry, developmental delay	-	WES, cytogenetics	aCGH; sanger sequencing	-	3q26.32 (c.G2176A, p.E726K in PIK3CA); 19p13.11 (c.G1117A, p.G373R in PIK3R2)
Tenorio et al., 2014 [276]	A new overgrowth syndrome is due to mutations in RNF125	Hospital Universitario La Paz, Universidad Autónoma de Madrid (UAM), Madrid, Spain	6 Subjects, 350 Control	Spanish	Case series	Macrocephaly, intellectual disability	Overgrowth, hypoglycemia, inflammatory diseases resembling sjögren syndrome	-	TGS, cytogenetics	Karyotyping; aCGH; SNP array; MLPA; high-resolution melting; sanger sequencing; pyrosequencing	De novo	18q12.1 (RNF125 mutations: c.G336A, p.M112I; c.C488T, p.S163L; c.C520T, p.R174C)
Terrone et al., 2016 [277]	De novo PIK3R2 variant causes polymicrogyria, corpus callosum hyperplasia and focal cortical dysplasia	Federico II University, Naples, Italy	1 Subject	Italian	Case study	Left spastic hemiplegia, megalencephaly, perisylvian polymicrogyria, and mega corpus callosum	Synophrys, depressed nasal bridge, anteverted nares, pectus excavatum, broad thumb and hallux	-	WES	Sanger sequencing	De novo	19p13.11 (c.G1669C, p.D557H in exon 13 of PIK3R2)
Zarate et al., 2019 [278]	Constitutive activation of the PI3K-AKT pathway and cardiovascular abnormalities in an individual with Kosaki overgrowth syndrome	University of Arkansas for Medical Sciences, Little Rock, Arkansas	1 Subject, 1 Control	-	Case study	Dandy-Walker malformation, cervical spine arachnoid cyst, progressive scoliosis, white matter lesions, spastic diplegia	Craniofacial dysmorphism, hyperextensible skin, cardiac saccular aneurysms, developmental delay, low-frequency hearing loss	Obstructive	TES	Exome sequencing trio analysis, sanger sequencing	De novo	5q32 (c.T1696C, p.W566R in exon 12 of PDGFRB)

Array comparative genomic hybridization (aCGH). Autosomal Recessive (AR). Copy number variant (CNV). Multiplex ligation dependent probe amplification (MLPA). Next generation sequencing (NGS). Restriction endonuclease fingerprinting (REF). Single-molecule molecular inversion probes (smMIP). Single nucleotide polymorphisms (SNP). Targeted exome sequencing (TES). Targeted genome sequencing (TGS). Whole exome sequencing (WES). Whole genome sequencing (WGS)

### Vesicle regulation & cell adhesion

Table 7 details mutations in genes responsible for vesicle regulation and cell adhesion that contribute to the development of HC. Missense mutations were found in and glial fibrillary acidic protein (*GFAP*). Sorting nexin 10 (*SNX10*) displayed a nonsense mutation and clathrin heavy chain (*CLTC*) displayed a frameshift mutation. Additional mutations include ArfGAP with FG repeats 1 (*RAB*), multiple PDZ domain crumbs cell polarity complex component (*MPDZ*), beta 1,3-glucosyltransferase (*B3GALTL*), SEC24 homolog D, COPII coat complex component (*SEC24D*), and actin beta (*ACTB*).

**Table 7:** Vesicle regulation and cell adhesion

Citation	Title	Author affiliation	Case #	Ancestry	Study design	CNS phenotype	Non-CNS phenotype	Type of hydrocephalus	Genetic methodology	Genetic analysis	Inheritance	Genetic findings
Al-Dosari et al., 2013 [279]	Mutation in MPDZ causes severe congenital hydrocephalus	King Faisal Specialist Hospital and Research Center, Riyadh, Saudi Arabia	1 Subject, 50 Controls	Saudi	Case series	Callosal agenesis, hypotonia	Chorioretinal coloboma, atrial septal defect	Communicating	TGS, genotyping	Autozygosity mapping, linkage analysis, sanger sequencing	AR	9p23 (MPDZ)
Al-Jezawi et al., 2018 [280]	Compound heterozygous variants in the multiple PDZ domain protein (MPDZ) cause a case of mild non-progressive communicating hydrocephalus	College of Medicine and Heath Sciences, United Arab Emirates University	1 Subject, 2 Parents, 100 Controls	United Arab Emirates	Case study	Isolated hydrocephalus	Large head with frontal bossing and high arched palate	Communicating	WES	Variant analysis, sanger sequencing	AR	9p23 (MPDZ)
DeMari et al., 2016 [62]	CLTC as a clinically novel gene associated with multiple malformations and developmental delay	SUNY Upstate Medical University, Syracuse, New York	1 Subject, 2 Parents	Caucasian	Case study	Hypotonia	Prominent jaw, large anterior fontanel, bilateral hip laxity, and jaundice, low-set ears, depressed nasal bridge, anteverted nares, widely set involuted nipples	Communicating	WGS, cytogenetics	Karyotype, SNP microarray, co-segregation analysis, sanger sequencing	De novo	17q23.1 (A heterozygous de novo frameshift mutation, c.2737_2738dupGA p.D913Efs*59)
Mégarbané et al., 2013 [281]	Homozygous stop mutation in the SNX10 gene in a consanguineous Iraqi boy with osteopetrosis and corpus callosum hypoplasia	Unité de Génétique Médicale et laboratoire associé INSERM à l'Unité UMR_S 910, Pôle Technologie Santé, Université Saint-Joseph, Beirut, Lebanon	1 Subject, 1 Control	Iraqi	Case study	Macrocephaly, Brain atrophy, thin corpus callosum	Proptosis of the eyes, skeletal abnormality, strabismus, splenomegaly and joint hyperlaxity	Communicating	TGS	Direct sequencing	AR	7p15.2 (SNX10 gene)
Rajadhyax et al., 2007 [63]	Neurological presentation of Griscelli syndrome: obstructive hydrocephalus without haematological abnormalities or organomegaly	Genetics and Neurosurgery, Leeds General Infirmary, UK	1 Subject	Asian	Case study	Sixth nerve palsy, increased muscle tone	Patchy hyperpigmentation on the lower limbs, hemophagocytic lymphohistiocytosis	Obstructive	TGS	-	AR	2q36.3 (RAB27A)
Reis et al., 2008 [282]	Mutation analysis of B3GALTL in Peters Plus syndrome	Medical College of Wisconsin and Children's Hospital of Wisconsin, Milwaukee, Wisconsin, USA	8 Subjects, 180 Controls	Dutch	Case series	Intellectual disability	Central corneal opacity, defects in the posterior layers of the cornea, and lenticulo-corneal and/or irido-corneal adhesions, short stature, short broad hands with fifth finger clinodactyly, distinctive facial features, cleft lip and/or cleft palate, hearing loss, abnormal ears, heart defects, genitourinary anomalies	Communicating	TGS	Direct sequencing,	AR	13q12.3 (beta1,3-glucosyltransferase gene (B3GALTL))
Rodriguez et al., 2001 [283]	Infantile Alexander disease: spectrum of GFAP mutations and genotype–phenotype correlation	Laboratoire de Neurogénétique Moléculaire, INSERM U546, Université Paris VI, France	15 Subjects, 50 Controls	-	Case series	Macrocephaly, psychomotor regression, seizures, and spasticity	Respiratory difficulties	Communicating	TES	-	De novo	17q21.31 (Missense, heterozygous, de novo GFAP mutations (R79H; four had R239C; and one had R239H))
Sakakibara et al., 2007 [284]	A case of infantile Alexander disease diagnosed by magnetic resonance imaging and genetic analysis	Nara Medical University, Japan	1 Subject	-	Case study	Megalencephalic, seizures, white matter abnormalities	Bulbar paralysis	Obstructive	TGS	-	AD	17q21.31 (R239H mutation of glial fibrillary acidic protein(GFAP))
Saugier-Veber et al., 2017 [285]	Hydrocephalus due to multiple ependymal malformations is caused by mutations in the MPDZ gene	Normandie Univ, UNIROUEN, INSERM U1245, Normandy Centre for Genomic and Personalized Medicine, Rouen University Hospital, F76000, Rouen, France	5 Subjects, 3 Controls	-	Case series	Multifocal ependymal malformations		Obstructive	TGS, cytogenetics	Karyotyping, variant analysis, sanger sequencing, targeted NGS	AR	9p23 (MPDZ gene)
Takeyari et al., 2018 [286]	Japanese patient with Cole-carpenter syndrome with compound heterozygous variants of SEC24D	Osaka University Graduate School of Medicine, Osaka, Japan	1 Subject	Japanese	Case study	Craniosynostosis	Prominent eye and micrognathia, short neck, scoliosis, and chest deformity, bone fractures, wormian bones, lordosis, and long thin bones	-	TES	Variant analysis, sanger sequencing	-	4q26 (SEC24D)
Van der Knaap et al., 2005 [287]	Unusual variants of Alexander's disease	VU University Medical Center, De Boelelaan 1117, 1081 HV Amsterdam, the Netherlands	10 Subjects, 100 Controls	-	Case series	Cerebral white matter abnormalities, brainstem lesions	Scoliosis, dysphagia, gait disturbances	Obstructive	TGS	-	De novo	17q21.31 (GFAP)
Zhang et al., 2020 [288]	Prenatal presentation and diagnosis of Baraitser-Winter syndrome using exome sequencing	Virginia Tech Carilion School of Medicine, Roanoke, Virginia, USA	1 Subject, 2 Parents		Case study	Interhemispheric cyst	Cystic hygroma and omphalocele, ocular coloboma, hypertelorism, heart, renal, musculoskeletal system defects	-	TGS	NGS, variant analysis,	AD	7p22.1 (ACTB)

Array comparative genomic hybridization (aCGH). Autosomal Dominant (AD). Autosomal Recessive (AR). Next generation sequencing (NGS). Single nucleotide polymorphisms (SNP). Targeted exome sequencing (TES). Targeted genome sequencing (TGS). Whole exome sequencing (WES). Whole genome sequencing (WGS)

GFAP is required for white-matter architectural development and myelination, perhaps accounting for the neurodevelopmental comorbidities frequently observed in patients with HC [59]. Mutations in the phosphoinositide binding domain of *SNX10* alters endosomal integrity, suggesting a potential pathogenic mechanism in vesicular trafficking [60]. Additionally, mutations in this gene locus can disrupt interactions between sorting nexins and the V-ATPase complex further contributing to vesicle dysfunction and ciliopathy [61]. *CLTC* contributes to the development of the vesicular coat, and a mutation within this gene locus may disrupt vesicle stability [62]. Mutations in *RAB27A* are associated with Griscelli syndrome, characterized by albinism, hematological abnormalities, and organ malformation which can also present with HC [63]. *SEC24* is also involved in intracellular trafficking by interacting with export signals from the endoplasmic reticulum and regulating cargo transport [64]. In addition, *MPDZ* is highly expressed in tight junctions suggesting that a mutation within this gene locus may disrupt alter tissue permeability [65]. Finally, *B3GALTL* interacts with the thrombospondin type 1 repeat (TSR) protein family which play varied roles in maintain and regulating cell–cell adhesion [66].

### Glycosylation defects

Table 8 summarizes genes implicated in human HC associated with defects in glycosylation. Nonsense mutations were seen in protein O-mannose kinase (*POMK*), and protein O-mannosyltransferase 1 (*POMT1*). Loss of function mutations were identified in dystroglycan 1 (*DAG1*) and isoprenoid synthase domain containing gene (*ISPD*). Additional mutations included those in protein C, inactivator of coagulation factors Va and VIIIa (*PROC*), fukutin related protein (*FKRP*), protein O-mannosyltransferase 2 (*POMT2*), protein O-linked mannose N-acetylglucosaminyltransferase 1 (beta 1,2-) (*POMGNT1*), LARGE xylosyl and glucuronyltransferase 1 (*LARGE1*) and a translocation between chromosome 5 and 6, t(5;6) (q35;q21). In addition, *DAG1* codes for dystroglycan, a protein involved in extracellular matrix integrity and the genetic etiology of many neurological syndromes. Mutations in *DAG1* have been found to contribute to Walker-Warburg syndrome and other muscular dystrophy-dystroglycanopathies which can be associated with HC [67]. Dystroglycan may also be affected through defects in its glycosylation patterns. For instance, mutations in *POMK* have been shown to impair the glycosylation of a-dystroglycan affecting cytoskeleton stability [68]. Other genes contributing to dystroglycanopathies through glycosylation errors include *POMT1*, *POMT2*, *POMGNT1*, *FKRP*, *ISPD* and *LARGE1* [69].

**Table 8:** Glycosylation defects

Citation	Title	Author affiliation	Case #	Ancestry	Study design	CNS phenotype	Non-CNS phenotype	Type of hydrocephalus	Genetic methodology	Genetic analysis	Inheritance	Genetic findings
Beltran-Valero de Bernabé et al., 2004 [289]	Mutations in the FKRP gene can cause muscle-eye-brain disease and Walker-Warburg syndrome	University Medical Centre Nijmegen, Nijmegen, The Netherlands	2 Patients, 200 Controls	German, Asian	Case series	Dandy walker-like malformation, intellectual disability	Muscular dystrophy, left ventricular hypertrophy, retinal and eye developmental issues	Communicating	TES	Direct sequencing, linkage analysis	-	19q13.32 (FKRP)
Beltrán-Valero de Bernabé et al., 2002 [290]	Mutations in the O-mannosyltransferase gene POMT1 give rise to the severe neuronal migration disorder Walker-Warburg syndrome	University Medical Centre Nijmegen, Nijmegen, The Netherlands	30 Subjects, 105 Controls	Turkish, Italy, Dutch, Australian	Case series	Cobblestone lissencephaly, occipital encephalocele	Eye malformations, congenital muscular dystrophy or elevated creatine kinase	Obstructive	TES	Linkage analysis, SSCP, restriction enzyme analysis	AR	9q34.13 (POMT1)
Biancheri et al., 2006 [291]	POMGnT1 mutations in congenital muscular dystrophy: genotype–phenotype correlation and expanded clinical spectrum	University of Genova, Italy	3 Subjects, 192 Controls	Italian	Case series	Intellectual disability, epilepsy, and lissencephaly	Congenital muscular dystrophy, ocular abnormalities	Communicating	TGS	Direct sequencing	AR	1p34.1 (POMGnT1)
Bouchet et al., 2007 [292]	Molecular heterogeneity in fetal forms of type II lissencephaly	Bichat-Claude Bernard Hospital, Biochimie Métabolique, Paris, France	47 Subjects, 100 Controls	French	Case series	Agyria, thick leptomeninges, disorganized cortical ribbon, cerebellar dysplasia		Communicating	TGS	-	AR	9q34.13 (15 in POMT1); 14q24.3 (five in POMT2); 1p34.1 (POMGNT1)
Cormand et al., 2001 [293]	Clinical and genetic distinction between Walker-Warburg syndrome and muscle-eye-brain disease	University of Helsinki, Finland	29 Subjects	Turkish, Netherlands, German, Pakistani, Swedish, Palestinian, Dutch, and American	Case series	Malformation of neuronal migration compatible with cobblestone complex	Elevated serum creatine kinase level or abnormal muscle biopsy, and ocular abnormalities	-	Genotyping	Linkage analysis	AR	MEB gene locus localized to 1p32-p34
Currier et al., 2005 [294]	Mutations in POMT1 are found in a minority of patients with Walker-Warburg syndrome	Beth Israel Deaconess Medical Center, Boston, Massachusetts, USA	30 Subjects, 110 Controls	Asian, African, and Caucasian	Case series	Cerebellar hypoplasia, brainstem hypoplasia, agenesis of the corpus callosum, agenesis of the septum pellucidum, interhemispheric fusion, and the presence of an encephalocele	Ocular abnormalities, congenital muscular dystrophy	Obstructive	TES	Microsatellite marker assay	AR	9q34.13 (POMT1)
Geis et al., 2019 [295]	Clinical long-time course, novel mutations and genotype–phenotype correlation in a cohort of 27 families with POMT1-related disorders	Klinik St. Hedwig, University Children's Hospital Regensburg (KUNO), Steinmetzstr. 1–3, 93,049, Regensburg, Germany	35 Subjects	German, Turkish, Indonesian, Gipsy, African	Case series	Lissencephaly type II, hypoplasia of the pons and/or brainstem, cerebellar hypoplasia, hypoplasia of the corpus callosum, encephalocele	Muscle weakness, muscular dystrophy, GI malformations	Communicating	TGS	Direct sequencing, sanger sequencing, massive parallel sequencing	AR	9q34.13 (POMT1)
Godfrey et al., 2007 [69]	Refining genotype phenotype correlations in muscular dystrophies with defective glycosylation of dystroglycan	Hammersmith Hospital, Imperial College, London, UK	92 Subjects	Australia, Turkey	Case series	Cobblestone lissencephaly	Limb girdle muscular dystrophy, congenital muscular dystrophy, elevated serum ck	Communicating	TGS	Unidirectional sequencing, HA, segregation analysis,	AR, De novo	9q34.13 (POMT1); 14q24.3 (POMT2); 1p34.1 (POMGnT1); 9q31.2 (FKTN); and 22q12.3 (LARGE)
Hehr et al., 2007 [296]	Novel POMGnT1 mutations define broader phenotypic spectrum of muscle-eye-brain disease	University of Regensburg, Universitätklinikum D3, Franz-Josef-Strauss-Allee 11, Regensburg, Germany	9 Subjects	German, Turkish, English	Case series	Global developmental delay, seizures, cerebellar cysts, intellectual disability	Congenital muscular dystrophy, se- vere congenital myopia, glaucoma, retinal hypoplasia	Communicating	TGS	Cycle sequencing, linkage analysis, restriction enzyme analysis	AR	1p34.1 (POMGnT1)
Ichiyama et al., 2016 [297]	Fetal hydrocephalus and neonatal stroke as the first presentation of protein C deficiency	Kyushu University, Fukuoka, Japan	1 Subject	Asian	Case study	Isolated hydrocephalus	Slight developmental delay	-	TES	Direct sequencing	-	2q14.3 (PROC c.574_576delAAG)
Kano et al., 2002 [298]	Deficiency of alpha-dystroglycan in muscle-eye-brain disease	Osaka University Graduate School of Medicine, 2–2 B9, Yamadaoka, Suita, Osaka, Japan	3 Subjects, 1 Control	Turkish, French	Case series	Type II lissencephaly, Intellectual disability	Congenital muscular dystrophy, congenital myopia, congenital glaucoma, pallor of the optic discs, retinal hypoplasia, hydrocephalus, myoclonic jerks	Communicating	TGS	-	AR	1p34.1 (POMGnT1)
Karadeniz et al., 2002 [299]	De novo translocation t(5;6)(q35;q21) in an infant with Walker-Warburg syndrome	Burak Woman's Hospital, Department of Medical Genetics, Ankara, Turkey	1 Subject, 2 Parents	-	Case study	Hypoplasia of cerebellar vermis, enlargement of cisterna magna, bilateral dilatation of lateral ventricles, widespread agyria, and irregularity of the white matter-gray matter line	Eye abnormalities with microphthalmia cataract, congenital muscular dystrophy	Communicating	-	G-banding	De novo	translocation t(5;6)(q35;q21)
Preiksaitiene et al., 2020 [300]	Pathogenic homozygous variant in POMK gene is the cause of prenatally detected severe ventriculomegaly in two Lithuanian families	Vilnius University, Vilnius, Lithuania	4 Subjects, 98 Controls	Lithuanian	Case series	Isolated hydrocephalus	Highly variable	Dependent on phenotype of dystroglycanopathy	WES	Sanger sequencing	De novo*,* AR	8p11.21 (homozygous nonsense variant in the POMK)
Van Reeuwijk et al., 2005 [301]	POMT2 mutations cause alpha-dystroglycan hypoglycosylation and Walker-Warburg syndrome	Radboud University Nijmegen Medical Centre, Nijmegen, The Netherlands	3 Subjects, Controls used	Moroccan, Pakistani, Bengali	Case series	Lissencephaly, agenesis of the corpus callosum, fusion of the hemispheres, cerebellar hypoplasia, and neuronal overmigration	Eye malformations (cataract, microphthalmia, buphthalmos, and peters anomaly)	-	TGS	Homozygosity mapping, direct sequencing	AR	14q24.3 (POMT2)
Van Reeuwijk et al., 2006 [302]	The expanding phenotype of POMT1 mutations: from Walker-Warburg syndrome to congenital muscular dystrophy, microcephaly, and mental retardation	Radboud University Nijmegen Medical Centre, Nijmegen, The Netherlands	28 Subjects, 100 Controls	Italy, Netherlands, Pakistan, Lebanon, India, Qatar, Ireland, turkey	Case series	Lissencephaly, agenesis of the corpus callosum, fusion of the hemispheres, cerebellar hypoplasia, and neuronal overmigration	Myopia, gait disturbances	Communicating	TGS	Linkage analysis	-	9q34.13 (POMT1)
Van Reeuwijk et al., 2010 [303]	A homozygous FKRP start codon mutation is associated with Walker-Warburg syndrome, the severe end of the clinical spectrum	Radboud University Nijmegen Medical Centre, Nijmegen, The Netherlands	2 Subjects, 2 Parents	Caucasian	Case series	Lissencephaly, agenesis of the corpus callosum, fusion of the hemispheres, cerebellar hypoplasia, and neuronal overmigration	Cataracts, muscular dystrophy	Communicating	TGS	SNP	-	19q13.32 (FKRP)
Riemersma et al., 2015 [304]	Absence of α- and β-dystroglycan is associated with Walker-Warburg syndrome	Leiden University Medical Center, the Netherlands, Sydney Children's Hospital, University of New South Wales, Sydney, Australia, Rambam Health Care Campus, Haifa, Weizmann Institute of Science, Rehovot, Israel	5 Subjects, Controls used	Israeli-Arab	Case series	Hypotonia, posterior fossa, a small midline encephalocele, a hypoplastic vermis, intracranial calcifications	Elevated ck, elevated lfts, respiratory failure, bilateral corneal opacities, and glaucoma	Communicating	TES	Homozygosity mapping, CNV, sanger sequencing	-	3p21.31 (homozygous loss-of-function frameshift mutation in the DAG1 gene)
Saredi et al., 2012 [305]	Novel POMGNT1 point mutations and intragenic rearrangements associated with muscle-eye-brain disease	Foundation Neurological Institute C. Besta, Milano, Italy	3 Subjects, 1 Control	Italian	Case series	Microcephaly, spastic tetraparesis	Rounded forehead, thin lips, short neck, micrognathia, motor disability, eye abnormalitie	Communicating	TGS	Cycle sequencing, MLPA	AR	1p34.1 (c.643C > T, c.1863delC in POMGnT1)
Vervoort et al., 2004 [306]	POMGnT1 gene alterations in a family with neurological abnormalities	J. C. Self Research Institute of Human Genetics, Greenwood Genetic Center, Greenwood, SC, USA	2 Subjects, 2 Parents, 500 Controls	Caucasian	Case series	Hypotonia, bilateral frontal polymicrogyria, abnormal cerebellum, and characteristic flattened dystrophic pons	Congenital muscular dystrophy, congenital glaucoma and severe myopia	-	TGS	Haplotype analysis, SSCP, cycle sequencing	AR	1p34.1 (POMGnT1)
Willer et al., 2012 [307]	ISPD loss-of-function mutations disrupt dystroglycan O-mannosylation and cause Walker-Warburg syndrome	University of Iowa Roy J and Lucille A Carver College of Medicine, Iowa City, Iowa, USA	7 Subjects, Controls used	-	Case series	Cobblestone lissencephaly, severe brainstem hypoplasia with a kink at the isthmus and severe hypoplasia of the cerebellum	Muscular dystrophy, bilateral microphthalmia with cataracts and arrested retinal development	Communicating	TGS, cytogenetics	Linkage analysis, targeted NGS, aCGH, CNV, Sanger sequencing	AR	7p21 (ISPD mutation)
Yis et al., 2007 [308]	A case of Walker-Warburg syndrome resulting from a homozygous POMT1 mutation	University of Dokuz Eylul, 35,340 Izmir, Turkey	1 Subject, 2 Parents	-	Case study	Type II lissencephaly and pontocerebellar hypoplasia	Severe ocular malformations and congenital muscular dystrophy	Communicating	TGS	Linkage analysis, direct sequencing	AR	9q34.13 (mutation (R514X) in the POMT1 gene)
Yoshida et al., 2001 [309]	Muscular dystrophy and neuronal migration disorder caused by mutations in a glycosyltransferase, POMGnT1	Central Laboratories for Key Technology, Kirin Brewery Co., Ltd., Kanazawa-ku, Yokohama, Japan	6 Subjects	Turkish, French	Case series	Lissencephaly	Congenital muscular dystrophy, ocular abnormalities	Communicating	TGS	Direct sequencing	AR	1p34.1 (POMGnT1)

Array comparative genomic hybridization (aCGH). Autosomal Recessive (AR). Copy number variant (CNV). Heteroduplex analysis (HA). Multiplex ligation dependent probe amplification (MLPA). Polymerase chain reaction (PCR). Next generation sequencing (NGS). Single nucleotide polymorphisms (SNP). Single-strand conformation polymorphisms (SSCP). Targeted exome sequencing (TES). Targeted genome sequencing (TGS). Whole exome sequencing (WES). Whole genome sequencing (WGS)

### Growth factor related signaling

Table 9 summarizes genetic mutations associated with growth factor related signaling dysfunction. Mutations were observed in fibroblast growth factor receptor 1 (*FGFR1*), fibroblast growth factor receptor 2 (*FGFR2*), fibroblast growth factor receptor 3 (*FGFR3*), ZPR1 zinc finger (*ZPR1*), and fibrillin 1 (*FBN1*). Specifically, exon 7 displayed a missense mutation in *FGFR2* and exon 64 displayed a mutation in *FBN1*. Mutations in *FGFR* play pleiotropic roles in numerous syndromes including Crouzon syndrome, Jackson-Weiss syndrome, Apert syndrome and Pfeiffer syndrome [70–73]. These craniosynostoses have been associated with HC and *FGFR* mutations contributing to bony abnormalities, which may explain the venous and CSF outflow obstructions leading to this phenotype [74]. The FGFR mutations identified are predominantly gain of function mutations altering ligand binding and tyrosine kinase activity [75]. In addition, *ZPR1* contributes to cell proliferation and *FBN1* is associated with TGF beta signaling suggesting their mechanistic contributions to the HC phenotype seen in patients with these phenotypes [76].

**Table 9:** Growth factor signaling

Citation	Title	Author affiliation	Case #	Ancestry	Study design	CNS phenotype	Non-CNS phenotype	Type of hydrocephalus	Genetic methodology	Genetic analysis	Inheritance	Genetic findings
Abdel-Salam et al., 2011 [310]	Muenke syndrome with pigmentary disorder and probable hemimegalencephaly: An expansion of the phenotype	National Research Centre, Cairo, Egypt	1 Patient, 2 parents	-	Case study	Left HME, inadequate differentiation of white and gray matter, underdeveloped corpus callosum, abnormal hippocampus configuration, right coronal, sagittal, and lambdoid suture synostoses	Frontal bossing, sparse, hypopigmented, curly hair, prominent eyes, low-set ears, hypoplastic maxilla, long philtrum, brachydactyly with fusiform fingers, skin hyperpigmentation	Obstructive	TES	MLPA, DHPLC	AD	4p16.3 (FGFR3 showed a c.749C > G, p.Pro250Arg substitution)
Arnaud-López et al., 2007 [311]	Crouzon with acanthosis nigricans. Further delineation of the syndrome	Instituto Mexicano del Seguro Social, Guadalajara, México	2 Subjects	-	Case series	Craniosynostosis	Laryngomalacia, acanthosis nigricans, choanal stenosis, double collecting system and dysplastic kidney	Communicating	TGS, cytogenetics	Karyotyping	AD	4p16.3 (FGFR3)
Chen et al., 2001 [312]	Prenatal diagnosis and genetic analysis of type I and type II thanatophoric dysplasia	Mackay Memorial Hospital, Taipei, Taiwan	4 Subjects, control matched sampling	Chinese	Case series	Cloverleaf skull, macrocephaly, synostosis	Short-limbed dwarfism, multiple skeletal dysplasias	-	TES	Direct sequencing	-	4p16.3 (FGFR3)
Chen et al., 2008 [313]	Craniosynostosis and congenital tracheal anomalies in an infant with Pfeiffer syndrome carrying the W290C FGFR2 mutation	Mackay Memorial Hospital, Taipei, Taiwan	1 Subject	-	Case study	Dandy walker—cerebellar malformations	Turricephalic prominent forehead, hypertelorism, low-set ears, a flat nasal bridge, mid-face hypoplasia, bilateral cleft lip and palate, a thick nuchal fold, and a distended abdomen, and multicystic kidneys	Communicating	-	-	De novo	10q26.13 (c.870 G > T (TGG > TGT) in the FGFR2)
Chen et al., 2017 [314]	Pfeiffer syndrome with FGFR2 C342R mutation presenting extreme proptosis, craniosynostosis, hearing loss, ventriculomegaly, broad great toes and thumbs, maxillary hypoplasia, and laryngomalacia	Mackay Memorial Hospital, Taipei, Taiwan	1 Subject	-	Case study	Multisynostoses of sagittal and coronal sutures	Bilateral hearing loss, brachycephaly, hypertelorism, broad big toes and thumbs, low-set ears, laryngeomalacia and midface hypoplasia	Obstructive	Cytogenetics	Karyotyping	AD	10q26.13 (FGFR2 C342R mutation)
Fonseca et al., 2008 [315]	Second case of Beare-Stevenson syndrome with an FGFR2 Ser372Cys mutation	Universidade Federal do Rio de Janeiro, Rio de Janeiro, Brazil.	1 Subject	-	Case study	Craniosynostosis, crouzonoid-like features, and cloverleaf skull	Cutis gyrata, acanthosis nigricans, skin furrows, skin tags, anogenital anomalies, and prominent umbilical stump	Communicating	TES	Direct sequencing	AD	10q26.13 (FGFR2 Ser372Cys mutation.)
González-Del Angel et al., 2016 [316]	Expansion of the variable expression of Muenke syndrome: Hydrocephalus without craniosynostosis	Instituto Nacional de Pediatría, Mexico City, Mexico	56 Subjects	Mexican	Case series	Uni- or bicoronal craniosynostosi	Wide variability	Obstructive	TES	Direct sequencing, restriction enzyme analysis	AD	4p16.3 (FGFR3)
Gripp et al., 1998 [317]	Phenotype of the fibroblast growth factor receptor 2 Ser351Cys mutation: Pfeiffer syndrome type III	The Children's Hospital of Philadelphia, Pennsylvania, USA	1 Subject	Caucasian	Case study	Seizures, developmental delay, pansynostosis	Bilateral elbow ankylosis, radial head dislocation, Extreme proptosis with luxation of the eyes out of the lids, apnea and airway obstruction, intestinal non-rotation	Communicating	TES	SSCP, cycle sequencing	AD	10q26.13 (Ser351Cys in FGFR2)
Gupta et al., 2020 [318]	Crouzon Syndrome in a Ten-week-old Infant: A Case Report	All India Institute of Medical Sciences, Patna, Bihar, India	1 Subject	Japanese	Case study	Neurologic and neuromuscular impairment, Craniosynostosis	Airway obstruction, craniofacial dysostosis with abnormal shape of the skull, proptosis, hypertelorism, curved nose and frontal bossing	Communicating	-	-	AR	10q26.13 (FGFR2)
Ito et al., 2018 [76]	A ZPR1 mutation is associated with a novel syndrome of growth restriction, distinct craniofacial features, alopecia, and hypoplastic kidneys	University of Ottawa, Ottawa, Canada	4 Subjects, 3 Controls	New Mexican Hispanic heritage	Case series	Microcephaly	Growth restriction, distinctive craniofacial features, congenital alopecia, hypoplastic kidneys with renal insufficiency, global developmental delay, severe congenital sensorineural hearing loss, and genital hypoplasia	-	WES	Sanger sequencing	AR	11q23.3 (ZPR1 Zinc Finger)
Kan et al., 2002 [75]	Genomic screening of fibroblast growth-factor receptor 2 reveals a wide spectrum of mutations in patients with syndromic craniosynostosis	The John Radcliffe Hospital, Oxford, United Kingdom	259 Subjects, 128 Controls	-	Case series	Cloverleaf skull, craniosynostosis		Communicating	TES	HA	AD	10q26.13 (FGFR2)
Lajeunie et al., 2006 [319]	Mutation screening in patients with syndromic craniosynostoses indicates that a limited number of recurrent FGFR2 mutations accounts for severe forms of Pfeiffer syndrome	Hôpital Necker-Enfants malades, Paris, France	129 Subjects, 65 Controls	-	Case series	Synostosis of one or several cranial sutures	Ocular proptosis, maxillary hypoplasia and midface retrusion	Communicating	TES	Direct sequencing	AD	8p11.23 (FGFR 1); 10q26.13 (FGFR2); 4p16.3 (FGFR 3 mutation)
Priolo et al., 2000 [320]	Pfeiffer syndrome type 2 associated with a single amino acid deletion in the FGFR2 gene	G. Gaslini Institute, Genova, Italy	1 Subject, 60 Controls	-	Case study	Acrocephalo-trygonocephaly with cloverleaf skull, callosal dysgenesis and Chiari I malformation	Facial dysmorphism, radial clinodactyly of the thumbs and valgus deviation of the halluces	Unclear	TGS	Cycle sequencing	AD	10q26.13 (FGFR2)
Przylepa et al., 1996 [321]	Fibroblast growth factor receptor 2 mutations in Beare-Stevenson cutis gyrata syndrome	The Johns Hopkins University School of Medicine, Baltimore, Maryland, USA	5 Subjects, 3 Parents, and 50 Controls	-	Case series	Craniosynostosis	Cutis gyrata, acanthosis nigricans, craniofacial dysmorphism, digital anomalies, umbilical and anogenital abnormalities	Communicating	TGS	HA, fluorescent dideoxy terminator method, restriction enzyme analysis	AD	10q26.13 (FGFR2)
Rump et al., 2006 [322]	Severe complications in a child with achondroplasia and two FGFR3 mutations on the same allele	University Medical Center Groningen, University of Groningen, The Netherlands	1 Subject, 2 Parents	Dutch	Case study	Megalencephalic	Midface hypoplasia, lordotic lumbar spine, trident hand configuration, achondroplasia, respiratory failure	Communicating	TGS	Variant analysis	AD	4p16.3 (p.G380R mutation of FGFR3)
Rutland et al., 1995 [323]	Identical mutations in the FGFR2 gene cause both Pfeiffer and Crouzon syndrome phenotypes	Institute of Child Health, London, UK	12 Subjects	-	Case series	Cloverleaf skull, craniosynostosis	Digital abnormalities	Communicating	TES	SSCP, direct sequencing, restriction endonuclease analysis	De novo	10q26.13 (FGFR2)
Schaefer et al., 1998 [324]	Novel mutation in the FGFR2 gene at the same codon as the Crouzon syndrome mutations in a severe Pfeiffer syndrome type 2 case	H.A. Chapman Research Institute of Medical Genetics, Tulsa, Oklahoma, USA	1 Subject	-	Case study	Cloverleaf skull	Proptosis, radioulnar synostosis and broad thumbs and great toes	Communicating	TES	Cycle sequencing	-	10q26.13 (G to T mutation in codon 290 exon 7 of the FGFR2)
Takenouchi et al., 2013 [325]	Severe congenital lipodystrophy and a progeroid appearance: Mutation in the penultimate exon of FBN1 causing a recognizable phenotype	Keio University School of Medicine, Tokyo, Japan	1 Subject	Japanese	Case study	Craniosynostosis	Progeroid appearance, wide-open anterior fontanelle, low-set ears, long arms and legs, arachnodactyly, and arthrogryposis, hydronephrosis	Communicating	TGS	NGS, sanger sequencing	AD	15q21.1 (exon 64 of the FBN1 gene)

Autosomal Dominant (AD). Autosomal Recessive (AR). Copy number variant (CNV). Denaturing high performance liquid chromatography (DHPLC). Heteroduplex analysis (HA). Multiplex ligation dependent probe amplification (MLPA). Next generation sequencing (NGS). Single-strand conformation polymorphisms (SSCP). Targeted exome sequencing (TES). Targeted genome sequencing (TGS). Whole exome sequencing (WES). Whole genome sequencing (WGS)

### Extracellular matrix defects

Table 10 highlights the genetic mutations contributing to extracellular matrix defects. Mutations were found in fukutin (*FKTN*), cartilage associated protein (*CRTAP*), collagen type VIII alpha 2 chain (*COL8A2*), collagen type III alpha 1 chain (*COL3A1*), collagen type IV alpha 1 chain (*COL4A1*), vascular cell adhesion molecule 1 (*VCAM1*), protein tyrosine phosphatase receptor type F (*PTPRF*), fibrillin 1 (*FBN1*), laminin subunit beta 1 (*LAMB1*), FRAS1 related extracellular matrix 1 (*FREM1*), and the plasminogen gene. *CRTAP* is involved in proline hydroxylation which ultimately contributes to collagen stability and functionality [77]. Mutations within the *CRTAP* gene locus can lead to Cole-Carpenter syndrome, which is associated with HC [77]. Other basement membrane proteins encoded by *COL8A2*, *COL3A1*, *COL4A1*, *VCAM1*, and *PTPRF* may alert the extracellular matrix and contribute to HC. For instance, a mutation in *COL3A1* affects its triple helix stability leading to degradation and further defects in the basement membrane [78]. *LAMB1* knockdown in zebrafish disrupted laminin integrity, a component of the basal lamina, leading to brain structural abnormalities [79], suggesting a potential pathogenic link to HC.

**Table 10:** Extracellular matrix defects

Citation	Title	Author affiliation	Case #	Ancestry	Study design	CNS phenotype	Non-CNS phenotype	Type of hydrocephalus	Genetic methodology	Genetic analysis	Inheritance	Genetic findings
Balasubramanian et al., 2015 [77]	CRTAP mutation in a patient with Cole-Carpenter syndrome	Sheffield Children's NHS Foundation Trust, UK	1 Subject	Asian Pakistan	Case subject	Thoraco-lumbar scoliosis and sutural craniosynostosis	Osteogenesis imperfecta, bilateral limb deformities, joint hypermobility, prominent eyes with a proptotic appearance, greyish blue sclerae, and dentinogenesis imperfecta	Communicating	TGS	Variant analysis	-	3p22.3 (c.118G > T mutation in exon 1 of the CRTAP gene)
Çiftçi et al., 2003 [326]	Ligneous conjunctivitis, hydrocephalus, hydrocele, and pulmonary involvement in a child with homozygous type I plasminogen deficiency	University of Ankara Medical School, 06100, Dikimevi Ankara, Turkey	1 Subject, 2 Parents, 1 Control	Turkish	Case study	Isolated hydrocephalus	Tracheal pseudomembranes, bilateral hydrocele and unilateral inguinal hernia	Obstructive	TGS	SSCP, direct sequencing	AR	6q26 (L650fsX652 mutation (deletion of 2081C))
Cormand et al., 1999 [327]	Assignment of the muscle-eye-brain disease gene to 1p32-p34 by linkage analysis and homozygosity mapping	University of Helsinki, Finland	12 Subjects, 27 Controls	Finnish, Turkish	Case series	Intellectual disability, polymicrogyria-pachygyria-type neuronal migration disorder of the brain	Ocular abnormalities, congenital muscular dystrophy	Communicating	Genotyping	Linkage analysis, haplotype analysis	AR	1p34.3 (COL8A2), 1p21.2 (VCAM1), 1p34.2 (PTPRF)
Cotarelo et al., 2008 [328]	Two new patients bearing mutations in the fukutin gene confirm the relevance of this gene in Walker-Warburg syndrome	Universidad Autónoma de Madrid, Madrid, Spain	2 Subjects, 3 Family Members	Ashkanazi Jewish, Spanish	Case series	Overriding cranial bones, monolobar holoprosencephaly, interhemispheric cyst, incomplete cleavage of the thalamus and corpora quadrigemina, an absent corpus callosum and rhombencephalic hypoplasia	Microphtalmia, atrial septal defect, double subaortic ventricular defect, hypoplastic left ventricle outlet, stenotic pulmonary valve and infundibular transposition of great vessels with no innominate vein, and retinal dysplasia	External and internal	TGS	Restriction endonuclease enzyme analysis, PCR	AR	9q31.2 (FKTN)
de Bernabé et al., 2003 [329]	A homozygous nonsense mutation in the fukutin gene causes a Walker-Warburg syndrome phenotype	University Medical Centre Nijmegen, Nijmegen, Netherlands	30 Subjects, 105 Controls	Japanese	Case series	Cobblestone lissencephaly with agenesis of the corpus callosum, fusion of hemispheres, hydrocephalus, dilatation of the fourth ventricle, cerebellar hypoplasia, hydrocephalus, and sometimes encephalocele	Eye malformations and congenital muscular dystrophy	Communicating	TGS	Linkage analysis, direct sequencing, SSCP	AR	9q31.2 (FKTN)
Horn et al., 2011 [330]	Progeroid facial features and lipodystrophy associated with a novel splice site mutation in the final intron of the FBN1 gene	Charité-Universitätsmedizin Berlin, Berlin, Germany	1 Subject, 150 Controls	German	Case study	Psychomotor delay, hypotonia	Triangular facial shape, large head with a broad and prominent forehead, deep set eyes with proptosis, downward slanting palpebral fissures, and a high nasal bridge, highly arched palate and mild retrognathia, generalized lipodystrophy, long fingers and toes, bilateral pes valgus	-	TGS, cytogenetics	Karyotyping, aCGH	AD	15q21.1 (FBN1)
Kondo-lida et al., 1999 [331]	Novel mutations and genotype–phenotype relationships in 107 families with Fukuyama-type congenital muscular dystrophy (FCMD)	Human Genome Center, Institute of Medical Science, University of Tokyo, Japan	19 Subjects, 50 Controls	Japanese	Case series	Intellectual delay micropolygyria, pachygyria and agyria	Congenital muscular dystrophy, eye abnormalities	Communicating	TGS	SSCP, direct sequencing	De novo	9q31, gene FCMD
Kroes et al., 2003 [78]	Ehlers-Danlos syndrome type IV: unusual congenital anomalies in a mother and son with a COL3A1 mutation and a normal collagen III protein profile	University Medical Center WKZ, Internal mail KC 04.084.2, Lundlaan 6, 3584 EA Utrecht, the Netherlands	2 Subjects	-	Case series	Macrocephaly	Blue sclerae, unilateral clubfoot, esophageal atresia, joint hyperlaxity	Communicating	TGS	-	-	2q32.2 (COL3A1)
Radmanesh et al., 2013 [332]	Mutations in LAMB1 cause cobblestone brain malformation without muscular or ocular abnormalities	University of California, San Diego, CA, USA	2 Subjects, 200 Controls	Egyptian and Turkish	Case series	Cortical gyral and white-matter signal abnormalities, severe cerebellar dysplasia, brainstem hypoplasia, and occipital encephalocele	Minor optic atrophy	Communicating	WES	Sanger sequencing	AR	7q31.1 (LAMB1)
Saito et al., 2000 [333]	Haplotype-phenotype correlation in Fukuyama congenital muscular dystrophy	Tokyo Women's Medical University, School of Medicine, Japan	56 Subjects, 82 Controls	Japanese	Case series	Cobblestone lissencephaly with cerebral and cerebellar cortical dysplasia	Congenital muscular dystrophy, eye abnormalities	Communicating	Allelotyping	Haplotype analysis, microsatellite marker assay	AR	FCMD gene
Schott et al., 1998 [334]	Therapy with a purified plasminogen concentrate in an infant with ligneous conjunctivitis and homozygous plasminogen deficiency	Klinikum Mannheim, University of Heidelberg, Germany	1 Subject, 1 Control, 2 Parents, 1 Brother	Turkish	Case study	Macrocephalus	Pseudomembranous conjunctivitis, ligneous conjunctivitis	-	TGS	SSCP, cycle sequencing, restriction enzyme analysis	AR	6q26 (plasminogen gene (Glu460Stop mutation))
Schuster et al., 1997 [335]	Homozygous mutations in the plasminogen gene of two unrelated girls with ligneous conjunctivitis	Children's Hospital, University of Würzburg, Germany	2 Subjects, 2 Parents, 1 Sister, 1 Control	Turkish	Case study	Macrocephaly	Pseudomembranous lesions of other mucous membranes in the mouth, nasopharynx, trachea, and female genital tract	Obstructive	TES	SSCP, restriction enzyme analysis	AR	6q26 (Plasminogen gene)
Schuster et al., 1999 [336]	Prenatal diagnosis in a family with severe type I plasminogen deficiency, ligneous conjunctivitis and congenital hydrocephalus	Children's Hospital, University of Würzburg, Germany	1 Subject, 2 Parents, 1 Control	Turkish	Case study	Isolated hydrocephalus	Pseudomembranous conjunctivitis, ligneous conjunctivitis	Obstructive	TES	SSCP	AR	6q26 (Plasminogen gene)
Tonduti et al., 2015 [337]	Cystic leukoencephalopathy with cortical dysplasia related to LAMB1 mutations	Université Paris Diderot-Sorbonne Paris Cité and INSERM U1141-DHU Protect, Paris, France	2 Subjects, 100 Control	-	Case series	Cerebral palsy, epilepsy, spastic tetraplegia, intellectual disability	Lens opacification, optic atrophy	Unclear	WES	Sanger sequencing, segregation analysis	-	7q31.1 (LAMB1)
Van der Knaap et al., 2006 [338]	Neonatal porencephaly and adult stroke related to mutations in collagen IV A1	VU University Medical Center, Amsterdam, the Netherlands	3 Subjects, 192 Controls	Dutch	Case series	Leukoencephalopathy, porencephalic cysts, cerebral microangiopathies	Cataracts, blood vessel defects	Obstructive (blood, calcifications) vs. Porencephaly	-	-	AD	13q34 (mutation in the *COL4A1)*
Yang et al., 2017 [339]	Novel FREM1 mutations are associated with severe hydrocephalus and shortened limbs in a prenatal case	The First Affiliated Hospital of Jinan University, Guangzhou, Guangdong, China	1 Subject, 200 Controls	Chinese	Case study	Isolated hydrocephalus	Short limbs	-	WES	Sanger sequencing	-	9p22.3 (FREM1)

Array comparative genomic hybridization (aCGH). Autosomal Dominant (AD). Autosomal Recessive (AR). Single-strand conformation polymorphisms (SSCP). Targeted exome sequencing (TES). Targeted genome sequencing (TGS). Whole exome sequencing (WES). Whole genome sequencing (WGS)

### Neurogenesis and neural stem cell biology

Table 11 summaries gene mutations implicating neurogenesis. Mutations were identified in SRY-box transcription factor 9 (*SOX9*), solute carrier family 29 member 3 (*SLC29A3*), adhesion G protein-coupled receptor (*ADGRG1*), katanin interacting protein (*KIAA0556*), G protein signaling modulator 2 (*GPSM2*), tripartite motif containing 71 (*TRIM71*) [80], SWI/SNF related, matrix associated, actin dependent regulator of chromatin subfamily c member 1 (*SMARCC1*) [81], patched 1 (*PTCH1*), FLVCR heme transporter 2 (*FLVCR2*), intestinal cell kinase (*ICK*), cystathionine beta-synthase (*CBS*), 5-methyltetrahydrofolate-homocysteine methyltransferase reductase (*MTRR*), interleukin 4 induced 1 (*IL4I1*), scribble planar cell polarity protein (*SCRIB1*), protein tyrosine kinase 7 (*PTK7*), frizzled class receptor 1 (*FZD1*), VANGL planar cell polarity protein 2 (*VANGL2*), dishevelled segment polarity protein (*DVL2*), transcription elongation factor B polypeptide 3B (*TCEB3B*), phospholipase C delta 4 (*PLCD4*), Ras associated domain family member 4 (*RASSF4*), phenylalanyl-tRNA synthetase 2, mitochondrial (*FARS2*), tubulin beta 3 class III (*TUBB3*), and discs large MAGUK scaffold protein 5 (*DLG5*). Frameshift mutations were seen in WD repeat domain 81 (*WDR81*), kinase D interacting substrate 220 (*KIDINS220*). Deletions were seen in chromosome 6 (6q25.3 and 6p25), chromosome 13 (13q), chromosome 16 (16p12.2), and chromosome 22 (22q11.2). The 6p25 deletion resulted in the deletion of forkhead box C1 (*FOXC1*), forkhead box F2 (*FOXF2*), and forkhead box Q1 (*FOXQ1*).

**Table 11:** Neurogenesis

Citation	Title	Author affiliation	Case #	Ancestry	Study design	CNS phenotype	Non-CNS phenotype	Type of hydrocephalus	Genetic methodology	Genetic analysis	Inheritance	Genetic findings
Antwi et al., 2018 [340]	A novel association of campomelic dysplasia and hydrocephalus with an unbalanced chromosomal translocation upstream of SOX9	Yale University, New Haven, CT, United States	1 Subject	-	Case study	Hypoplastic C6 vertebral body, exaggerated cervical lordosis, and exaggerated thoracic kyphosis	Tracheobronchomalacia, cleft palate, retrognathia, hypertelorism, hypoplastic mandible	Communicating	WES, cytogenetics	Karyotyping, FISH, aCGH	De novo	17q24.3 (SOX9)
Avitan-Hersh et al., 2011 [341]	A case of H syndrome showing immunophenotye similarities to Rosai-Dorfman disease	Technion Institute of Technology, Haifa, Israel	1 Subject, 2 Parents	Arab	Case study	Isolated hydrocephalus	Pulmonic stenosis, skin hyperpigmentation, hepatomegaly, splenomegaly, dilatation of the right renal pelvis	Communicating	TES	-	AR	10q22.1 (SLC29A3 gene, encodes human equilibrative nucleoside transporter hENT3)
Cauley et al., 2019 [342]	Overlap of polymicrogyria, hydrocephalus, and Joubert syndrome in a family with novel truncating mutations in ADGRG1/GPR56 and KIAA0556	The George Washington University School of Medicine and Health Sciences, Washington, DC, USA	2 Subjects, 2 Siblings, 2 Parents, Controls used	Sudanese	Case series	Psychomotor delay, intellectual disability, seizures, severe brain malformations, spasticity, hyperreflexia	Ptosis, unilateral ophthalmoplegia, and bilateral vertical ophthalmoplegia, muscle wasting	-	WES	Variant analysis, Sanger sequencing	AR	16q21 (ADGRG1) And 16p12.1 (KIAA0556)
Christofolini et al., 2006 [343]	Hydrocephaly, penoscrotal transposition, and digital anomalies associated with de novo pseudodicentric rearranged chromosome 13 characterized by classical cytogenetic methods and mBAND analysis	Departamento de Morfologia, Disciplina de Genética, Universidade Federal de São Paulo, São Paulo, Brazil	1 Subject, 2 Parents	-	Case study	Corpus callosum agenesis	Imperforate anus with anocutaneous fistula, penoscrotal transposition, and digital reduction defects, short palpebral fissures, telecanthus, epicanthic folds, short nose with depressed nasal bridge and anteverted nostrils, posteriorly rotated ears, short neck	Obstructive	Cytogenetics	G-banding	De novo	13q deletion
Doherty et al., 2012 [344]	GPSM2 mutations cause the brain malformations and hearing loss in Chudley-McCullough syndrome	University of Washington, Seattle Children's Hospital, USA	12 Subjects, Controls used	Mennonite, European American, Dutch	Case series	Bilateral sensorineural deafness, corpus callosum agenesis, arachnoid cysts, posterior agenesis of the corpus callosum, frontal polymicrogyria, frontal heterotopia, cerebellar dysplasia	Down slanting palpebral fissures and low-set, posteriorly rotated ears	Communicating, Obstructive	Genotyping, WES	SNP, sanger sequencing	AR	1p13.3 (G protein-signaling modulator 2 gene, GPSM2)
Forrester et al., 2002 [345]	Kousseff syndrome caused by deletion of chromosome 22q11-13	Southern Illinois University School of Medicine, Springfield, Illinois, USA	3 Subjects, 2 Controls	-	Case series	Intellectual disability	Lumbosacral myelomeningocele, cleft palate, and dysmorphic features consisting of low-set and posteriorly rotated ears, retrognathia, and clinodactyly of the fifth toes, cardiac anomalies	Obstructive	Genotyping, cytogenetics	FISH, karyotyping, microsatellite marker assay	AR	22q11.2-microdeletion
Furey et al., 2018 [8]	De Novo Mutation in Genes Regulating Neural Stem Cell Fate in Human Congenital Hydrocephalus	Yale University School of Medicine, New Haven, CT 06510, USA	177 subjects, 1,789 controls	-	Case series	Isolated hydrocephalus		Communicating, Obstructive	WES	Direct sequencing	De novo	3p22.3 (TRIM71) 3p21.31 (SMARCC1 9q22.32 (PTCH1)
Grosso et al., 2002 [346]	De novo complete trisomy 5p: clinical and neuroradiological findings	University of Siena, Siena, Italy	1 Subject	-	Case study	Isolated hydrocephalus	Low-set, posteriorly rotated ears with reduced cartilage, up slanted palpebral fissures, epicanthus, hypertelorism, a wide and depressed nasal root, a short nose with anteverted nostrils, a long philtrum, retrognathia, an ogival palate, a short neck, abnormal palmar creases, and a bell-shaped trunk	-	Cytogenetics	FISH w/ WCP	De novo	trisomy 5p
Jacquemin et al., 2020 [347]	TrkA mediates effect of novel KIDINS220 mutation in human brain ventriculomegaly	Université Libre de Bruxelles, 1070 Brussels, Belgium	3 Subjects, 1 Control	Pakistan	Case series	Isolated hydrocephalus	Limb contractures, club feet	-	WES	Variant analysis	AR	2p25.1 (homozygous variant of KIDINS220)
Kline-Fath et al., 2018 [348]	Fowler syndrome and fetal MRI findings: a genetic disorder mimicking hydranencephaly/hydrocephalus	Cincinnati Children's Hospital Medical Center, 3333 Burnet Ave., Cincinnati, OH, USA	1 Subject	-	Case study	Thin cerebral cortex, cerebellum, brainstem, and spinal cord	Arthrogryposis, proliferative glomeruloid vasculopathy	Obstructive	WES	-	AR	14q24.3 (FLVCR2 gene)
Koenigstein et al., 2016 [349]	Chudley-McCullough Syndrome: Variable Clinical Picture in Twins with a Novel GPSM2 Mutation	Justus-Liebig-University, Giessen, Germany	2 Subjects	Turkish	Case series	Callosal agenesis, interhemispheric cyst, frontal polymicrogyria	Sensorineural deafness	Communicating	-	-	AR	1p13.3 (c.C1093T; p.R365X in GPSM2)
Lahiry et al., 2009 [350]	A multiplex human syndrome implicates a key role for intestinal cell kinase in development of central nervous, skeletal, and endocrine systems	Robarts Research Institute, London, Ontario N6A 5K8, Canada	6 Subjects, 3112 Controls	Amish	Case series	Cerebral anomalies	Facial dysmorphisms, eye anomalies, skeletal anomalies, pulmonary/GI/GU dysplasia	Communicating	Genotyping, TGS	SNP, autozygosity mapping, direct sequencing	AR	6p12.1 (ICK p.R272Q mutation)
Li et al., 2015 [351]	Congenital hydrocephalus and hemivertebrae associated with de novo partial monosomy 6q (6q25.3 → qter)	The Third Affiliated Hospital of Guangzhou Medical University, Guangzhou, Guangdong, People's Republic of China	1 Subject	-	Case study	Isolated hydrocephalus	Lumbar hemivertebrae	-	Cytogenetics	CNV, aCGH, Karyotyping, FISH	-	deletion in chromosome region 6q25.3 → qter
Maclean et al., 2004 (258)	Kousseff syndrome: a causally heterogeneous disorder	Sydney Children's Hospital, Sydney, Australia	2 Subjects	Indonesian	Case series	Myelomeningocele, callosal hypoplasia, intellectual delay	Posteriorly rotated ears, a large nose, a smooth featureless philtrum, hypertrichosis and restricted ankle dorsiflexion, tetralogy of fallot	Obstructive	TES	Cycle sequencing	AR	22q11.2-microdeletion
Maclean et al., 2005 [352]	Axenfeld-Rieger malformation and distinctive facial features: Clues to a recognizable 6p25 microdeletion syndrome	The Children's Hospital at Westmead, Sydney, New South Wales, Australia	1 Subject	Caucasian	Case study	Cerebellar hypoplasia, a deficient inferior vermis, hypoplasia of the pons, medulla, and posterior corpus callosum, and absent septum pellucidum	Axenfeld-rieger malformation, hearing loss, congenital heart disease, dental anomalies, developmental delay, and a characteristic facial appearance	Communicating	Cytogenetics, genotyping	Karyotyping, FISH, microsatellite marker assay,	De novo	6p25 (deletion of the FOXC1/FOXF2/FOXQ1 forkhead gene cluster)
Mero et al., 2017 [353]	Homozygous KIDINS220 loss-of-function variants in fetuses with cerebral ventriculomegaly and limb contractures	Oslo University Hospital, Oslo, Norway	4 Subjects, 2 Parents	-	Case series	Callosum agenesis, small cerebellum	Limb contractures	Communicating	WES	Sanger sequencing, autozygosity mapping,	AR	2p25.1 (homozygous frameshift variant in exon 24 in KIDINS220)
Pappa et al., 2017 [354]	Exome analysis in an Estonian multiplex family with neural tube defects-a case report	University of Tartu, Riia 23b, 51,010, Tartu, Estonia	3 Subjects, 2 Parents	Estonian	Case series	Spina bifida, aqueductal stenosis, intellectual delay	Gait and motor abnormalities	Obstructive	WES	Variant analysis	Maternal	21q22.3 (CBS), 5p15.31 (MTRR), 1p36.22 (MTHFR), 19q13.33 (IL4I1), 8q24.3 (SCRIB1), 6p21.1 (PTK7), 7q21.13 (FZD1), 1q23.2 (VANGL2), 17p13.1 (DVL2), 18q21.1 (TCEB3B), 2q35 (PLCD4), 10q11.21 (RASSF4), and 6p25.1 (FARS2)
Powis et al., 2018 [355]	Postmortem Diagnostic Exome Sequencing Identifies a De Novo TUBB3 Alteration in a Newborn with Prenatally Diagnosed Hydrocephalus and Suspected Walker-Warburg Syndrome	Ambry Genetics, Aliso Viejo, California, USA	1 Subject, 2 Parents	Caucasian	Case study	Posterior fossa cyst, dandy walker malformation, seizures	Optic nerve abnormalities, abnormal renal function	-	Diagnostic exome sequencing	-	De novo	16q24.3 (TUBB3)
Rai et al., 2015 [356]	Cervicomedullary spinal stenosis and ventriculomegaly in a child with developmental delay due to chromosome 16p12.1 microdeletion syndrome	Midland Regional Hospital, Mullingar Westmeath, Ireland	1 Subject	-	Case study	Macrocephaly	Significant delay in gross motor skills	-	Cytogenetics	aCGH	-	Chr. 16p12.2 deletion
Su et al., 2021 [357]	Novel compound heterozygous frameshift variants in WDR81 associated with congenital hydrocephalus 3 with brain anomalies: First Chinese prenatal case confirms WDR81 involvement	Guangxi Health Commission Key Laboratory of Precise Diagnosis and Treatment of Genetic Diseases, Maternal and Child Health Hospital of Guangxi Zhuang Autonomous Region, Nanning, China	2 Subjects	Chinese	Case series	Cerebellar hypoplasia	Cleft lip and palate, hydrops fetalis, hepatomegaly	HYC3	WES	Sanger sequencing, variant analysis	AR	17p13.3 (WDR81)
Yüksel et al., 2019 [358]	A homozygous frameshift variant in an alternatively spliced exon of DLG5 causes hydrocephalus and renal dysplasia	Centogene AG, Rostock, Germany	1 Subject, 1 Control	-	Case study	Isolated hydrocephalus	Atrial and ventricular septal defects, cleft lip and palate, and a renal phenotype including multi-cystic dysplasia	Obstructive	WES	Variant analysis, sanger sequencing	De novo	10q22.3 (DLG5)

Array comparative genomic hybridization (aCGH). Autosomal Recessive (AR). Copy number variant (CNV). Fluorescence In Situ Hybridization (FISH). Single nucleotide polymorphisms (SNP). Targeted exome sequencing (TES). Targeted genome sequencing (TGS). Whole chromosome probes (WCP). Whole exome sequencing (WES). Whole genome sequencing (WGS)

The heterogeneity of neurogenesis-associated HC suggests that numerous genes involved in development may confer susceptibility to this phenotype. *SOX9* knockdown in mice suggest a role in neural stem cell development and ependymal cell maintenance as a pathogenic mechanism that causes HC [82]. In addition, mutations in *ADGRG1* have been shown to impact cerebral cortex development and neuronal migration via the perturbation of the RhoA pathway [83]. In addition, *GPSM2* has been shown to alter neuroepithelial function through disruption of cellular orientation and planarity leading to aberrant brain development [84]. Finally, mice lacking *KIDINS220* display attenuated responses to neurotrophic factors and have impaired development in multiple signaling pathways [85]. Understanding the genetic influence of neurogenesis may elucidate a better understanding of patient characteristics and poor outcomes in the HC phenotype [86–89].

### Inherited cancer syndromes

Table 12 summarizes genes that contributed to tumor pathogenesis, and which result in the development of HC. Mutations are seen in NRAS proto-oncogene, GTPase (*NRAS*), von Hippel-Lindau tumor suppressor (*VHL*), patched 1 (*PTCH1*) and FA complementation group C (*FANCC*). Germline mutations are seen in phosphatase and tensin homolog (*PTEN*) and SUFU negative regulator of hedgehog signaling (*SUFU*). Deletions within chromosome 11 (11p13) and chromosome 9 (9q22.3 and 9q22-q31) were also identified. *NRAS* is an oncogene contributing to the development of congenital melanocytic nevi, a condition associated with HC [90]. Clinically relevant mutations in Von Hippau Lindau (*VHL*) affect protein expression and degradation where patients with or without a mass lesion (i.e., hemangioblastoma) develop HC [91]. Gorlin syndrome is disorder characterized with bony abnormalities and an increased risk for multiple CNS and non-CNS tumors. Previous studies have mapped this syndrome to deletions in the 9q22 locus which is consistent with the patients identified in this review with mutations specifically affecting *PTCH1* and *FANCC* genes [92, 93]. Finally, mutations in SUFU have also been associated with Gorlin syndrome [94, 95].

**Table 12:** Inherited cancer syndromes

Citation	Title	Author affiliation	Case #	Ancestry	Study design	CNS phenotype	Non-CNS phenotype	Type of hydrocephalus	Genetic methodology	Genetic analysis	Inheritance	Genetic findings
Demir et al., 2011 [359]	WAGR syndrome with tetralogy of Fallot and hydrocephalus	Hacettepe University, Ankara, Turkey	1 Subject	-	Case study	Isolated hydrocephalus	Wilms tumor, aniridia, genitourinary abnormalities, and intellectual disability	Communicating	Cytogenetics	G-banding	De novo	deletion of chromosome 11p13
Fukino et al., 2000 [360]	A family with hydrocephalus as a complication of cerebellar hemangioblastoma: identification of Pro157Leu mutation in the VHL gene	Nippon Medical School, Kawasaki-shi, Japan	2 Subjects	Japanese	Case series	Isolated hydrocephalus	Retinal angioma, cerebellar, hemangioblastomas, pancreatic cysts	Obstructive	TES	Direct sequencing, restriction enzyme analysis	-	3p25.3 (VHL)
Kinsler et al., 2013 [90]	Multiple congenital melanocytic nevi and neurocutaneous melanosis are caused by postzygotic mutations in codon 61 of NRAS	Great Ormond Street Hospital for Children, London, UK	5 Subjects, Controls used	-	Case series	Arachnoid cysts, syringomyelia, tumors (including astrocytoma, choroid plexus papilloma, ependymoma, and pineal germinoma), Dandy–Walker, and Chiari malformation	Widespread melanocytic nevi	Communicating, Obstructing	TGS, cytogenetics	aCGH, direct sequencing	Non-mendelian inheritance	1p13.2 (c.181C > A, p.Q61K NRAS mutations)
Kusakabe et al., 2018 [361]	Combined morphological, immunohistochemical and genetic analyses of medulloepithelioma in the posterior cranial fossa	Ehime University School of Medicine, Toon, Japan	1 Subject	-	Case study	Medulloepithelioma		Obstructive	Cytogenetics	FISH	-	No C19MC mutations
Pastorino et al., 2009 [94]	Identification of a SUFU germline mutation in a family with Gorlin syndrome	Università degli Studi di Genova, Genova, Italy	1 Subject, 1 Control	Caucasian	Case study	Spina bifida	Pits in hands and soles, coarse facies, strabismus, cleft lip and palate, bifid ribs	Obstructive	TGS	MPLA, direct sequencing	AD	10q24.32 (c.1022 + 1G > A SUFU germ line splicing mutation)
Reardon et al., 2001 [362]	A novel germline mutation of the PTEN gene in a patient with macrocephaly, ventricular dilatation, and features of VATER association	Our Lady's Hospital for Sick Children, Crumlin, Dublin 12, Ireland	1 Subject, 2 Parents	-	Case study	Macrocephaly	Hypoplasia of the thumbs bilaterally with radial deviation of the hands, 13 pairs of ribs	Communicating	Cytogenetics, TGS	Karyotyping	AD	10q23.31 PTEN
Reichert et al., 2015 [92]	Diagnosis of 9q22.3 microdeletion syndrome in utero following identification of craniosynostosis, overgrowth, and skeletal anomalies	Children's Hospital of Philadelphia, Philadelphia, Pennsylvania	1 Subject, 2 Parents	-	Case study	Metopic craniosynostosis, intellectual disability, Trigonocephaly	Macrosomia, hepatomegaly, nephromegaly, and anomalous vertebrae	Communicating	Cytogenetics	Karyotyping, SNP, FISH	De novo	9q22.32 (PTCH1) and 9q22.32 (FANCC) genes 9q22.3
Shimkets et al., 1996 [93]	Molecular analysis of chromosome 9q deletions in two Gorlin syndrome patients	Yale University School of Medicine, New Haven, CT 06520–8005, USA	2 Subjects, 4 Parents	African American, Caucasian	Case series	Macrocephalus, agenesis of the corpus callosum	Bilateral inguinal hernias, bilateral conductive hearing loss, strabismus, and ectopic eruption of the upper central incisors, multiple basal cell carcinomas, medulloblastomas, ovarian fibromas	Communicating	Cytogenetics, genotyping,	G-banding, restriction enzyme analysis	AD	chromosome 9q22 deletion and 9q22-q3l
Uguen et al., 2015 [363]	Severe hydrocephalus caused by diffuse leptomeningeal and neurocutaneous melanocytosis of antenatal onset: a clinical, pathologic, and molecular study of 2 cases	Service d'anatomie et cytologie pathologiques, Brest, F-29220 France; Université Européenne de Bretagne, 29,238 France	2 Subjects	-	Case series	Leptomeningeal pigmentation, Dandy walker malformation	Melanocytic nevi	Obstructive	Cytogenetics, TGS	aCGH, FISH, pyrosequencing, NGS	-	1p13.2 (NRAS)

Autosomal Dominant (AD). Fluorescence In Situ Hybridization (FISH). Multiplex ligation dependent probe amplification (MLPA). Single nucleotide polymorphisms (SNP). Targeted genome sequencing (TGS)

### WNT signaling

WNT signal transduction is involved in numerous pathways regulating cell function and development. Table 13 summarizes gene mutations identified in HC patients with this pathway. Numerous studies have reported gene mutations in coiled-coil and C2 domain containing 2A (*CC2D2A*) and coiled-coil domain containing 88C (*CCDC88C*). COACH syndrome is defined as cerebellar vermis hypoplasia, oligophrenia, ataxia, colobomas, and hepatic fibrosis [96]. This gene locus has been shown to interact with the WNT signaling pathway and is associated with centrosome stability [97]. In addition, *CCDC88C* is associated with the WNT signaling pathway through interaction with the Dishevelled protein [98]. The dishevelled protein contains a binding domain which interacts with a hook related protein transcribed from the *CCDC88C* locus [99]. WNT signaling plays numerous roles in cell communication and embryonic development, suggesting potential mechanisms contributing to HC [100].

**Table 13:** WNT signaling

Citation	Title	Author affiliation	Case #	Ancestry	Study design	CNS phenotype	Non-CNS phenotype	Type of hydrocephalus	Genetic methodology	Genetic analysis	Inheritance	Genetic finding
Doherty et al., 2010 [96]	Mutations in 3 genes (MKS3, CC2D2A and RPGRIP1L) cause COACH syndrome (Joubert syndrome with congenital hepatic fibrosis)	University of Washington, Seattle Children's Hospital, USA	26 Subjects, 210 Controls	USA, European, Asian, African, Native American, Italy, the Netherlands, Germany, UK, and Turkey	Case series	Intellectual impairment, hypotonia, ataxia, cerebellar vermis hypoplasia, encephalocele	Congestive heart failure, hepatic fibrosis, coloboma, retinal disease, renal disease, polydactyly	Communicating	Genotyping, TGS	Microsatellite marker assay, SNP	AR	4p15.32 (CC2D2A)
Drielsma et al., 2012 [364]	Two novel CCDC88C mutations confirm the role of DAPLE in autosomal recessive congenital hydrocephalus	Institute of Interdisciplinary Research – IRIBHM, Université Libre de Bruxelles, Brussels, Belgium	8 Subjects, 4 Parents, 721 Controls	Jewish Ashkenazi, Palestinian,	Case series	Seizures, parietal polymicrogyria	Hypertelorism, lung lymphangiectasias	Communicating	Cytogenetics, genotyping, TGS	Karyotyping, MLPA, homozygosity mapping, sanger sequencing	AR	14q32.11-q32.12 (CCDC88C)
Ekici et al., 2010 [98]	Disturbed Wnt Signalling due to a Mutation in CCDC88C Causes an Autosomal Recessive Non-Syndromic Hydrocephalus with Medial Diverticulum	University of Regensburg, Regensburg, Germany	58 subjects, 224 controls	Algeria	Case series	Mild psychomotor delay	-	-	Genotyping, TGS	Linkage analysis, homozygosity mapping, cycle sequencing	AR	14q32.11-q32.12 (CCDC88C)
Ruggeri et al., 2018 [365]	Bi-allelic mutations of CCDC88C are a rare cause of severe congenital hydrocephalus	Seattle Children's Research Institute, Seattle, Washington	2 Subjects	-	Case series	Intellectual delay and infantile onset seizures	Varying degrees of motor delay	-	WES	Variant analysis, trio-based exome sequencing, sanger sequencing	AR	14q32.11-q32.12 (CCDC88C)
Wallis et al., 2018 [366]	Surprisingly good outcome in antenatal diagnosis of severe hydrocephalus related to CCDC88C deficiency	Austin Health, Heidelberg, Victoria, Australia	5 Subjects	Moroccan, Saudi	Case series	Isolated hydrocephalus	Developmental delay	Obstructive	TGS	Massively parallel sequencing	AR	14q32.11-q32.12 (CCDC88C)

Autosomal Recessive (AR). Multiplex ligation dependent probe amplification (MLPA). Single nucleotide polymorphisms (SNP). Targeted genome sequencing (TGS). Whole exome sequencing (WES)

### Transcriptional, post-transcriptional, and epigenetic regulation

Table 14 summarizes mutations in genes that regulate transcription, post-transcriptional, and epigenetic processes. Missense mutations were seen in THO complex subunit 6 (*THOC6*) and *HYLS1*, genes involved in transcriptional regulation. Patients with loss of function mutations in FA complementation group L (*FANCL*) we identified. Additional mutations observed included interferon regulatory factory 6 (*IRF6*), small nucleolar RNA, C/D box 118 (*SNORD118*), nuclear factor I A (*NFIA*), SET binding protein 1 (*SETBP1*), SWI/SNF related, matrix associated, actin dependent regulator of chromatin, subfamily b, member 1 (*SMARCB1*), maelstrom spermatogenic transposon (*MAEL*), a deletion in chromosome 5 (5q35.3), and 20q13.3 trisomy. Deletions in chromosome 1 (1q42.3-q44) resulted in the deletion of zinc finger and BTB domain containing 18 (*ZBTB18*) and heterogeneous nuclear ribonucleoprotein U (*HNRNPU*). THOC6 is a part the TREX complex responsible for mRNA export and is localized to the 5’ cap of mRNA [101]. It has been associated with Beaulieu-Boycott-Innes syndrome, which is charactered by developmental delay and organ dysgenesis [102]. Patients identified in this review with Beaulieu-Boycott-Innes syndrome and *THOC6* mutations have been shown to develop HC, suggesting a role for mRNA export regulation in association with HC phenotypes [103]. *HYLS1* is associated with Hydrolethalus syndrome, a disorder characterized by HC and craniofacial abnormalities [104]. Expression analysis of this gene suggests a role in CNS development, where a HC associated mutation gene causes nuclear localization whereas the WT form is expressed in the cytoplasm [104]. *SNORD118* is involved in regulation of ribosome biology and associated with the hydrocephalic phenotype of Labrune syndrome, characterized by leukoencephalopathy, intracranial cysts, and calcification [105]. While the function of *SETBP1* remains largely unknown, mutations in this gene are associated with Schinzel-Giedion syndrome, characterized by facial abnormalities, intellectual disability, congenital malformations, and HC [106]. *SMARCB1* is involved in chromatin remodeling to further enhance or repress transcription [107]. Finally, a transcriptome-wide association study (TWAS) and multi-omics study of HC identified maelstrom (*MAEL*), a gene that regulates transposons and epigenetic modifications, as an experiment-wide predictor of HC in the cortex [9, 108]. These studies identified transcriptional regulators and further emphasize the need to explore these mechanisms to understand the mechanistic associations with HC.

**Table 14:** Transcriptional, post-transcriptional, and epigenetic regulation

Citation	Title	Author affiliation	Case #	Ancestry	Study design	CNS phenotype	Non-CNS phenotype	Type of hydrocephalus	Genetic methodology	Genetic analysis	Inheritance	Genetic finding
Chen et al., 2020 [367]	Prenatal diagnosis and molecular cytogenetic characterization of a chromosome 1q42.3-q44 deletion in a fetus associated with ventriculomegaly on prenatal ultrasound	Mackay Memorial Hospital, Taipei, Taiwan	1 Subject, 2 Parents	-	Case study	Anomalies of corpus callosum, microcephaly	Hypertelorism, large low-set ears, micrognathia, a broad nose, arched eyebrows, prominent forehead and flat nasal bridge	-	Cytogenetics	aCGH, FISH, polymorphic DNA marker analysis	Paternal	1q42.3-q44 deletion (including 1q43 (RGS7), 1q43 (FH), 1q43 (CEP170), 1q43-44 (AKT3), 1q44 (ZBTB18 and 1q44 (HNRNPU))
Diets et al., 2019 [368]	A recurrent de novo missense pathogenic variant in SMARCB1 causes severe intellectual disability and choroid plexus hyperplasia with resultant hydrocephalus	Radboud University Medical Center and Radboud Institute for Molecular Life Sciences, Nijmegen, The Netherlands	4 Subjects	-	Case series	Choroid plexus hyperplasia w/ papilloma, truncal hypotonia, intellectual disability	Visual impairment, myopia, sleep apnea, joint hypermobility, renal and cardiac anomalies	-	WES	Trio-based exome sequencing	De novo	22q11.23 (SMARCB1)
Hale et al., 2021 [9]	Multi-omic analysis elucidates the genetic basis of hydrocephalus	Vanderbilt University School of Medicine, Medical Scientist Training Program, Nashville, TN	287 Subjects, 18,740 Controls	European	Case series	Various neurological phenotypes		Variable	Gene expression	PrediXcan analysis	Variable	1q24.1 (MAEL)
Hishimura et al., 2016 [106]	Genetic and prenatal findings in two Japanese patients with Schinzel-Giedion syndrome	Tenshi Hospital N-12, E-3 Sapporo, Japan	2 Subjects	Japanese	Case series	Isolated hydrocephalus	Overlapping fingers, hydronephrosis. High, prominent forehead, hypertelorism, and depressed nasal root	-	TES, cytogenetics	G-banding	AD	18q12.3 (SETBP1)
Mattioli et al., 2019 [103]	Clinical and functional characterization of recurrent missense variants implicated in THOC6-related intellectual disability	Institut de Génétique et de Biologie Moléculaire et Cellulaire, 67,400 Illkirch-Graffenstaden, France	2 Subjects, controls used	European	Case series	Intellectual disability, multiple brain abnormalities	Facial dysmorphism, a cleft palate, micrognathia, choanal atresia, congenital heart defect, micropenis	Communicating	TGS, cytogenetics	Karyotyping, aCGH, SNP	AR	16p13.3 (THOC6 gene-Trp100Arg, Val234Leu, Gly275Asp)
Mee et al., 2005 [104]	Hydrolethalus syndrome is caused by a missense mutation in a novel gene HYLS1	David Geffen School of Medicine, University of California at Los Angeles, Los Angeles, CA, USA	24 subjects, 40 Controls	Finland	Case series	Absent midline structures of the brain	Micrognathia, polydactyly, Defective lobation of the lungs, anomalies of the respiratory tract, small chin and anomalous nose	Communicating	Genotyping, TGS	Microsatellite marker analysis, SNP, haplotype analysis, two-point linkage analysis	AR	11q24.2 (HYLS1 gene)
Negishi et al., 2015 [369]	Truncating mutation in NFIA causes brain malformation and urinary tract defects	Nagoya City University Graduate School of Medical Sciences, Nagoya, Japan	1 Subject, Control database used	-	Case study	Ventricular enlargement, callosal agenesis, urinary tract defects, mildly dysmorphic facial features	Urinary tract defects	Communicating	WES	Variant analysis, sanger sequencing	De novo	1p31.3 (de novo truncating mutation (c.1094delC; p.Pro365Hisfs*32) in the NFIA gene)
Nyboe et al., 2015 [370]	Familial craniosynostosis associated with a microdeletion involving the NFIA gene	Copenhagen University Hospital Rigshospitalet, Copenhagen, Denmark	4 Subjects	-	Case series	Hypoplasia of the corpus callosum, craniosynostosis, lambdoid synostosis	Dysmorphic features, renal defects	Obstructive	Cytogenetics	aCGH	De novo	1p31.3 (NFIA gene)
Shtaya et al., 2019 [371]	Leukoencephalopathy, Intracranial Calcifications, Cysts, and SNORD118 Mutation (Labrune Syndrome) with Obstructive Hydrocephalus	Neurosciences Research Centre, St. George's, University of London, London, United Kingdom; Atkinson Morley Neurosurgery Centre, St. George's University Hospital NHS Foundation Trust, London, United Kingdom	1 Subject	-	Case study	Widespread intracranial calcifications, cysts, and leukoencephalopathy	Motor developmental delay	Obstructive	-	-	-	17p13.1 (SNORD118
Verkerk et al., 2010 [372]	Unbalanced der(5)t(5;20) translocation associated with megalencephaly, perisylvian polymicrogyria, polydactyly and hydrocephalus	Erasmus Medical Center, Rotterdam, The Netherlands	2 Subjects	Dutch	Case series	Perisylvian polymicrogyria, megalencephaly	Asd, hypothalamic hypothyroidism, kyphoscoliosis, pectus carinatum and rickets, vesicoureteral reflux, high broad forehead, large fontanel, hypertelorism with epicanthic folds, short, upturned nose with hypoplastic nostrils, down turned corners of the mouth with thick vermilion of the lips, high arched palate, small, pointed chin with a vertical groove, large low-set ears, barrel shaped chest with kyphoscoliosis, postaxial polydactyly of the 5th right toe	-	Cytogenetics, WGS	Karyotyping, MLPA, FISH, SNP, CNV	-	5q35.3 deletion and 20q13.3 trisomy
Vetro et al., 2015 [373]	Loss-of-Function FANCL Mutations Associate with Severe Fanconi Anemia Overlapping the VACTERL Association	Biotechnology Research Laboratories, Fondazione IRCCS Policlinico San Matteo, Pavia, Italy	3 Subjects, 2 Parents, 3 Controls	Morocco, Dutch	Case series	Aqueductal stenosis, cerebellar hypoplasia	Bilateral radial and thumbs aplasia, hypoplasia of the left shoulder girdle, bilateral club feet, micrognathia, single and ectopic kidney, absent uterus, micropenis, hypoplastic lungs with abnormal lobation, tetralogy of fallot, ventricular septal defect and patent ductus arteriosus, esophageal atresia with tracheoesophageal fistula, anal atresia and rectovaginal fistula	-	WES	Sanger sequencing	AR	2p16.1 (FANCL truncating mutation)
Zechi-Ceide et al., 2007 [374]	Hydrocephalus and moderate mental retardation in a boy with Van der Woude phenotype and IRF6 gene mutation	Hospital de Reabilitação de Anomalias Craniofaciais Department of Biological Sciences, Universidade Estadual Paulista, Bauru Human Genome Center and Department of Genetics and Evolutionary Biology, Institute of Biosciences, USP, São Paulo, SP, Brazil	1 Subject, 2 Parents, Controls used	Finnish	Case study	Callosal hypoplasia, intellectual delay	Lip pits, distinct craniofacial dysmorphism with cleft lip and palate	-	TES	Segregation analysis, direct sequencing	AD w/ variable expressivity	1q32.2 (IRF6)

Array comparative genomic hybridization (aCGH). Autosomal Recessive (AR). Copy number variant (CNV). Fluorescence In Situ Hybridization (FISH). Multiplex ligation dependent probe amplification (MLPA). Single nucleotide polymorphisms (SNP). Targeted genome sequencing (TGS). Whole exome sequencing (WES). Whole genome sequencing (WGS)

### Ion transport and regulation

Table 15 summarizes gene mutations implicating ion transport. Mutations were seen in aquaporin 4 (*AQP4*) and FLVCR heme transporter 2 (*FLVCR2*). Mutations on chromosome 17 (17p13) implicated transient receptor potential cation channel subfamily V member 3 (*TRPV3*). Aquaporin 4 (*AQP4*) regulates water transport on ependymal cells and knockout of this gene in mice show disrupted gap junctions which alter the ependymal zone and CSF flow contributing to HC development [109; 110]. Mutations in the enhancer of *TMEM50b* alter expression of *TTF*, a direct transcriptional regulator of *AQP1*, have also been identified [101]. Mutations in *FLVCR2* are associated with Fowler’s syndrome, a disorder characterized by HC and hydranencephaly [111]. This gene locus encodes a transmembrane protein involved in solute transport, suggesting that defects in chemiosmotic regulation contribute to HC development [112].

**Table 15:** Ion transport and regulation

Citation	Title	Author affiliation	Case #	Ancestry	Study design	CNS phenotype	Non-CNS phenotype	Type of hydrocephalus	Genetic methodology	Genetic analysis	Inheritance	Genetic finding
Castañeyra-Ruiz1 et al., 2013 [375]	Aquaporin-4 expression in the cerebrospinal fluid in congenital human hydrocephalus	Facultad de Medicina, Universidad de La Laguna, La Laguna, Tenerife, Canary Island, Spain	13 Subjects, 4 Controls	-	Case series	Isolated hydrocephalus		Communicating, Obstructive	Gene expression	Western blot, ELISA assay	-	18q11.2 (AQP4)
Kvarnung et al., 2016 [376]	Mutations in FLVCR2 associated with Fowler syndrome and survival beyond infancy	Center for Molecular Medicine, Karolinska Institutet, Stockholm, Sweden	2 Subjects	Somalian	Case series	Intellectual disability, glomerular vasculopathy in the central nervous system, hypokinesia/akinesia	Arthrogryphosis	-	TES	Variant analysis, sanger sequencing	AR	14q24.3 (FLVCR2)
Lalonde et al., 2010 [377]	Unexpected allelic heterogeneity and spectrum of mutations in Fowler syndrome revealed by next-generation exome sequencing	McGill University and Genome Quebec Innovation Centre, Montreal, Canada	2 Subjects	French Canadian	Case series	CNS microcalcifications and hyperplastic microvessels forming glomeruloid structures	Arthrogryposis multiplex, webbing of joints, muscular atrophy	Obstructive	WES	Variant analysis, SNP	AR	14q24.3 (FLVCR2)
Martínez-Glez et al., 2010 [378]	Macrocephaly-capillary malformation: Analysis of 13 patients and review of the diagnostic criteria	Hospital Universitario La Paz, Madrid, Spain	13 Subjects	Spain	Case series	Megalencephaly, Chiari I, Sylvius aqueduct stenosis, polymicrogyria and hypocampic nodular hypocampic, septum pellucidum bifida, hemimegaloencephaly, tonsillar herniation, polymicrogyria, subependymal cyst	Both overgrowth/asymmetry, capillary malformations, skeletal abnormalities	Communicating	Cytogenetics, TGS, genotyping	G-banding, MLPA, SNP	-	17p13: *ABR*, *YWHAE*, *SMYD4*, *TRPV3*
Meyer et al., 2010 [111]	Mutations in FLVCR2 are associated with proliferative vasculopathy and hydranencephaly-hydrocephaly syndrome (Fowler syndrome)	Institute of Biomedical Research, University of Birmingham, Birmingham, UK	7 Subjects, 646 Controls	Pakistan	Case series	Hydranencephaly, brain stem, basal ganglia, and spinal cord diffuse clastic ischemic lesions with calcifications	Glomeruloid vasculopathy of the retinal vessel, akinesia deformation sequence (FADS) with muscular neurogenic atrophy	Obstructive	Genotyping	SNP, microsatellite marker assay	AR	14q24.3 (FLVCR2)
Özdemir et al., 2016 [379]	Neonatal Bartter syndrome with cholelithiasis and hydrocephalus: Rare association	Pamukkale University Faculty of Medicine, Denizli, Turkey	1 Subject	-	Case study	Isolated hydrocephalus	Renal abnormalities	Communicating	Cytogenetics	Karyotyping	AR	-
Thomas et al., 2010 [380]	High-throughput sequencing of a 4.1 Mb linkage interval reveals FLVCR2 deletions and mutations in lethal cerebral vasculopathy	Hôpital Necker-Enfants Malades, Paris, France	16 Subjects, 2 Controls	Turkish	Case series	Brain angiogenesis, hydranencephaly	Arthrogryposis/pterygia	Obstructive	TGS	Homozygosity mapping, SNP, cycle sequencing	AR	14q24.3 (FLVCR2)
Visapää et al., 1999 [381]	Assignment of the locus for hydrolethalus syndrome to a highly restricted region on 11q23-25	National Public Health Institute, Helsinki, Finland	15 Subjects, 20 Family Members, 41 Controls	Finnish	Case series	Absent midline structures of the brain	Micrognathia, polydactyly, anomalous eyes and nose, and a keyhole-shaped defect of the occipital bone, cleft lip or palate, anomalous or low-set ears, abnormal larynx or trachea, defective lobulation of the lungs, congenital heart defect, abnormal genitalia, and club feet	Communicating	Genotyping	Radiation-hybrid mapping, two-point and multipoint linkage analysis	AR	11q23-25

Autosomal recessive (AR). Single nucleotide polymorphisms (SNP). Targeted genome sequencing (TGS). Whole exome sequencing (WES)

### Normal pressure hydrocephalus

Normal pressure HC (NPH) is a form of communicating HC in which the progressive pressure of CSF is believed to result in in ventricular dilatation and further CSF accumulation. Table 16 summarizes the genes implicated in human studies of NPH. Scm like with four mbt domains 1 (*SFMBT1*) displayed an intron 2 deletion. Cilia and flagella associated protein 43 (*CFAP43*) was found to have a nonsense mutation. The gene locus contributing to the development of ETINPH, a disorder characterized with essential tremors and idiopathic NPH, was localized to 19q12-13.31 on chromosome 19. *SFMBT1* is highly expressed in ependymal cells and epithelial cells of the brain, suggesting that a mutation in this gene locus may contribute to the dysfunctional CSF circulation [113]. Furthermore, a binding site had been identified within intron 2 of this gene locus, suggesting that the deletion of this intron, as seen in our review, will impact function [114; 115]. Deletion of cell wall biogenesis 43 (*CWH43*) in humans has also been associated with NPH [116].

**Table 16:** Normal Pressure Hydrocephalus

Citation	Title	Author affiliation	Case #	Ancestry	Study design	CNS phenotype	Non-CNS phenotype	Type of hydrocephalus	Genetic Methodology	Genetic Analysis	Inheritance	Genetic Findings
Kato et al., 2011 [113]	Segmental copy number loss of SFMBT1 gene in elderly individuals with ventriculomegaly: a community-based study	Yamagata University Faculty of Medicine, Japan	8 Subjects, 10 Controls	Japanese	Case series	Isolated hydrocephalus		Communicating	WGS, cytogenetics	CNV, aCGH	*-*	3p21.1 (12 kb deletion within intron 2 of SFMBT1)
Morimoto et al., 2019 [382]	Nonsense mutation in *CFAP43* causes normal-pressure hydrocephalus with ciliary abnormalities	Kagawa University, Takamatsu, Japan	5 Subjects, Controls used	Japanese	Case study	Isolated hydrocephalus	Chronic sinusitis, pneumonia	Communicating	WES	Sanger sequencing	Heterozygous	10q25.1 (c.C105893468T in CFAP43)
Sato et al., 2016 [115]	A Segmental Copy Number Loss of the SFMBT1 Gene Is a Genetic Risk for Shunt-Responsive, Idiopathic Normal Pressure Hydrocephalus (iNPH): A Case–Control Study	Yamagata University Faculty of Medicine, Yamagata, Japan	50 Subjects, 110 Controls	Japanese	Case–Control	Isolated hydrocephalus		Communicating	TGS	CNV	De novo	3p21.1 (deletion in intron 2 of the SFMBT1)
Zhang et al., 2010 [383]	Genome-wide linkage scan maps ETINPH gene to chromosome 19q12-13.31	Center for Neurosciences, Texas Tech University Health Science Center, El Paso, TX, USA	26 Subjects	-	Case series	Essential tremor		Communicating	Genotyping	SNP, linkage analysis	AD	ETINPH locus localized to chromosome 19q12–13.31

Array comparative genomic hybridization (aCGH). Autosomal Dominant (AD). Copy number variant (CNV). Single nucleotide polymorphisms (SNP). Targeted genome sequencing (TGS). Whole exome sequencing (WES). Whole genome sequencing (WGS)

### Metabolism

Table 17 indicates genes involved in metabolic pathways. Mutations were seen in cytochrome c oxidase subunit 6B1 (*COX6B1*), methylenetetrahydrofolate reductase (*MTHFR),* and sulfatase modifying factor 1 (*SUMF1*). Mutations in *COX6B1* have been shown to disrupt the electron transport chain suggesting that alterations in cellular energetics can contribute to HC [117] [118]. *SUMF1* encodes formylglycine generating enzyme (FGE) involved in modifying cysteine residues in the endoplasmic reticulum [119]. *MTHFR* regulates folate metabolism, and mutations within this gene locus have been identified in congenital HC patients providing rationale to explore metabolic genes and their association with pathology [120].

**Table 17:** Metabolism

Citation	Title	Author affiliation	Case #	Ancestry	Study design	CNS phenotype	Non-CNS phenotype	Type of hydrocephalus	Genetic methodology	Genetic analysis	Inheritance	Genetic finding
Abdulhag et al., 2015 [117]	Mitochondrial complex IV deficiency, caused by mutated COX6B1, is associated with encephalomyopathy, hydrocephalus and cardiomyopathy	Hadassah-Hebrew University Medical Center, Jerusalem, Israel	1 Subject, 60 Controls	Palestinian	Case study	Hypotonia, cortical blindness	Symmetrical left ventricular hypertrophy tricuspid regurge and pulmonary hypertension	-	WES	Variant analysis, sanger sequencing	Mitochondrial inheritance	19q13.12 (COX6B1)
Cizmeci et al., 2013 [120]	Multiloculated hydrocephalus of intrauterine-onset: a case report of an unexpected MTHFR A1298C positive test result	Fatih University Medical School, Ankara, Turkey	1 Subject	-	Case study	Loculated hydrocephalus		Obstructive	-	-	-	1p36.22 (MTHFR A1298C homozygosity)
Schaaf et al., 2016 [384]	Desmosterolosis-phenotypic and molecular characterization of a third case and review of the literature	Baylor College of Medicine, Houston, Texas, USA	1 Subject, 1 Control, 2 Parents	-	Case study	Macrocephaly, thickening of the tectum and massa intermedia, effaced gyral pattern, underopercularization, thin corpus callosum	Arthrogryposis, disorder of cholesterol biosynthesis, bilateral fifth finger clinodactyly, mild cutaneous 2–4 toe syndactyly, and proximal placement of the great toes, and dysmorphic facial features	Obstructive	TGS	-	De novo	1p32.3 (compound heterozygote for c.281G > A (p.R94H) and c.1438G > A (p.E480K) mutations in DHCR24 gene)
Schlotawa et al., 2011 [119]	SUMF1 mutations affecting stability and activity of formylglycine generating enzyme predict clinical outcome in multiple sulfatase deficiency	Georg August University Göttingen, Göttingen, Germany	7 Subjects, Controls used	USA, Turkey, Switzerland, Pakistan	Case series	Intellectual disability, neurodegeneration	Skeletal changes, cardiac involvement, corneal clouding, organomegaly	Communicating	TGS	-	AR	3p26.1 (SUMF1)

Array comparative genomic hybridization (aCGH). Autosomal Recessive (AR). Fluorescence In Situ Hybridization (FISH). Multiplex ligation dependent probe amplification (MLPA). Single nucleotide polymorphisms (SNP). Targeted genome sequencing (TGS). Whole exome sequencing (WES)

### Cell cycle and cytoarchitecture

Table 18 displays genes involved in cell cycle regulation and cytoarchitecture. Mutations were seen in spindle apparatus coiled-coil protein 1 (*SPDL1*), tubulin alpha 3e (*TUBA3E*), nidogen 1 (*NID1*), tRNA splicing endonuclease subunit 15 (*TSEN15*), clathrin heavy chain linker domain containing 1 (*CLHC1*), TBC1 domain containing kinase (*TBCK*), xin actin binding repeat containing 1 (*XIRP1*), nucleoporin 107 (*NUP107*), erythrocyte membrane protein band 4.1 like 4A (*EPB41L4A*), protein phosphatase 2 regulatory subunit B delta (*PPP2R5D*), protein phosphatase 2 scaffold subunit Alpha (*PPP2R1A*), prolyl 4-hydroxylase subunit beta (*P4HB*), and crumbs cell polarity complex component 2 (*CRB2*). *SPDL1* has been shown to regulate mitotic checkpoints, and mutations arrested affected cells in metaphase [121]. *TUBA3E* maintains microtubule integrity by encoding for part of the microtubule heterodimer, alpha tubulin [122]. TSEN15 contributes to an endonuclease complex involved in tRNA splicing, and mutations affecting this gene locus can lead to defects in cell division [123]. *XIRP1* has been shown to maintain actin integrity and stability [124]. *P4HB* encodes an enzyme subunit involved in collagen formation, and mutations affecting this gene location are associated with reduced cytoarchitectural stability [125].

**Table 18:** Cell cycle and cytoarchitecture

Citation	Title	Author affiliation	Case #	Ancestry	Study design	CNS Phenotype	Non-CNS phenotype	Type of hydrocephalus	Genetic methodology	Genetic analysis	Inheritance	Genetic finding
Alazami et al., 2015 [385]	Accelerating novel candidate gene discovery in neurogenetic disorders via whole-exome sequencing of prescreened multiplex consanguineous families	King Faisal Specialist Hospital and Research Center, Riyadh, Saudi Arabia	143 Families	-	Case series	Global developmental delay, autism, epilepsy, primary microcephaly, ataxia, and neurodegeneration (*among many others*)	Wide Variability	Variable	WES	Autozygosity mapping, sanger sequencing	Variable	5q35.1 (SPDL1), 2q21.1 (TUBA3E), 15q15.1 (INO80), 1q42.3 (NID1), 1q25.3 (TSEN15), 1p33 (DMBX1), 2p16.1 (CLHC1), 12p13.32 (C12orf4), 15q26.1 (WDR93), 7q31.2 (ST7), 20q13.12 (MATN4), 4q26 (SEC24D), 5q31.3 (PCDHB4), 3p21.31 (PTPN23), 7q22.1 (TAF6), 4q24 (TBCK), 14q13.2 (FAM177A1), 4q27 (KIAA1109), 16q22.1 (MTSS1L), 3p22.2 (XIRP1), 1q41 (KCTD3), 21q22.12-q22.13 (CHAF1B), 1q42.2 (ARV1), 14q24.3 (ISCA2), 17q23.1 (PTRH2), 17p13.3 (GEMIN4), 17p12 (MYOCD), 16q22.1 (PDPR), 17p13.3 (DPH1), 12q15 (NUP107), 17q21.33 (TMEM92), 5q22.1-q22.2 (EPB41L4A), and 9q22.31 (FAM120AOS)
Houge et al., 2015 [386]	B56δ-related protein phosphatase 2A dysfunction identified in patients with intellectual disability	Haukeland University Hospital, Bergen, Norway	16 Subjects, Controls used	Dutch, English, Israeli, Norwegian	Case series	Intellectual disability, seizures, callosal agenesis, hypotonia	Frontal bossing, mild hypertelorism, and down slanting palpebral fissures	Communicating	Diagnostic exome sequencing, cytogenetics	Sanger sequencing, NGS, aCGH, SNP	De novo	6p21.1 (c.C157T, p.P53S; c.G592A, p.E198K; c.G598A, p.E200K; c.C602G, p.P201R; c.T619A, p.W207R in PPP2R5D); 19q13.41 (c.C536T, p.P179L; c.C544T, p.R182W; c.G773A, p.R528H in PPP2R1A)
Ouyang et al., 2017 [387]	Cole-Carpenter syndrome-1 with a de novo heterozygous deletion in the P4HB gene in a Chinese girl: A case report	West China Second University Hospital, Sichuan University, Chengdu, Sichuan, China	1 Subject, 2 Parents, 1 Control	Chinese	Case subject	Craniosynostosis	Plump anterior fontanel, growth retardation, osteopenia, and distinctive facial features, ocular proptosis, frontal bossing	-	WES	CNV, FQ-PCR	De novo	17q25.3 (P4HB)
Rauch et al., 2015 [388]	Cole-Carpenter syndrome is caused by a heterozygous missense mutation in P4HB	Shriners Hospital for Children, Montréal, QC H3G 1A6, Canada	2 Subjects, Controls used	-	Case series	Craniosynostosis	Bone fractures, ocular proptosis, and distinctive facial features	Communicating	WES	Variant analysis, sanger sequencing	De novo, Mosaic	17q25.3 (P4HB)
Slavotinek et al., 2015 (265) [389]	CRB2 mutations produce a phenotype resembling congenital nephrosis, Finnish type, with cerebral ventriculomegaly and raised alpha-fetoprotein	University of California, San Francisco, San Francisco, CA 94143–2711, USA	6 Subjects, 6 Parents	Ashkenazi Jewish	Case series	Gray matter heterotopias	Severe, Congenital renal involvement; congenital Nephrotic syndrome	-	WES, cytogenetics	aCGH, Karyotyping, variant analysis, sanger sequencing	AR	9q33.3 (CRB2)
Zhang et al., 2020 [390]	Genetic and preimplantation diagnosis of cystic kidney disease with ventriculomegaly	Children's Hospital of Shanxi and Women Health Center of Shanxi, Taiyuan, Shanxi, 030013, PR China	1 Subject, 2 Parents	Chinese	Case study	Isolated hydrocephalus	Echogenic kidneys and bowel, small fetal stomach bubble	-	WES	Variant analysis, sanger sequencing	-	9q33.3 (CRB2)

Array comparative genomic hybridization (aCGH). Autosomal Recessive (AR). Copy number variant (CNV). Fluorescence In Situ Hybridization (FISH). Fluorogenic quantitative-polymerase chain reaction (FQ-PCR). Next generation sequencing (NGS). Single nucleotide polymorphisms (SNP). Whole exome sequencing (WES)

### Lipid structure and regulation

Table 19 summarizes genes involved in lipid structure and regulation associated with HC in humans. Mutations were seen in bridge-like lipid transfer protein family member 1 (*KIAA1109*), and glucosylceramidase beta 1 (*GBA*). The *KIAA1109* ortholog in *Drosophila melanogaster* has shown to affect synaptic growth at the neuromuscular junction through modulation of phosphatidylinositol 4,5-bisphosphate (PIP^2^) [126]. *GBA* encodes for a lysosomal enzyme responsible for metabolizing glycolipids [127].

**Table 19:** Lipid structure and regulation

Citation	Title	Author affiliation	Case #	Ancestry	Study design	CNS phenotype	Non-CNS phenotype	Type of hydrocephalus	Genetic methodology	Genetic analysis	Inheritance	Genetic finding
Meszarosova et al., 2020 [391]	Two novel pathogenic variants in KIAA1109 causing Alkuraya-Kučinskas syndrome in two Czech Roma brothers	Second Faculty of Medicine Charles University and University Hospital Motol, Prague	2 Subjects	Roma	Case series	Hypotonia, cerebellar malformation, lissencephaly, callosum agenesis	Facial dysmorphic features, dysplastic ears, bilateral cataracts, finger contractures on both hands	-	WES	Variant analysis	AR	4q27 (KIAA1109)
Shiihara et al., 2000 [392]	Communicating Hydrocephalus in a Patient with Gaucher’s Disease Type 3	Institute of Neurological Sciences, Faculty of Medicine, Tottori University, Tottori, Japan	1 Subject, Controls used	Japanese	Case study	Isolated hydrocephalus	Splenomegaly, thrombocytopenia, bilateral papilledema, motor deficits	Communicating	TGS	Restriction enzyme analysis	-	1q22 (D409H mutation in GBA)

Autosomal Recessive (AR). Targeted genome sequencing (TGS). Whole exome sequencing (WES)

### Genes of unknown function

**Fig. 4:**
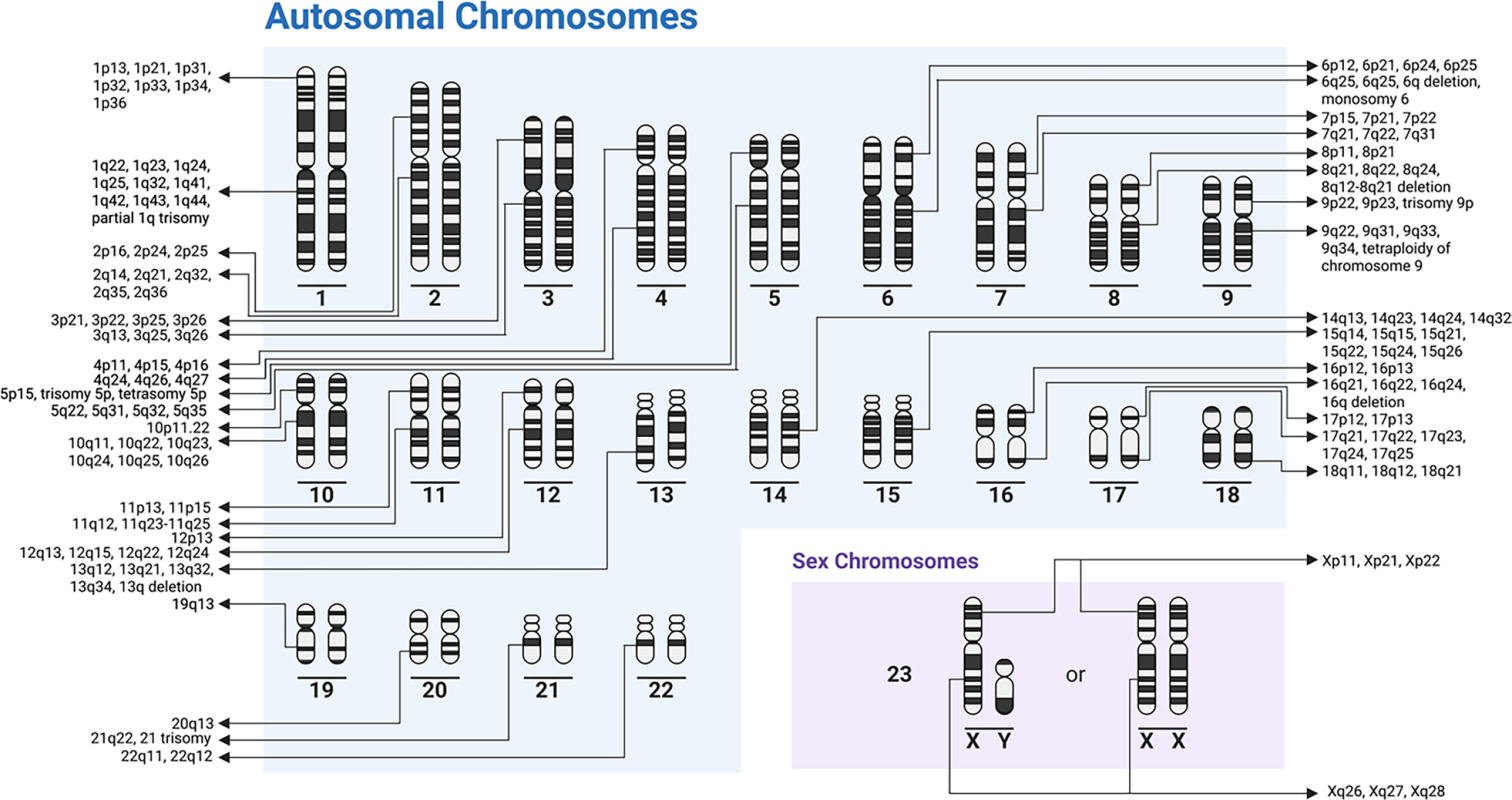
Chromosome map of hydrocephalus-associated loci across autosomal and sex chromosomes in humans

Table 20 summarizes genes that are associated with HC pathology without a clear function. Additional variants include partial 1q trisomy, tetrasomy 5p, tetraploidy of chromosome 9, trisomy 9p, and chromosome 21 trisomy. Studies that have identified mutations in chromone 6 displayed microdeletions or mosaicism of monosomy. Deletions in chromosome 8 (8q12.2-q21.2) and chromosome 16 (16q) were also identified, and microduplications in chromosome 17 (17p13.1) have been reported. The vast genetic influence on HC emphasizes importance of exploring and understanding the factors that confer genetic risk to improve diagnostic and prognostic efficiency. Autosomal and sex chromosomal location of all genetic findings included in this review is summarized in Figs. 3, 4.

**Table 20:** Genes of unknown function

Citation	Title	Author affiliation	Case #	Ancestry	Study design	CNS phenotype	Non-CNS phenotype	Type of hydrocephalus	Genetic methodology	Genetic analysis	Inheritance	Genetic finding
Basel-Vanagaite et al., 2010 [393]	Familial hydrocephalus with normal cognition and distinctive radiological features	Raphael Recanati Genetics Institute, Rabin Medical Center, Beilinson Campus, Petah Tikva, Israel	6 Subjects	-	Case series	Mega cisterna magna, midline cysts	Bilateral cleft lip and palate	Obstructive	TGS, cytogenetics	X-inactivation analysis, karyotyping	-	-
Bernstock et al., 2020 [394]	Complex Management of Hydrocephalus Secondary to Choroid Plexus Hyperplasia	Brigham and Women's Hospital, Harvard Medical School, Boston, Massachusetts	1 Subject	-	Case study	Developmental delay, villous hyperplasia of choroid	Hydrocele, abdominal distension, short stature, developmental delay, low-set ears, hypertelorism, deep-set eyes, down slanting palpebral fissure, and a bulbous nose	Communicating	Cytogenetics	aCGH	De novo	tetraploidy of chromosome 9
Boxill et al., 2018 [395]	Choroid plexus hyperplasia and chromosome 9p gains	Viborg Regional Hospital, Viborg, Denmark	4 Subjects	-	Case series	Choroid plexus hyperplasia	Enophthalmia, hypertelorism, downslanting palpebral fissures, broad nasal bridge, bulbous nose, downturned corners of the mouth, anomalous ears, clinodactyly, single fifth finger crease, hydrocele	Communicating	Cytogenetics	Q-banding, G-banding FISH, a-CGH	De novo	trisomy 9p
Brock et al., 2012 [396]	Mosaic tetrasomy 5p resulting from an isochromosome 5p marker chromosome: case report and review of literature	Dalhousie University, Halifax, Nova Scotia, Canada	1 Subject	Irish	Case study	Mild scoliosis, refractory siezures, global delay, hypotonia	Supernumerary nipples, transverse left palmar crease, square fingertips, bilateral 5th finger clinodactyly and shortened 4th and 5th metacarpals, overlapping toes bilaterally, skin pigmentary changes	Communicating	Cytogenetics	G-banding, FISH w/ WCP, aCGH	De novo	tetrasomy 5p
Brunetti-Pierri et al., 2008 [397]	Recurrent reciprocal 1q21.1 deletions and duplications associated with microcephaly or macrocephaly and developmental and behavioral abnormalities	Baylor College of Medicine, Houston, TX, USA	36 Subjects, 50 Controls	-	Case series	Attention deficit hyperactivity disorder autism, anxiety/depression, antisocial behavior, aggression, hallucinations	Frontal bossing, deep-set eyes and bulbous nose, hypertelorism	Communicating	Cytogenetics	aCGH, FISH	AR	1q21.2 microdeletion/microduplication
Cai et al., 2021 [398]	Classifying and Evaluating Fetuses with Ventriculomegaly in Genetic Etiologic Studies	Fujian Maternity and Child Health Hospital, Affiliated Hospital of Fujian Medical University, Fujian Key Laboratory for Prenatal Diagnosis and Birth Defect, Fuzhou, China	293 Subjects	-	Case series	Isolated hydrocephalus	Many—large study: cardiac, renal, facial agenesis, orthopaedic malformations, vascular malformations	-	WGS	SNP	*De-novo*, Maternal	Incidence of varying chromosomal abnormalities is higher in patients with non-isolated ventriculomegaly
Cambosu et al., 2013 [399]	Partial trisomy of the long arm of chromosome 1: prenatal diagnosis, clinical evaluation and cytogenetic findings. Case report and review of the literature	University of Sassari, Sassari, Italy	1 Subject	-	Case study	Macrocephaly with dolichocephaly	Prominent foreheads, modest microphthalmia, flat nasal bridge, microstomia, retrognathia, small, dysmorphic ears with small lobe and short neck, and hypoplastic left kidney	-	Cytogenetics	Q-banding, FISH	-	Partial 1q trisomy
Capra et al., 2009 [400]	Craniosynostosis, hydrocephalus, Chiari I malformation and radioulnar synostosis: probably a new syndrome	UO Neurochirurgia, Istituto G. Gaslini, Genova, Italy	2 Subjects	Caucasian, European	Case series	Sagittal craniosynostosis, chiari I malformation,	Blepharophimosis, small low-set ears, hypoplastic philtrum, radioulnar synostosis, kidney malformation, and hypogenitalism	Obstructive	Cytogenetics, TGS	Karyotyping, aCGH, MLPA	-	-
Castro-Gago et al., 2001 [401]	Congenital hydranencephalic-hydrocephalic syndrome with proliferative vasculopathy: a possible relation with mitochondrial dysfunction	Hospital Clínico Universitario, Santiago de Compostela, Spain	1 Subject	-	Case study	Severe encephalomalacia	Muscle body inclusions	-	-	-	-	-
Chen et al., 2011 [402]	Prenatal diagnosis of a de novo 17p13.1 microduplication in a fetus with ventriculomegaly and lissencephaly	Mackay Memorial Hospital, Taipei, Taiwan	1 Subject	-	Case study	Mental and motor retardation, hypotonia	Skeletal anomalies, clinodactyly of the fingers, hypertrichosis, congenital heart defects, craniofacial abnormalities such as microcephaly, down-slanting palpebral fissures, ptosis, hypertelorism, low-set malformed ears, smooth philtrum, micrognathia, high-arched palate, and a short neck	-	Cytogenetics	aCGH	De novo	17p13.1 microduplication
Chen et al., 2013 [403]	VACTERL association with hydrocephalus in a fetus conceived by in vitro fertilization and embryo transfer	Mackay Memorial Hospital, Taipei, Taiwan	1 Subject	-	Case study	Isolated hydrocephalus	Bilateral arthrogryposis, right radial aplasia, a right club hand, aplasia of the right thumb, hypoplasia of the left thumb, scoliosis, and an imperforate anus	-	Cytogenetics, TGS	aCGH	-	-
Descipio et al., 2005 [404]	Subtelomeric deletions of chromosome 6p: molecular and cytogenetic characterization of three new cases with phenotypic overlap with Ritscher-Schinzel (3C) syndrome	The Children's Hospital of Philadelphia, and The University of Pennsylvania School of Medicine, Philadelphia, Pennsylvania USA	6 Subjects, 12 Parents, Controls used	-	Case series	Dandy–Walker malformation intellectual disability	Ptosis, posterior embryotoxon, optic nerve abnormalities, mild glaucoma, atrial septal defect, patent ductus arteriosus	Communicating, Obstructive	Cytogenetics, TGS	STS mapping, FISH, direct sequencing	-	-
Dubé et al., 2000 [405]	A new association of congenital hydrocephalus, albinism, megalocornea, and retinal coloboma in a syndromic child: a clinical and genetic study	McGill University, Montreal Children's Hospital, Montreal, Quebec, Canada	1 Subject	French-Canadian	Case study	Global developmental delay, trigonocephaly	Oculocutaneous albinism, retinal coloboma, and megalocornea, prominent metopic suture, and cryptorchidism	-	Cytogenetics	FISH, karyotyping	-	-
Forcelini et al., 2006 [406]	Down syndrome with congenital hydrocephalus: case report	Rua Paissandu, Passo Fundo RS, Brazil	1 Subject	-	Case study	Isolated hydrocephalus	Upslanting)palpebral fissures; flat nasal bridge; open mouth; protruding tongue; transverse palmar creases; poor moro reflex; hyper flexibility; short stature; loose skin on nape of neck; flat facial profile; epicanthic folds; short broad hands; clinodactyly of fifth finger; gap between the first and second toes	-	Cytogenetics	-	-	Chr. 21 Trisomy
Garavelli et al., 2007 [407]	Megalencephaly and perisylvian polymicrogyria with postaxial polydactyly and hydrocephalus (MPPH): report of a new case	S. Maria Nuova Hospital, Reggio Emilia, Italy	1 Subject	-	Case study	Hypotonia, dysmorphic facial features, hypoplasia of corpus callosum	Polydactyly	-	Cytogenetics	Karyotyping, FISH, SNP	De novo	-
Inui et al., 2001 [408]	A new variant neuropathic type of Gaucher's disease characterized by hydrocephalus, corneal opacities, deformed toes, and fibrous thickening of spleen and liver capsules	Osaka University, Osaka, Japan	1 Subject	Japanese	Case study	Oculomotor apraxia, rigidity, spasticity, and hyperactive deep tendon reflexes	Corneal opacities, deformed toes, gastroesophageal reflux, and fibrous thickening of splenic and hepatic capsules	Communicating	WES	Cycle sequencing	De novo	1342G to C (D409H) homozygous mutation
Kariminejad et al., 2012 [409]	Megalencephaly-polymicrogyria-polydactyly-hydrocephalus syndrome: a case report	Kariminejad-Najmabadi Pathology and Genetics Center, Tehran, Iran	1 Subject	-	Case study	Megalocephaly, polymicrogyria, Hypotonia	Polydactyly, developmental delay, bossing forehead, long philtrum, strabismus, and mild hypertelorism	-	Cytogenetics	aCGH	-	-
Lemire et al., 2000 [410]	Chudley-McCullough syndrome: bilateral sensorineural deafness, hydrocephalus, and other structural brain abnormalities	Royal University Hospital and University of Saskatchewan, Saskatoon, Saskatchewan, Canada	2 Subjects	Canadian	Case series	Callosal dysgenesis, gray matter heterotopia, cortical dysplasia, cerebellar dysgenesis, intellectual disability	Bilateral sensorineural hearing loss, developmental delay	Obstructive	Cytogenetics, TGS	-	AR	-
Lowry et al., 2007 [411]	Absence of PITX2, BARX1, and FOXC1 mutations in De Hauwere syndrome (Axenfeld-Rieger anomaly, hydrocephaly, hearing loss): a 25-year follow up	Alberta Children's Hospital & University of Calgary, Calgary, Alberta, Canada	1 Subject	-	Case study	Isolated hydrocephalus	Short stature, hyperlaxity of joints, hearing loss	Communicating	Cytogenetics, TGS	Karyotyping, FISH	AD	-
Matteucci et al., 2006 [412]	Sensorineural deafness, hydrocephalus and structural brain abnormalities in two sisters: the Chudley-McCullough syndrome	Department of Neurosciences, University of Pisa, Pisa, Italy	2 Subjects	Italian	Case series	Callosum agenesis, interhemispheric cyst, cerebral and cerebellar abnormalities	Sensorineural hearing loss, developmental delay	Communicating	TGS, Cytogenetics	G-banding, Q-banding	AR	-
Naritomi et al., 1988 [413]	16q21 is critical for 16q deletion syndrome	School of Medicine, University of the Ryukyus, Okinawa, Japan	1 Subject	-	Case study	Hypotonia	Bossed forehead, epicanthal folds, hypertelorism, a flat, broad nasal bridge, a short nose with a bulbous tip, and low-set posteriorly rotated, deformed ears, high-arched pallet, short neck, medial toe curvature	-	Cytogenetics	G-banding	De novo	Chr. 16q deletion
Østergaard et al., 2004 [414]	Brothers with Chudley-McCullough syndrome: sensorineural deafness, agenesis of the corpus callosum, and other structural brain abnormalities	The John F. Kennedy Institute, Gl. Landevej 7, DK-2600 Glostrup, Denmark	2 Subjects	Pakistan	Case series	Callosal agenesis, colpocephaly	Bilateral sensorineural deafness	Obstructive	TGS	-	AR	-
Remes et al., 1992 [415]	Fumarase deficiency: two siblings with enlarged cerebral ventricles and polyhydramnios in utero	University of Oulu, Finland	2 Subjects, 5 Family Members, Controls	-	Case series	Hypotonia, seizures	Aciduria, dystonic tetraplegia	Communicating	-	-	AR	-
Silan et al., 2003 [416]	A new mutation of the fukutin gene in a non-Japanese patient	Abant Izzet Baysal University Duzce Medical Faculty, Duzce, Turkey	1 Subject, 2 Parents, 1 Brother	Turkish	Case study	Hypotonia, polymicrogyria in several cortical segments and severe cortical disorganization	Congenital muscular dystrophy, bilateral buphthalmus, proptosis, and cataracts	Communicating	TGS	Restriction enzyme analysis, direct sequencing	AR	9q31.2 (1 bp insertion mutation in exon 5 of the fukutin gene)
Tohyama et al., 2007 [417]	Megalencephaly and polymicrogyria with polydactyly syndrome	Kariminejad-Najmabadi Pathology and Genetics Center, Tehran, Iran	1 Subject	Japanese	Case study	Megalencephaly, hypotonia, polymicrogyria, and thin corpus callosum	Bilateral postaxial polydactyly of upper and lower limbs, developmental delay	Communicating	Cytogenetics	FISH	-	-
Toren et al., 2020 [418]	Chromosomal Microarray Evaluation of Fetal Ventriculomegaly	Sheba Medical Center, Tel Hashomer, Israel	164 Subjects, 209 Controls	-	Case series	Severity and anatomical variability in ventriculomegaly		-	Cytogenetics	Karyotyping, chromosomal microarray	De Novo, Paternal	Incidence of varying chromosomal abnormalities is higher in patients with non-isolated ventriculomegaly and bilateral ventriculomegaly
Vincent et al., 1994 [419]	A proposed new contiguous gene syndrome on 8q consists of Branchio-Oto-Renal (BOR) syndrome, Duane syndrome, a dominant form of hydrocephalus and trapeze aplasia; implications for the mapping of the BOR gene	Unité de Génétique Moléculaire Humaine (CNRS URA 1445), Institut Pasteur, Paris	1 Subject	-	Case study	Isolated hydrocephalus	Trapeze aplasia	-	Cytogenetics	Linkage analysis	De novo	8q12.2-q21.2 deletion
Wadt et al., 2012 [420]	Fetal ventriculomegaly due to familial submicroscopic terminal 6q deletions	University Hospital of Copenhagen, Rigshospitalet, Denmark	2 Subjects, 16 Family Members	-	Case series	Hypotonia	Oromotor difficulties, hypermobility, high, flat forehead, bilateral ptoses,	Obstructive	Cytogenetics	Karyotyping, aCGH, MLPA	Variable expressivity	terminal deletion of chromosome 6q
Walker et al., 1996 [421]	Prenatal diagnosis of ring chromosome 6 in a fetus with hydrocephalus	Children's Hospital Research Foundation, Cincinnati, Ohio, USA	1 Subject	-	Case study	Microcephaly, seizures	Severe bilateral hearing loss, and global development delay	Obstructive	Cytogenetics	-	De novo	chromosome 6/monosomy 6 mosaicism
Wang et al., 2020 [422]	Prenatal diagnosis of chromosomal aberrations by chromosomal microarray analysis in foetuses with ventriculomegaly	West China Second University Hospital, Sichuan University, No. 20, Sect. 3, Renminnan Road, Chengdu, 610,041, Sichuan, China	548 Subjects	-	Case series	Agenesis/hypoplasia of the corpus callosum, Dandy–Walker malformation, migration abnormality, and holoprosencephaly	Renal, cardiac, and skeletal anomalies	-	WGS, cytogenetics	SNP, Karyotyping, CNV	*De-novo*, maternal, balanced translocation	Incidence of varying chromosomal abnormalities (13 types) is higher in patients with severe ventriculomegaly
Welch et al., 2003 [423]	Chudley-McCullough syndrome: expanded phenotype and review of the literature	Gallaudet University, Washington DC, USA	3 Subjects, 13 Family Members	Western European	Case series	Obstruction of the foramen of Monro, arachnoid cyst, partial agenesis of the corpus callosum, and abnormalities in the migration of cerebellar cells	Deafness	Obstructive	TES	Linkage analysis	AR	-
Yoshioka et al., 1994 [424]	Clinical spectrum and genetic studies of Fukuyama congenital muscular dystrophy	Kobe General Hospital, Japan	48 Subjects	Japanese	Case series	Lissencephaly	Weakness, joint contractures, delayed motor development	Communicating	-	-	AR	-

Array comparative genomic hybridization (aCGH). Autosomal Dominant (AD). Autosomal Recessive (AR). Copy number variant (CNV). Fluorescence In Situ Hybridization (FISH). Fluorogenic quantitative-polymerase chain reaction (FQ-PCR). Multiplex ligation dependent probe amplification (MLPA). Next generation sequencing (NGS). Single nucleotide polymorphisms (SNP). Sequenced tagged sites (STS). Targeted exome sequencing (TES). Targeted genome sequencing (TGS). Whole chromosome probes (WCP). Whole exome sequencing (WES). Whole genome sequencing (WGS)

### Global burden of genetic hydrocephalus

**Fig. 5:**
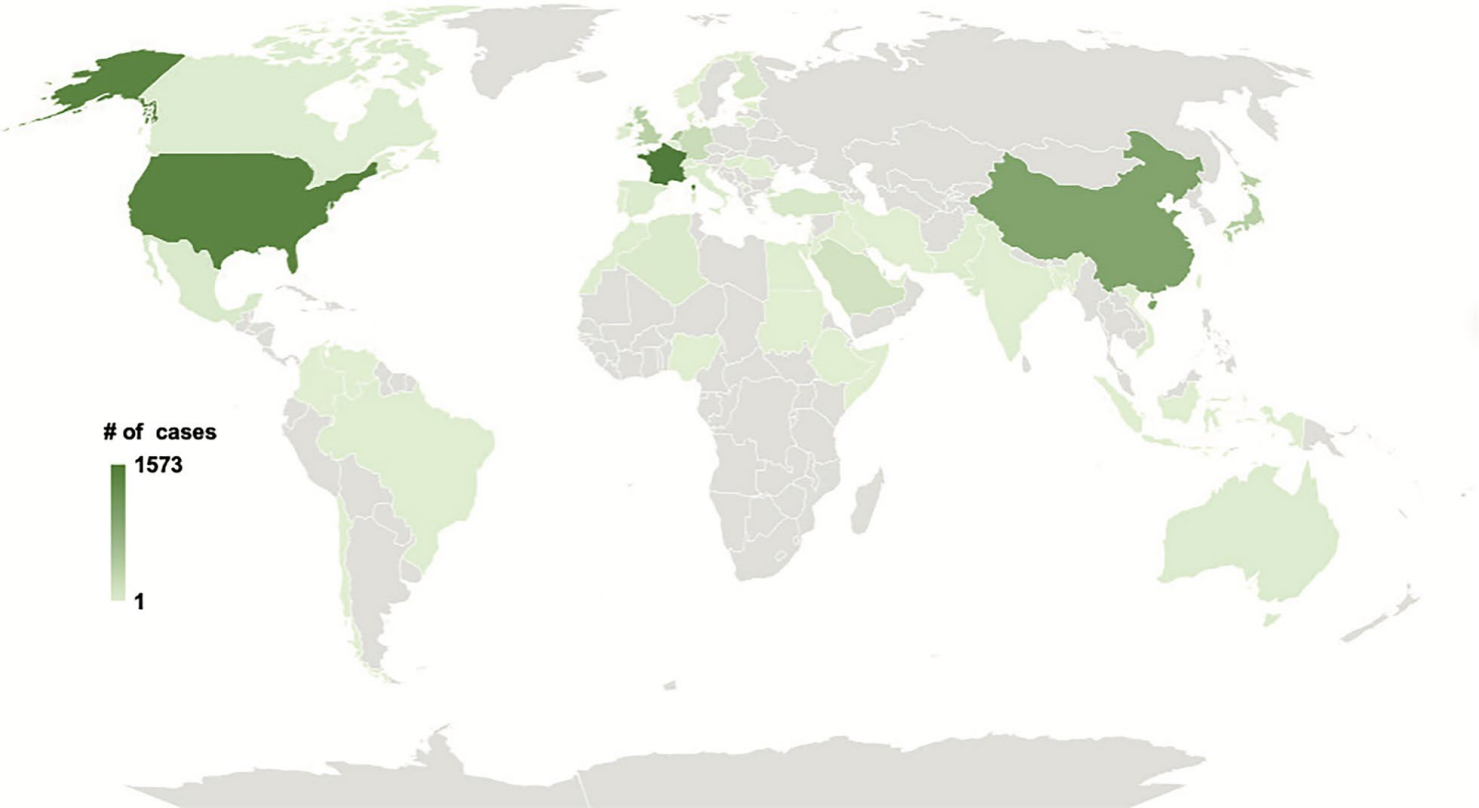
Heatmap of the globe demonstrating the country of origin for patients with genetic contributions to hydrocephalus. Figure created with OpenStreetMap

We next aimed to quantify the country of origin for patients included in this review (Fig. 5**).** Given the wide range of HC disease burden across the world [3], we aimed to determine if genetic studies of HC were similarly representative. What is clear is that for regions of the world where HC prevalence is highest (Africa, East Asia, etc.), there is an obvious lack of genetic studies of HC of any kind. For example, there is not a single study performed by authors in Sub-Saharan Africa (SSA) or including people from SSA. Given that African genomes are the most diverse and complex with generations of environmental pressures (including emerging pathogens) shaping the genome, understanding genetic risk factors in these populations is essential. While epidemiological estimates of the contribution of genetically linked forms of HC is not feasible at present, these data begin to highlight disparities in representation of genetic studies and the need for large-scale genetic studies of HC in diverse populations. These data also provide a reasonable estimate of the potential burden, although likely underestimated, contribution of genetic factors contributing to HC. It is our hope that this review highlights the diverse mechanisms underlying HC, the complex molecular pathways that may contribute to HC pathogenesis, and the need to greatly expand the representation of diverse peoples in HC genetics research.

## Discussion

HC is a complex, heterogenous condition that can be a component of a wide range of genetic conditions and can be caused by a variety of preceding environmental factors. Because HC is a component of many syndromes with a wide range of concomitant phenotypes, understanding the genetic pleiotropism of contributing genes is important for delineating the pathophysiologic basis of the disease. This review provides a broad overview of the associations between genetic mechanisms underlying HC. The variability in phenotypes observed, methodology used to uncover genetic information, and wide range of validation of genetic findings highlights the major challenges in the field. While many studies are descriptive, a wide range of hypotheses are generated based on implicated genes and potential mechanisms. Specifically, many studies implicate alterations in neurogenesis and primary brain development, as opposed to direct alterations in CSF regulation, as potential pathophysiologic mechanisms. Overall, as genomic technologies become more ubiquitous in clinical practice and more patients undergo unbiased genomic sequencing, our understanding of HC will improve. However, there are several limitations and points to consider as this field evolves.

An ongoing challenge in human genetics is proving causality of implicated genetic findings. Classical validation technique requires reproducing the implicated mutation (if evolutionarily conserved) in a model organism such as a mouse or rat. However, the physiologic regulation of CSF and mechanisms underlying brain development are markedly different in these model organisms and often do not recapitulate human disease. Many genes underlying HC are associated with other phenotypes, and it may not be possible to identify a secondary causative genetic factor that unmasks the phenotype. Since this approach relies on the gene product being evolutionarily conserved, identification of human-specific disease mechanisms is impaired. Alternative approaches to determining the relationship of a gene variant to a HC phenotype include structural biologic modeling of presumed deleterious mutations; however, this approach does not consider physiological and phenotypic heterogeneity. Similarly, these approaches often rely on protein expression in prokaryotic systems, limiting interpretation of post-translational modifications and other physiologic contributors to protein function.

Based on the significant co-occurrence of traits affecting other organ systems, it is likely that genes associated with HC display significant pleiotropy. A simplistic model of monogenic contributions to HC is unlikely to capture the genetic etiology of most cases. Even among monogenic contributions to HC, there is significant phenotypic and genetic variability (i.e., L1CAM). As quantitative genetic methodology improves to identify polygenic contributors to disease, we suspect that a much larger proportion of cases will have polygenic contributions. Because HC is a heterogenous disease, accrual of large numbers of ‘homogenous’ cases are needed to accurately quantify reproductible genetic associations.

The variability in genomic technology used to determine potential genetic contributions to HC is significant. Agnostic methods such as genome wide association studies (GWAS), transcriptome-wide association studies (TWAS), whole-exome sequencing (WES), and whole-genome sequencing (WGS) have been used, but are limited by cost, sample size, and technical expertise involved in analysis. In contrast, targeted sequencing approaches rely on hypothesis-driven identification of implicated genetic loci introducing significant experimental bias.

Our review highlights that most genetic studies of HC are performed in countries where disease burden, paradoxically, is amongst the lowest in the world. This reflects disproportionately low resources for genetic studies in low- and middle-income countries. For example, Sub Saharan Africa the most genetically diverse and complex region in the world, where the burden of HC is also the highest, yet there are no genetic studies of HC of any kind in these populations. Although the burden of HC is largely the result of infections, the genetic contributors to infection susceptibility are largely uncharacterized in these populations. Evolutionary selection pressures have been differentially shaped by exposure to infectious pathogens, geographic shifts of ancestral peoples, and population isolation. Therefore, understanding genetic factors specific to these populations is paramount to improve secondary prevention and moving towards non-surgical treatment options.

Advances in genetic technology and interpretation coupled with decreased costs will garner a new era of precision medicine that can be applied in the clinic [128]. The extent to which genetic information may guide treatment in HC has not been fully realized. As more patients are rigorously studied using complementary and convergent genomic approaches coupled with long-term clinical outcomes, we may be able to incorporate genetic information into clinical care. Owing to the genetic architecture of HC highlighted here and across many studies, we anticipate that creation of polygenic risk scores (PRS) may be the most clinically meaningful and practical for disease prognostication and understanding comorbid disease risks.

## Conclusions

HC is a phenotypically and genetically complex disease. While the literature describing the genetic causes of HC is vast, this comprehensive review highlights opportunities for further mechanistic study and disparities in ancestral representation. The varying rigor with which genetic studies are conducted highlights the challenge of determining causality of implicated genomic alterations, inadequacies of current model systems, and the need for human-specific molecular validation studies. What is clear is that our genetic understanding of HC is incomplete and our understanding of pleiotropy of implicated HC genes requires further maturation. This study represents the first large-scale systematic literature review of the genetic basis of HC in humans and highlights many areas ripe for future investigation.

## Data Availability

All data are contained within the manuscript.
